# Supplementary material for: Agency rescues competition for credit assignment among predictive cues from adverse learning conditions
Source: Sci Rep. 2021 Aug 10;11:16187. doi: 10.1038/s41598-021-95541-2 (PMC8355250; doi:10.1038/s41598-021-95541-2)
Supplement: Supplementary file 1 — Supplementary Information. [file 41598_2021_95541_MOESM1_ESM.docx]

**Supplementary Materials for**

**Agency rescues competition for credit assignment among predictive cues from adverse learning conditions**

**Mihwa Kang, Ingrid Reverte, Stephen Volz, Keith Kaufman, Salvatore Fevola, Anna Matarazzo, Fahd H. Alhazmi, Inmaculada Marquez, Mihaela D. Iordanova, Guillem R. Esber**

Corresponding author: Guillem R. Esber ([GEsber@brooklyn.cuny.edu](mailto:GEsber@brooklyn.cuny.edu))

**This PDF file includes**

Supplementary tables

Experiments S1, S2

Simulations of an agency-related positivity bias in prediction errors

Statistical tables (Jamovi)

**Supplementary tables**

| **Exp.** | **Phase** | **Total # trials** | **Mean ITI** | **SD ITI** | **Mean session** | **SD session** |
| --- | --- | --- | --- | --- | --- | --- |
| 1 | Pretraining | 96 | 18.0 s | 6.7 s | 50. 2 min | 10.7 min |
|  | Compound | 96 (100 w/ probes) | 13.1 s | 3.4 s | 43.1 min | 5.6 min |
| 2 | Pretraining | 96 | 19.5 s | 5.8 s | 52.5 min | 9.3 min |
|  | Compound | 100 | 18.7 s | 8.5 s | 51.2 min | 13.7 min |
| 3 | Pretraining | 96 | 24.3 s | 7.8 s | 60.1 min | 12.5 min |
|  | Compound | 96 (100 w/ probes) | 18.1 s | 7.5 s | 51.3 min | 12.2 min |
| 4 | Patterning | 96 | 13.4 s | 2.7 s | 42.7 min | 4.3 min |
| S2 | Patterning | 96 | 13.0 s | 3.6 s | 42.1 min | 5.8 min |

*Table S1.* Means and standard deviations of the effective intertrial intervals (ITI) and session durations for each experiment and training phase. The effective ITI takes into account not only the programmed ITIs, but also additional between-trial periods introduced by forgone trial offers (trials that were not initiated by Agency rats). Incomplete sessions that timed out after 90 min were excluded from these calculations.

| **Exp.** | **Phase** | **Session** | **Agency rat** | **Passive rat** | **Completed trials** |
| --- | --- | --- | --- | --- | --- |
| 1 | Pretraining | 8 | 5 | 6 | 27/96 |
|  |  | 2 | 3 | 4 | 72/96 |
|  |  | 2 | 5 | 6 | 92/96 |
| 2 | Pretraining | 1 | 11 | 12 | 68/96 |
|  |  | 2 | 11 | 12 | 84/96 |
|  |  | 1 | 3 | 4 | 84/96 |
|  |  | 3 | 11 | 12 | 81/100 |
| 3 | Pretraining | 1 | 11 | 12 | 32/96 |
|  |  | 2 | 11 | 12 | 33/96 |
|  |  | 3 | 11 | 12 | 42/96 |
|  |  | 5 | 13 | 14 | 66/96 |
|  |  | 1 | 15 | 16 | 85/96 |
|  |  | 1 | 3 | 4 | 89/96 |
|  | Compound | 1 | 1 | 2 | 10/96 |
|  |  | 2 | 1 | 2 | 10/96 |
|  |  | 1 | 11 | 12 | 74/96 |
|  |  | 1 | 13 | 14 | 86/96 |
|  |  | 15 | 11 | 12 | 82/100 |
|  |  | 14 | 11 | 12 | 90/100 |
|  |  | 13 | 5 | 6 | 96/100 |
|  |  | 24 | 15 | 16 | 96/100 |
|  |  | 13 | 15 | 16 | 96/100 |
|  |  | 13 | 17 | 18 | 96/100 |
|  |  | 13 | 7 | 8 | 96/100 |
|  |  | 13 | 11 | 12 | 96/100 |
|  |  | 13 | 3 | 4 | 96/100 |
|  |  | 13 | 13 | 14 | 96/100 |
|  |  | 31 | 1 | 2 | 99/100 |
|  |  | 14 | 13 | 14 | 99/100 |
| S2 | Patterning | 25 | 3 | 4 | 24/96 |

*Table S2.* List of sessions that timed out after 90 min before the rats could complete all scheduled trials. Notice that when an Agency rat failed to complete a session, its yoked counterpart in the Passive group was also affected. The ID numbers of all rats concerned are shown for each experiment and training phase, along with the number of trials they completed.

**Exp. S1: Piloting a novel cue-competition task in a standard Pavlovian magazine-approach setting**

The purpose of this study was to pilot the novel cue-competition design to be used in the second study in a standard Pavlovian magazine-approach setting. Trials were spaced out by a mean intertrial interval of 60 s. For ease of reference, the experimental design can be found in Fig. S1A.

**Results**

The pretraining phase consisted of 10 sessions of discrimination training with A(1), B(0), X(.75) and Y(.25) (Fig S1B, left). A Cue x Session block repeated measures ANOVA revealed a main effect of cue (*F*_(3)_ = 34.50 , *p* < 0.001) and session block (*F*_(4)_ = 11.79 , *p* < 0.001), and a cue by session block interaction (*F*_(12)_ = 4.79 , *p* < 0.001). Bonferroni-corrected post-hoc analyses of this interaction revealed that responding to A(1) was significantly greater than to B(0) on session blocks 2, 4 and 5 (*t*_(7)_ = [5.32 – 7.02], *p* < 0.010), while responding to X(.75) was marginally greater than to Y(.25) on session blocks 4 and 5 (*t*_(7)_ = [3.97 – 4.09], *p* = 0.05).

The results of the second compound phase, which comprised 20 sessions, are shown in Fig. S1, panel B (right). Competitive cue interactions were evidenced by a gradual switch in responding to X and Y; that is, a decrease in responding to X (due to competition with A) combined with an increase in responding to Y (due to protection from extinction by B). In support of this impression, an ANOVA on responding to X and Y throughout this phase revealed no main effect of cue or session block, but a significant cue by session block interaction (F_(9)_ = 4.45; *p* < 0.001). Bonferroni-corrected post hoc analyses revealed, however, no significant difference between X and Y on any given session block. A further ANOVA on the remainder of the cues presented in the compound phase revealed a significant main effect of cue (*F_(_*_3)_ = 17.11; *p* < 0.001) and a cue by session block interaction (*F*_(27)_ = 2.54; *p* < 0.001). Bonferroni-corrected post hoc analyses of this interaction revealed significant differences between A(1) and B(0) on session blocks 1, 3 and 4 (*t*_(7)_ = [4.60 – 4.89], *p* < 0.04) and between 3AX(1) and 3BY(0) on session block 5 (*t*_(7)_ = 4.83; *p* = 0.04).


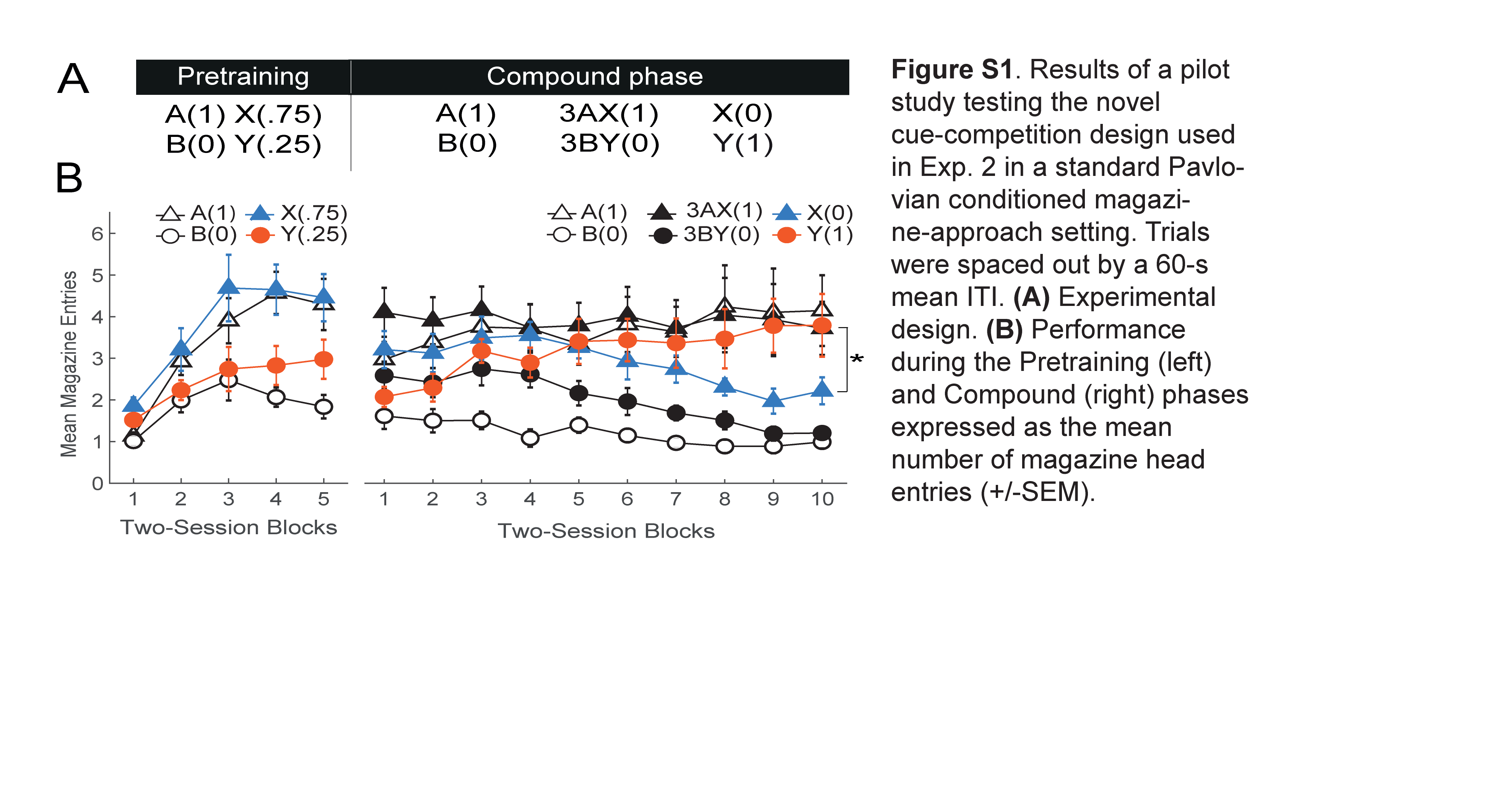


**Materials and Methods**

**Subjects**

The subjects were 8 naïve Long-Evans rats. At the start of the experiment they were ~22 wo and weighed 470-527 g (males) and 291-319 g (females). Husbandry details were the same as described in the main text.

**Apparatus**

Same as described in the main text.

**Procedure**

Rats were magazine trained in the manner described in the main text before receiving standard Pavlovian conditioned magazine-approach training. The experimental design was the same as that shown in Fig. 2A. All procedural details were identical to those used in the Passive group of Exp. 2 with two exceptions. First, trials were not preceded by the trial-availability cue (noseport light) and second, the mean ITI was 60 s (range: 30-90s)

**Experiment S2: Ruling out alternatives for the role of agency in competitive credit assignment – Replication**

The purpose of Exp. S2 was to replicate the findings of the patterning study in rats that had previously exhibited different degrees of competitive credit assignment. The same Agency and Passive groups used in the blocking task (Exp. 1) went on to receive negative- and positive-patterning problems.

**Results**

Inspection of Fig. S2 suggests that the same Passive rats that showed impaired competitive credit assignment during blocking training were, if anything, better able to solve the complex non-linear discriminations. Once again, we averaged responding on elemental trials to simplify the analysis [mean of A(1) and X(1) trials and of B(0) and Y(0) trials]. A Group x Session block x Cue mixed ANOVA on the negative-patterning [A(1)/X(1) vs. AX(0)] discrimination revealed an effect of cue [*F*_(1,350)_ = 53.37, *p* < 0.001] as well as significant cue by session block [*F*_(12,350)_ = 2.31, *p* = 0.008] and group by cue interactions [*F*_(1,350)_ = 9.11, *p* = 0.003]. Bonferroni-corrected simple main effects analysis of the latter interaction revealed that both groups solved this discrimination [Agency: *t*_(350)_ = -3.03, *p* = 0.006; Passive: *t*_(350)_ = -7.30, p < 0.002], although Passive rats showed a larger effect size than Agency rats (Cohen’s d = 0.64 and 0.18, respectively). A parallel analysis of the positive-patterning [B(0)/Z(0) vs. BZ(1)] discrimination revealed a main effect of cue (elements vs. compound) [*F*_(1,350)_ = 172.85, *p* < 0.001] and a cue by group interaction [*F*_(1,350)_ = 20.08, *p* < 0.001]. Bonferroni-corrected simple main effects analyses confirmed that both groups also solved this discrimination [Agency: *t*_(350)_ = 6.13, p < 0.002; Passive: *t*_(350)_ = 12.47 , p < 0.002], but, once again, a larger effect was observed in Passive than Agency rats (Cohen’s d = 1.50 and 0.48, respectively). These findings bolster the hypothesis that the deficits observed in Passive relative to Agency rats in Exps. 1-3 were specific to cue competition and not the result of impaired processing of compound cues or more general impairments in discrimination learning.


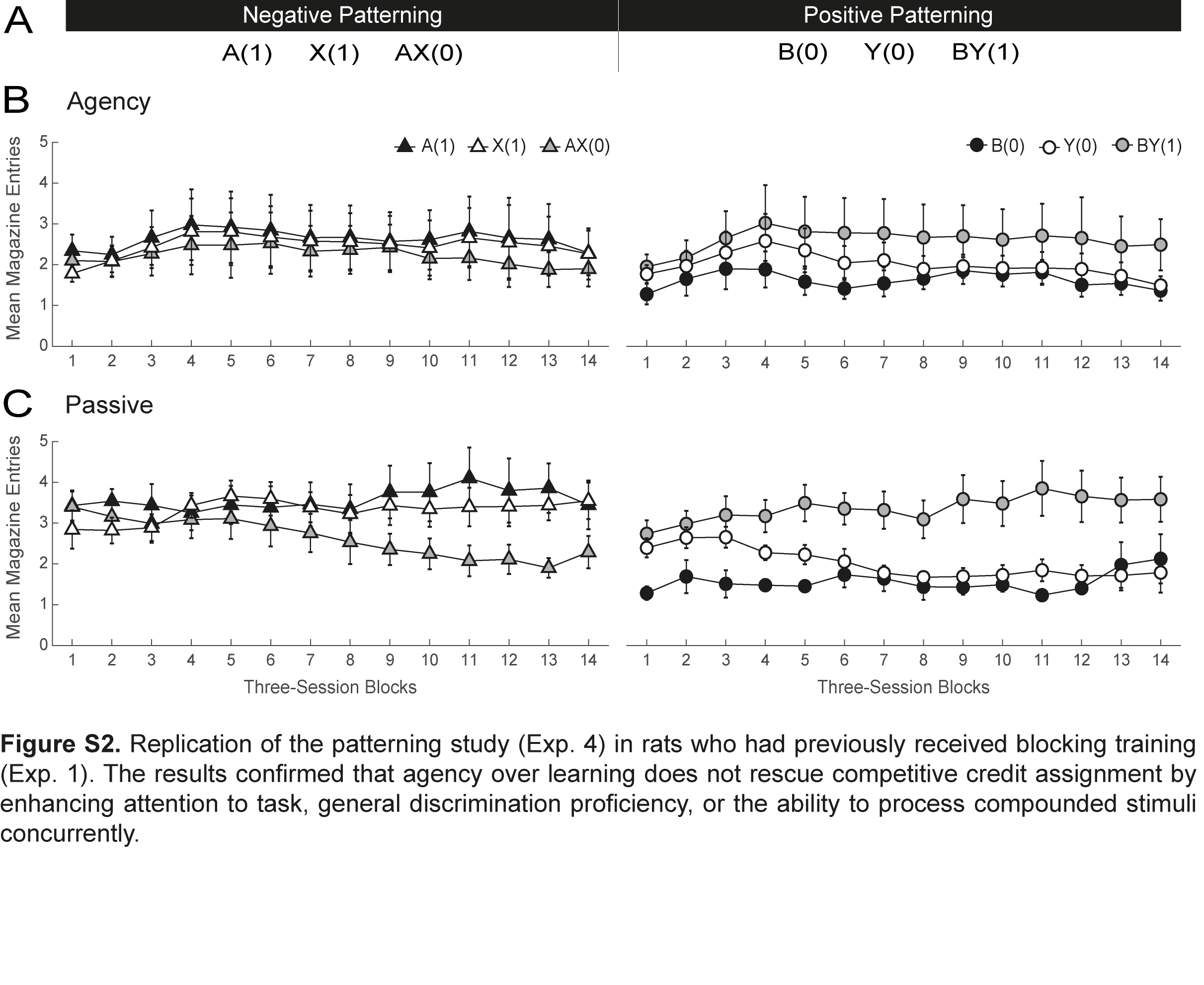


**Materials and Methods**

**Subjects**

The subjects were the 16 Long-Evans rats that previously took part in the blocking study (Exp. 1). At the outset of the study they were ~26 wo and weighed 544-601 g (males) and 303-340 g (females). Husbandry details were the same as described in the main text.

**Apparatus**

Same as described in the main text.

**Procedure**

Rats did not require magazine nor shaping training, as they had already received such training at the outset of Exp. 1. The experimental design as well as all other procedures was identical to that used in the patterning study (Exp. 4), with the exception that novel auditory stimuli were used in the role of X and Y (a 2.5-Hz, 80-dB clicker and a 1-kHz, 80-dB tone, counterbalanced). Thirty-nine training sessions were conducted.

**Simulations of an agency-related positivity bias in prediction errors**


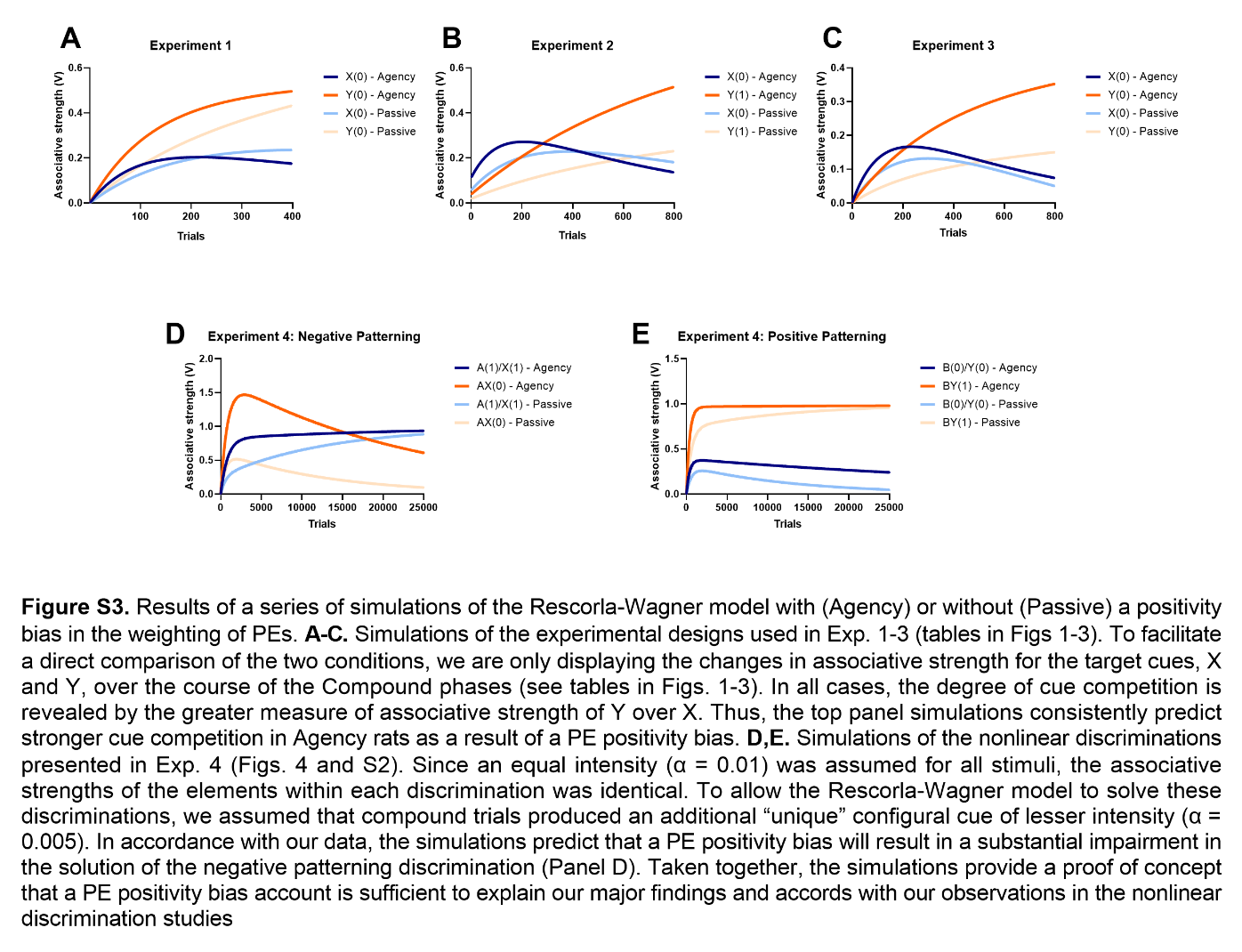
To assess whether the more competitive credit assignment observed in Agency rats resulted from their weighting positive prediction errors (+PE) significantly more than negative ones (-PE) (i.e., a positivity bias), we conducted a series of simulations using the Rescorla-Wagner model, one for each of our experimental designs. In this model, the associative change undergone by a cue on any given trial equals the product of the PE and two learning rate parameters, α and β, which represent the intensity of the cue and the outcome, respectively. In our simulations, we used β to specify the relative weights of +PEs and -PEs. Specifically, we implemented an agency-induced positivity bias by multiplying +PEs by a larger β than -PEs (+PE: β = 0.1; -PE: β = 0.01). For the Passive condition, we adopted the same β value for both +PEs and -PEs (β = 0.05). The intensity of all cues (α) was set at 0.01, with the exception noted in the figure caption. Whenever applicable, training of the model in the Pretraining phase was preasymptotic (not shown).

**Statistical analyses (Jamovi output tables)**

**Experiment 1: Agency rescues the blocking effect from the deleterious effects of massed training**

**Pretraining phase**

| Fixed Effect Omnibus tests | | | | | | | | | |
| --- | --- | --- | --- | --- | --- | --- | --- | --- | --- |
|  |  |  |  |  |  |  |  |  |  |
|  | | **F** | | **Num df** | | **Den df** | | **p** | |
| Group |  | 8.098 |  | 1 |  | 14.0 |  | 0.013 |  |
| 2 SessionBlocks |  | 0.504 |  | 6 |  | 182.0 |  | 0.805 |  |
| Stimulus |  | 398.237 |  | 1 |  | 182.0 |  | < .001 |  |
| Group ✻ 2 SessionBlocks |  | 0.168 |  | 6 |  | 182.0 |  | 0.985 |  |
| Group ✻ Stimulus |  | 15.647 |  | 1 |  | 182.0 |  | < .001 |  |
| 2 SessionBlocks ✻ Stimulus |  | 9.770 |  | 6 |  | 182.0 |  | < .001 |  |
| Group ✻ 2 SessionBlocks ✻ Stimulus |  | 0.267 |  | 6 |  | 182.0 |  | 0.952 |  |
| Note. Satterthwaite method for degrees of freedom | | | | | | | | | |
|  | | | | | | | | | |

| Post Hoc Comparisons - Group ✻ Stimulus | | | | | | | | | | | | | | | | | | | |
| --- | --- | --- | --- | --- | --- | --- | --- | --- | --- | --- | --- | --- | --- | --- | --- | --- | --- | --- | --- |
| **Comparison** | | | | | | | | | |  | | | | | | | | | |
| **Group** | | **Stimulus** | |  | | **Group** | | **Stimulus** | | **Difference** | | **SE** | | **t** | | **df** | | **p_bonferroni_** | |
| 2 |  | A+ |  | - |  | 2 |  | B- |  | 2.703 |  | 0.160 |  | 16.91 |  | 182.0 |  | < .001 |  |
| 2 |  | A+ |  | - |  | 1 |  | B- |  | 3.222 |  | 0.358 |  | 9.00 |  | 17.3 |  | < .001 |  |
| 1 |  | B- |  | - |  | 2 |  | B- |  | -0.519 |  | 0.358 |  | -1.45 |  | 17.3 |  | 0.989 |  |
| 1 |  | A+ |  | - |  | 2 |  | B- |  | 1.290 |  | 0.358 |  | 3.60 |  | 17.3 |  | 0.013 |  |
| 1 |  | A+ |  | - |  | 2 |  | A+ |  | -1.413 |  | 0.358 |  | -3.95 |  | 17.3 |  | 0.006 |  |
| 1 |  | A+ |  | - |  | 1 |  | B- |  | 1.809 |  | 0.160 |  | 11.31 |  | 182.0 |  | < .001 |  |
| **Compound phase (excluding X and Y probes)** | | | | | | | | | | | | | | | | | | | |

| Fixed Effect Omnibus tests | | | | | | | | | |
| --- | --- | --- | --- | --- | --- | --- | --- | --- | --- |
|  |  |  |  |  |  |  |  |  |  |
|  | | **F** | | **Num df** | | **Den df** | | **p** | |
| Group |  | 7.066 |  | 1 |  | 14.0 |  | 0.019 |  |
| 4Session Blocks |  | 4.280 |  | 4 |  | 266.0 |  | 0.002 |  |
| Stimulus |  | 187.346 |  | 3 |  | 266.0 |  | < .001 |  |
| Group ✻ 4Session Blocks |  | 1.757 |  | 4 |  | 266.0 |  | 0.138 |  |
| Group ✻ Stimulus |  | 10.330 |  | 3 |  | 266.0 |  | < .001 |  |
| 4Session Blocks ✻ Stimulus |  | 0.220 |  | 12 |  | 266.0 |  | 0.997 |  |
| Group ✻ 4Session Blocks ✻ Stimulus |  | 0.184 |  | 12 |  | 266.0 |  | 0.999 |  |
| Note. Satterthwaite method for degrees of freedom | | | | | | | | | |
|  | | | | | | | | | |

| Post Hoc Comparisons - Group ✻ Stimulus | | | | | | | | | | | | | | | | | | | |
| --- | --- | --- | --- | --- | --- | --- | --- | --- | --- | --- | --- | --- | --- | --- | --- | --- | --- | --- | --- |
| **Comparison** | | | | | | | | | |  | | | | | | | | | |
| **Group** | | **Stimulus** | |  | | **Group** | | **Stimulus** | | **Difference** | | **SE** | | **t** | | **df** | | **p_bonferroni_** | |
| 2 |  | B- |  | - |  | 2 |  | BY+ |  | -2.9764 |  | 0.181 |  | -16.4061 |  | 266.0 |  | < .001 |  |
| 2 |  | B- |  | - |  | 1 |  | BY+ |  | -1.5457 |  | 0.493 |  | -3.1357 |  | 17.3 |  | 0.166 |  |
| 2 |  | A+ |  | - |  | 2 |  | B- |  | 3.0748 |  | 0.181 |  | 16.9485 |  | 266.0 |  | < .001 |  |
| 2 |  | A+ |  | - |  | 2 |  | BY+ |  | 0.0984 |  | 0.181 |  | 0.5424 |  | 266.0 |  | 1.000 |  |
| 2 |  | A+ |  | - |  | 2 |  | AX+ |  | -0.0520 |  | 0.181 |  | -0.2865 |  | 266.0 |  | 1.000 |  |
| 2 |  | A+ |  | - |  | 1 |  | B- |  | 3.4519 |  | 0.493 |  | 7.0027 |  | 17.3 |  | < .001 |  |
| 2 |  | A+ |  | - |  | 1 |  | BY+ |  | 1.5291 |  | 0.493 |  | 3.1021 |  | 17.3 |  | 0.178 |  |
| 2 |  | A+ |  | - |  | 1 |  | AX+ |  | 1.5901 |  | 0.493 |  | 3.2257 |  | 17.3 |  | 0.136 |  |
| 2 |  | AX+ |  | - |  | 2 |  | B- |  | 3.1268 |  | 0.181 |  | 17.2350 |  | 266.0 |  | < .001 |  |
| 2 |  | AX+ |  | - |  | 2 |  | BY+ |  | 0.1504 |  | 0.181 |  | 0.8289 |  | 266.0 |  | 1.000 |  |
| 2 |  | AX+ |  | - |  | 1 |  | B- |  | 3.5039 |  | 0.493 |  | 7.1082 |  | 17.3 |  | < .001 |  |
| 2 |  | AX+ |  | - |  | 1 |  | BY+ |  | 1.5811 |  | 0.493 |  | 3.2076 |  | 17.3 |  | 0.142 |  |
| 1 |  | B- |  | - |  | 2 |  | B- |  | -0.3770 |  | 0.493 |  | -0.7649 |  | 17.3 |  | 1.000 |  |
| 1 |  | B- |  | - |  | 2 |  | BY+ |  | -3.3535 |  | 0.493 |  | -6.8031 |  | 17.3 |  | < .001 |  |
| 1 |  | B- |  | - |  | 1 |  | BY+ |  | -1.9227 |  | 0.181 |  | -10.5982 |  | 266.0 |  | < .001 |  |
| 1 |  | BY+ |  | - |  | 2 |  | BY+ |  | -1.4307 |  | 0.493 |  | -2.9025 |  | 17.3 |  | 0.273 |  |
| 1 |  | A+ |  | - |  | 2 |  | B- |  | 1.5568 |  | 0.493 |  | 3.1583 |  | 17.3 |  | 0.158 |  |
| 1 |  | A+ |  | - |  | 2 |  | BY+ |  | -1.4196 |  | 0.493 |  | -2.8799 |  | 17.3 |  | 0.287 |  |
| 1 |  | A+ |  | - |  | 2 |  | A+ |  | -1.5180 |  | 0.493 |  | -3.0796 |  | 17.3 |  | 0.187 |  |
| 1 |  | A+ |  | - |  | 2 |  | AX+ |  | -1.5700 |  | 0.493 |  | -3.1850 |  | 17.3 |  | 0.149 |  |
| 1 |  | A+ |  | - |  | 1 |  | B- |  | 1.9339 |  | 0.181 |  | 10.6595 |  | 266.0 |  | < .001 |  |
| 1 |  | A+ |  | - |  | 1 |  | BY+ |  | 0.0111 |  | 0.181 |  | 0.0612 |  | 266.0 |  | 1.000 |  |
| 1 |  | A+ |  | - |  | 1 |  | AX+ |  | 0.0720 |  | 0.181 |  | 0.3971 |  | 266.0 |  | 1.000 |  |
| 1 |  | AX+ |  | - |  | 2 |  | B- |  | 1.4848 |  | 0.493 |  | 3.0121 |  | 17.3 |  | 0.216 |  |
| 1 |  | AX+ |  | - |  | 2 |  | BY+ |  | -1.4917 |  | 0.493 |  | -3.0261 |  | 17.3 |  | 0.210 |  |
| 1 |  | AX+ |  | - |  | 2 |  | AX+ |  | -1.6420 |  | 0.493 |  | -3.3312 |  | 17.3 |  | 0.108 |  |
| 1 |  | AX+ |  | - |  | 1 |  | B- |  | 1.8618 |  | 0.181 |  | 10.2623 |  | 266.0 |  | < .001 |  |
| 1 |  | AX+ |  | - |  | 1 |  | BY+ |  | -0.0609 |  | 0.181 |  | -0.3359 |  | 266.0 |  | 1.000 |  |
|  | | | | | | | | | | | | | | | | | | | |

**Compound phase (probe trials X and Y across last 4 sessions)**

| Fixed Effect Omnibus tests | | | | | | | | | |
| --- | --- | --- | --- | --- | --- | --- | --- | --- | --- |
|  |  |  |  |  |  |  |  |  |  |
|  | | **F** | | **Num df** | | **Den df** | | **p** | |
| Group |  | 6.422 |  | 1 |  | 14.0 |  | 0.024 |  |
| Session |  | 0.530 |  | 3 |  | 98.0 |  | 0.663 |  |
| Stimulus |  | 7.799 |  | 1 |  | 98.0 |  | 0.006 |  |
| Group ✻ Session |  | 0.147 |  | 3 |  | 98.0 |  | 0.931 |  |
| Group ✻ Stimulus |  | 6.281 |  | 1 |  | 98.0 |  | 0.014 |  |
| Session ✻ Stimulus |  | 1.896 |  | 3 |  | 98.0 |  | 0.135 |  |
| Group ✻ Session ✻ Stimulus |  | 0.993 |  | 3 |  | 98.0 |  | 0.399 |  |
| Note. Satterthwaite method for degrees of freedom | | | | | | | | | |
|  | | | | | | | | | |

**Simple Effects**

| Simple effects of Stimulus : Parameter estimates | | | | | | | | | | | | | | | | | |
| --- | --- | --- | --- | --- | --- | --- | --- | --- | --- | --- | --- | --- | --- | --- | --- | --- | --- |
| **Moderator levels** | |  | | | | | | **95% Confidence Interval** | | | |  | | | | | |
| **Group** | | **contrast** | | **Estimate** | | **SE** | | **Lower** | | **Upper** | | **df** | | **t** | | **p** | |
| 1 |  | Y- - X- |  | 1.1562 |  | 0.309 |  | 0.544 |  | 1.769 |  | 98.0 |  | 3.747 |  | < .001 |  |
| 2 |  | Y- - X- |  | 0.0625 |  | 0.309 |  | -0.550 |  | 0.675 |  | 98.0 |  | 0.203 |  | 0.840 |  |
| Note. Simple effects are estimated keeping constant other independent variable(s) in the model | | | | | | | | | | | | | | | | | |
|  | | | | | | | | | | | | | | | | | |

**Experiment 2: Agency rescues competitive credit assignment in a novel cue competition task**

**Pretraining phase**

| Fixed Effect Omnibus tests | | | | | | | | | |
| --- | --- | --- | --- | --- | --- | --- | --- | --- | --- |
|  |  |  |  |  |  |  |  |  |  |
|  | | **F** | | **Num df** | | **Den df** | | **p** | |
| group |  | 0.122 |  | 1 |  | 14.0 |  | 0.732 |  |
| 2 SessionBlock |  | 2.449 |  | 4 |  | 266.0 |  | 0.047 |  |
| Stimulus |  | 24.271 |  | 3 |  | 266.0 |  | < .001 |  |
| group ✻ 2 SessionBlock |  | 0.382 |  | 4 |  | 266.0 |  | 0.821 |  |
| group ✻ Stimulus |  | 1.843 |  | 3 |  | 266.0 |  | 0.140 |  |
| 2 SessionBlock ✻ Stimulus |  | 2.030 |  | 12 |  | 266.0 |  | 0.022 |  |
| group ✻ 2 SessionBlock ✻ Stimulus |  | 0.118 |  | 12 |  | 266.0 |  | 1.000 |  |
|  | | | | | | | | | |

| Post Hoc Comparisons - 2 SessionBlock ✻ Stimulus | | | | | | | | | | | | | | | | | | | | | | |  |
| --- | --- | --- | --- | --- | --- | --- | --- | --- | --- | --- | --- | --- | --- | --- | --- | --- | --- | --- | --- | --- | --- | --- | --- |
| **Comparison** | | | | | | | | | |  | | | | | | | | | | | | |  |
| **2 SessBlock** | | **Stimulus** | |  | | **2 SessBlock** | | **Stimulus** | | **Difference** | | **SE** | **t** | | | | | **df** | | **p** | | |  |
| 2 |  | B- |  | - |  | 2 |  | X+/X- |  | -0.98698 |  | 0.371 | |  | -2.6627 |  | 266 | |  | | 0.008 |  | |
| 2 |  | B- |  | - |  | 2 |  | Y-/Y+ |  | -0.29687 |  | 0.371 | |  | -0.8009 |  | 266 | |  | | 0.424 |  | |
| 2 |  | B- |  | - |  | 1 |  | X+/X- |  | -0.27892 |  | 0.371 | |  | -0.7525 |  | 266 | |  | | 0.452 |  | |
| 2 |  | B- |  | - |  | 1 |  | Y-/Y+ |  | -0.13128 |  | 0.371 | |  | -0.3542 |  | 266 | |  | | 0.723 |  | |
| 2 |  | B- |  | - |  | 3 |  | B- |  | 0.11328 |  | 0.371 | |  | 0.3056 |  | 266 | |  | | 0.760 |  | |
| 2 |  | B- |  | - |  | 3 |  | X+/X- |  | -0.93620 |  | 0.371 | |  | -2.5257 |  | 266 | |  | | 0.012 |  | |
| 2 |  | B- |  | - |  | 3 |  | Y-/Y+ |  | 0.35286 |  | 0.371 | |  | 0.9520 |  | 266 | |  | | 0.342 |  | |
| 2 |  | B- |  | - |  | 4 |  | B- |  | 0.39323 |  | 0.371 | |  | 1.0609 |  | 266 | |  | | 0.290 |  | |
| 2 |  | B- |  | - |  | 4 |  | X+/X- |  | -0.86198 |  | 0.371 | |  | -2.3254 |  | 266 | |  | | 0.021 |  | |
| 2 |  | B- |  | - |  | 4 |  | Y-/Y+ |  | 0.27083 |  | 0.371 | |  | 0.7307 |  | 266 | |  | | 0.466 |  | |
| 2 |  | B- |  | - |  | 5 |  | B- |  | 0.42318 |  | 0.371 | |  | 1.1416 |  | 266 | |  | | 0.255 |  | |
| 2 |  | B- |  | - |  | 5 |  | X+/X- |  | -0.59245 |  | 0.371 | |  | -1.5983 |  | 266 | |  | | 0.111 |  | |
| 2 |  | B- |  | - |  | 5 |  | Y-/Y+ |  | 0.49870 |  | 0.371 | |  | 1.3454 |  | 266 | |  | | 0.180 |  | |
| 2 |  | A+ |  | - |  | 2 |  | B- |  | 0.99349 |  | 0.371 | |  | 2.6802 |  | 266 | |  | | 0.008 |  | |
| 2 |  | A+ |  | - |  | 2 |  | X+/X- |  | 0.00651 |  | 0.371 | |  | 0.0176 |  | 266 | |  | | 0.986 |  | |
| 2 |  | A+ |  | - |  | 2 |  | Y-/Y+ |  | 0.69661 |  | 0.371 | |  | 1.8793 |  | 266 | |  | | 0.061 |  | |
| 2 |  | A+ |  | - |  | 1 |  | B- |  | 1.00986 |  | 0.371 | |  | 2.7244 |  | 266 | |  | | 0.007 |  | |
| 2 |  | A+ |  | - |  | 1 |  | X+/X- |  | 0.71457 |  | 0.371 | |  | 1.9278 |  | 266 | |  | | 0.055 |  | |
| 2 |  | A+ |  | - |  | 1 |  | Y-/Y+ |  | 0.86221 |  | 0.371 | |  | 2.3261 |  | 266 | |  | | 0.021 |  | |
| 2 |  | A+ |  | - |  | 3 |  | B- |  | 1.10677 |  | 0.371 | |  | 2.9858 |  | 266 | |  | | 0.003 |  | |
| 2 |  | A+ |  | - |  | 3 |  | A+ |  | -0.38932 |  | 0.371 | |  | -1.0503 |  | 266 | |  | | 0.295 |  | |
| 2 |  | A+ |  | - |  | 3 |  | X+/X- |  | 0.05729 |  | 0.371 | |  | 0.1546 |  | 266 | |  | | 0.877 |  | |
| 2 |  | A+ |  | - |  | 3 |  | Y-/Y+ |  | 1.34635 |  | 0.371 | |  | 3.6322 |  | 266 | |  | | < .001 |  | |
| 2 |  | A+ |  | - |  | 4 |  | B- |  | 1.38672 |  | 0.371 | |  | 3.7411 |  | 266 | |  | | < .001 |  | |
| 2 |  | A+ |  | - |  | 4 |  | A+ |  | -0.33333 |  | 0.371 | |  | -0.8993 |  | 266 | |  | | 0.369 |  | |
| 2 |  | A+ |  | - |  | 4 |  | X+/X- |  | 0.13151 |  | 0.371 | |  | 0.3548 |  | 266 | |  | | 0.723 |  | |
| 2 |  | A+ |  | - |  | 4 |  | Y-/Y+ |  | 1.26432 |  | 0.371 | |  | 3.4109 |  | 266 | |  | | < .001 |  | |
| 2 |  | A+ |  | - |  | 5 |  | B- |  | 1.41667 |  | 0.371 | |  | 3.8219 |  | 266 | |  | | < .001 |  | |
| 2 |  | A+ |  | - |  | 5 |  | A+ |  | 0.01562 |  | 0.371 | |  | 0.0422 |  | 266 | |  | | 0.966 |  | |
| 2 |  | A+ |  | - |  | 5 |  | X+/X- |  | 0.40104 |  | 0.371 | |  | 1.0819 |  | 266 | |  | | 0.280 |  | |
| 2 |  | A+ |  | - |  | 5 |  | Y-/Y+ |  | 1.49219 |  | 0.371 | |  | 4.0256 |  | 266 | |  | | < .001 |  | |
| 2 |  | X+/X- |  | - |  | 2 |  | Y-/Y+ |  | 0.69010 |  | 0.371 | |  | 1.8618 |  | 266 | |  | | 0.064 |  | |
| 2 |  | X+/X- |  | - |  | 1 |  | Y-/Y+ |  | 0.85570 |  | 0.371 | |  | 2.3085 |  | 266 | |  | | 0.022 |  | |
| 2 |  | X+/X- |  | - |  | 3 |  | X+/X- |  | 0.05078 |  | 0.371 | |  | 0.1370 |  | 266 | |  | | 0.891 |  | |
| 2 |  | X+/X- |  | - |  | 3 |  | Y-/Y+ |  | 1.33984 |  | 0.371 | |  | 3.6146 |  | 266 | |  | | < .001 |  | |
| 2 |  | X+/X- |  | - |  | 4 |  | X+/X- |  | 0.12500 |  | 0.371 | |  | 0.3372 |  | 266 | |  | | 0.736 |  | |
| 2 |  | X+/X- |  | - |  | 4 |  | Y-/Y+ |  | 1.25781 |  | 0.371 | |  | 3.3933 |  | 266 | |  | | < .001 |  | |
| 2 |  | X+/X- |  | - |  | 5 |  | X+/X- |  | 0.39453 |  | 0.371 | |  | 1.0644 |  | 266 | |  | | 0.288 |  | |
| 2 |  | X+/X- |  | - |  | 5 |  | Y-/Y+ |  | 1.48568 |  | 0.371 | |  | 4.0081 |  | 266 | |  | | < .001 |  | |
| 2 |  | Y-/Y+ |  | - |  | 3 |  | Y-/Y+ |  | 0.64974 |  | 0.371 | |  | 1.7529 |  | 266 | |  | | 0.081 |  | |
| 2 |  | Y-/Y+ |  | - |  | 4 |  | Y-/Y+ |  | 0.56771 |  | 0.371 | |  | 1.5316 |  | 266 | |  | | 0.127 |  | |
| 2 |  | Y-/Y+ |  | - |  | 5 |  | Y-/Y+ |  | 0.79557 |  | 0.371 | |  | 2.1463 |  | 266 | |  | | 0.033 |  | |
| 1 |  | B- |  | - |  | 2 |  | B- |  | -0.01637 |  | 0.371 | |  | -0.0442 |  | 266 | |  | | 0.965 |  | |
| 1 |  | B- |  | - |  | 2 |  | X+/X- |  | -1.00335 |  | 0.371 | |  | -2.7068 |  | 266 | |  | | 0.007 |  | |
| 1 |  | B- |  | - |  | 2 |  | Y-/Y+ |  | -0.31325 |  | 0.371 | |  | -0.8451 |  | 266 | |  | | 0.399 |  | |
| 1 |  | B- |  | - |  | 1 |  | X+/X- |  | -0.29529 |  | 0.371 | |  | -0.7966 |  | 266 | |  | | 0.426 |  | |
| 1 |  | B- |  | - |  | 1 |  | Y-/Y+ |  | -0.14766 |  | 0.371 | |  | -0.3983 |  | 266 | |  | | 0.691 |  | |
| 1 |  | B- |  | - |  | 3 |  | B- |  | 0.09691 |  | 0.371 | |  | 0.2614 |  | 266 | |  | | 0.794 |  | |
| 1 |  | B- |  | - |  | 3 |  | X+/X- |  | -0.95257 |  | 0.371 | |  | -2.5698 |  | 266 | |  | | 0.011 |  | |
| 1 |  | B- |  | - |  | 3 |  | Y-/Y+ |  | 0.33649 |  | 0.371 | |  | 0.9078 |  | 266 | |  | | 0.365 |  | |
| 1 |  | B- |  | - |  | 4 |  | B- |  | 0.37685 |  | 0.371 | |  | 1.0167 |  | 266 | |  | | 0.310 |  | |
| 1 |  | B- |  | - |  | 4 |  | X+/X- |  | -0.87835 |  | 0.371 | |  | -2.3696 |  | 266 | |  | | 0.019 |  | |
| 1 |  | B- |  | - |  | 4 |  | Y-/Y+ |  | 0.25446 |  | 0.371 | |  | 0.6865 |  | 266 | |  | | 0.493 |  | |
| 1 |  | B- |  | - |  | 5 |  | B- |  | 0.40680 |  | 0.371 | |  | 1.0975 |  | 266 | |  | | 0.273 |  | |
| 1 |  | B- |  | - |  | 5 |  | X+/X- |  | -0.60882 |  | 0.371 | |  | -1.6425 |  | 266 | |  | | 0.102 |  | |
| 1 |  | B- |  | - |  | 5 |  | Y-/Y+ |  | 0.48232 |  | 0.371 | |  | 1.3012 |  | 266 | |  | | 0.194 |  | |
| 1 |  | A+ |  | - |  | 2 |  | B- |  | -0.07304 |  | 0.371 | |  | -0.1970 |  | 266 | |  | | 0.844 |  | |
| 1 |  | A+ |  | - |  | 2 |  | A+ |  | -1.06653 |  | 0.371 | |  | -2.8773 |  | 266 | |  | | 0.004 |  | |
| 1 |  | A+ |  | - |  | 2 |  | X+/X- |  | -1.06002 |  | 0.371 | |  | -2.8597 |  | 266 | |  | | 0.005 |  | |
| 1 |  | A+ |  | - |  | 2 |  | Y-/Y+ |  | -0.36992 |  | 0.371 | |  | -0.9980 |  | 266 | |  | | 0.319 |  | |
| 1 |  | A+ |  | - |  | 1 |  | B- |  | -0.05667 |  | 0.371 | |  | -0.1529 |  | 266 | |  | | 0.879 |  | |
| 1 |  | A+ |  | - |  | 1 |  | X+/X- |  | -0.35196 |  | 0.371 | |  | -0.9495 |  | 266 | |  | | 0.343 |  | |
| 1 |  | A+ |  | - |  | 1 |  | Y-/Y+ |  | -0.20432 |  | 0.371 | |  | -0.5512 |  | 266 | |  | | 0.582 |  | |
| 1 |  | A+ |  | - |  | 3 |  | B- |  | 0.04024 |  | 0.371 | |  | 0.1086 |  | 266 | |  | | 0.914 |  | |
| 1 |  | A+ |  | - |  | 3 |  | A+ |  | -1.45585 |  | 0.371 | |  | -3.9276 |  | 266 | |  | | < .001 |  | |
| 1 |  | A+ |  | - |  | 3 |  | X+/X- |  | -1.00924 |  | 0.371 | |  | -2.7227 |  | 266 | |  | | 0.007 |  | |
| 1 |  | A+ |  | - |  | 3 |  | Y-/Y+ |  | 0.27982 |  | 0.371 | |  | 0.7549 |  | 266 | |  | | 0.451 |  | |
| 1 |  | A+ |  | - |  | 4 |  | B- |  | 0.32019 |  | 0.371 | |  | 0.8638 |  | 266 | |  | | 0.388 |  | |
| 1 |  | A+ |  | - |  | 4 |  | A+ |  | -1.39986 |  | 0.371 | |  | -3.7765 |  | 266 | |  | | < .001 |  | |
| 1 |  | A+ |  | - |  | 4 |  | X+/X- |  | -0.93502 |  | 0.371 | |  | -2.5225 |  | 266 | |  | | 0.012 |  | |
| 1 |  | A+ |  | - |  | 4 |  | Y-/Y+ |  | 0.19779 |  | 0.371 | |  | 0.5336 |  | 266 | |  | | 0.594 |  | |
| 1 |  | A+ |  | - |  | 5 |  | B- |  | 0.35014 |  | 0.371 | |  | 0.9446 |  | 266 | |  | | 0.346 |  | |
| 1 |  | A+ |  | - |  | 5 |  | A+ |  | -1.05091 |  | 0.371 | |  | -2.8351 |  | 266 | |  | | 0.005 |  | |
| 1 |  | A+ |  | - |  | 5 |  | X+/X- |  | -0.66549 |  | 0.371 | |  | -1.7954 |  | 266 | |  | | 0.074 |  | |
| 1 |  | A+ |  | - |  | 5 |  | Y-/Y+ |  | 0.42566 |  | 0.371 | |  | 1.1483 |  | 266 | |  | | 0.252 |  | |
| 1 |  | X+/X- |  | - |  | 2 |  | X+/X- |  | -0.70806 |  | 0.371 | |  | -1.9102 |  | 266 | |  | | 0.057 |  | |
| 1 |  | X+/X- |  | - |  | 2 |  | Y-/Y+ |  | -0.01796 |  | 0.371 | |  | -0.0484 |  | 266 | |  | | 0.961 |  | |
| 1 |  | X+/X- |  | - |  | 1 |  | Y-/Y+ |  | 0.14764 |  | 0.371 | |  | 0.3983 |  | 266 | |  | | 0.691 |  | |
| 1 |  | X+/X- |  | - |  | 3 |  | X+/X- |  | -0.65728 |  | 0.371 | |  | -1.7732 |  | 266 | |  | | 0.077 |  | |
| 1 |  | X+/X- |  | - |  | 3 |  | Y-/Y+ |  | 0.63178 |  | 0.371 | |  | 1.7044 |  | 266 | |  | | 0.089 |  | |
| 1 |  | X+/X- |  | - |  | 4 |  | X+/X- |  | -0.58306 |  | 0.371 | |  | -1.5730 |  | 266 | |  | | 0.117 |  | |
| 1 |  | X+/X- |  | - |  | 4 |  | Y-/Y+ |  | 0.54975 |  | 0.371 | |  | 1.4831 |  | 266 | |  | | 0.139 |  | |
| 1 |  | X+/X- |  | - |  | 5 |  | X+/X- |  | -0.31353 |  | 0.371 | |  | -0.8458 |  | 266 | |  | | 0.398 |  | |
| 1 |  | X+/X- |  | - |  | 5 |  | Y-/Y+ |  | 0.77762 |  | 0.371 | |  | 2.0979 |  | 266 | |  | | 0.037 |  | |
| 1 |  | Y-/Y+ |  | - |  | 2 |  | Y-/Y+ |  | -0.16559 |  | 0.371 | |  | -0.4467 |  | 266 | |  | | 0.655 |  | |
| 1 |  | Y-/Y+ |  | - |  | 3 |  | Y-/Y+ |  | 0.48415 |  | 0.371 | |  | 1.3061 |  | 266 | |  | | 0.193 |  | |
| 1 |  | Y-/Y+ |  | - |  | 4 |  | Y-/Y+ |  | 0.40211 |  | 0.371 | |  | 1.0848 |  | 266 | |  | | 0.279 |  | |
| 1 |  | Y-/Y+ |  | - |  | 5 |  | Y-/Y+ |  | 0.62998 |  | 0.371 | |  | 1.6996 |  | 266 | |  | | 0.090 |  | |
| 3 |  | B- |  | - |  | 2 |  | X+/X- |  | -1.10026 |  | 0.371 | |  | -2.9683 |  | 266 | |  | | 0.003 |  | |
| 3 |  | B- |  | - |  | 2 |  | Y-/Y+ |  | -0.41016 |  | 0.371 | |  | -1.1065 |  | 266 | |  | | 0.270 |  | |
| 3 |  | B- |  | - |  | 1 |  | X+/X- |  | -0.39220 |  | 0.371 | |  | -1.0581 |  | 266 | |  | | 0.291 |  | |
| 3 |  | B- |  | - |  | 1 |  | Y-/Y+ |  | -0.24456 |  | 0.371 | |  | -0.6598 |  | 266 | |  | | 0.510 |  | |
| 3 |  | B- |  | - |  | 3 |  | X+/X- |  | -1.04948 |  | 0.371 | |  | -2.8313 |  | 266 | |  | | 0.005 |  | |
| 3 |  | B- |  | - |  | 3 |  | Y-/Y+ |  | 0.23958 |  | 0.371 | |  | 0.6463 |  | 266 | |  | | 0.519 |  | |
| 3 |  | B- |  | - |  | 4 |  | B- |  | 0.27995 |  | 0.371 | |  | 0.7552 |  | 266 | |  | | 0.451 |  | |
| 3 |  | B- |  | - |  | 4 |  | X+/X- |  | -0.97526 |  | 0.371 | |  | -2.6311 |  | 266 | |  | | 0.009 |  | |
| 3 |  | B- |  | - |  | 4 |  | Y-/Y+ |  | 0.15755 |  | 0.371 | |  | 0.4250 |  | 266 | |  | | 0.671 |  | |
| 3 |  | B- |  | - |  | 5 |  | B- |  | 0.30990 |  | 0.371 | |  | 0.8360 |  | 266 | |  | | 0.404 |  | |
| 3 |  | B- |  | - |  | 5 |  | X+/X- |  | -0.70573 |  | 0.371 | |  | -1.9039 |  | 266 | |  | | 0.058 |  | |
| 3 |  | B- |  | - |  | 5 |  | Y-/Y+ |  | 0.38542 |  | 0.371 | |  | 1.0398 |  | 266 | |  | | 0.299 |  | |
| 3 |  | A+ |  | - |  | 2 |  | B- |  | 1.38281 |  | 0.371 | |  | 3.7305 |  | 266 | |  | | < .001 |  | |
| 3 |  | A+ |  | - |  | 2 |  | X+/X- |  | 0.39583 |  | 0.371 | |  | 1.0679 |  | 266 | |  | | 0.287 |  | |
| 3 |  | A+ |  | - |  | 2 |  | Y-/Y+ |  | 1.08594 |  | 0.371 | |  | 2.9296 |  | 266 | |  | | 0.004 |  | |
| 3 |  | A+ |  | - |  | 1 |  | B- |  | 1.39919 |  | 0.371 | |  | 3.7747 |  | 266 | |  | | < .001 |  | |
| 3 |  | A+ |  | - |  | 1 |  | X+/X- |  | 1.10389 |  | 0.371 | |  | 2.9781 |  | 266 | |  | | 0.003 |  | |
| 3 |  | A+ |  | - |  | 1 |  | Y-/Y+ |  | 1.25153 |  | 0.371 | |  | 3.3764 |  | 266 | |  | | < .001 |  | |
| 3 |  | A+ |  | - |  | 3 |  | B- |  | 1.49609 |  | 0.371 | |  | 4.0362 |  | 266 | |  | | < .001 |  | |
| 3 |  | A+ |  | - |  | 3 |  | X+/X- |  | 0.44661 |  | 0.371 | |  | 1.2049 |  | 266 | |  | | 0.229 |  | |
| 3 |  | A+ |  | - |  | 3 |  | Y-/Y+ |  | 1.73568 |  | 0.371 | |  | 4.6825 |  | 266 | |  | | < .001 |  | |
| 3 |  | A+ |  | - |  | 4 |  | B- |  | 1.77604 |  | 0.371 | |  | 4.7914 |  | 266 | |  | | < .001 |  | |
| 3 |  | A+ |  | - |  | 4 |  | A+ |  | 0.05599 |  | 0.371 | |  | 0.1510 |  | 266 | |  | | 0.880 |  | |
| 3 |  | A+ |  | - |  | 4 |  | X+/X- |  | 0.52083 |  | 0.371 | |  | 1.4051 |  | 266 | |  | | 0.161 |  | |
| 3 |  | A+ |  | - |  | 4 |  | Y-/Y+ |  | 1.65365 |  | 0.371 | |  | 4.4612 |  | 266 | |  | | < .001 |  | |
| 3 |  | A+ |  | - |  | 5 |  | B- |  | 1.80599 |  | 0.371 | |  | 4.8722 |  | 266 | |  | | < .001 |  | |
| 3 |  | A+ |  | - |  | 5 |  | A+ |  | 0.40495 |  | 0.371 | |  | 1.0925 |  | 266 | |  | | 0.276 |  | |
| 3 |  | A+ |  | - |  | 5 |  | X+/X- |  | 0.79036 |  | 0.371 | |  | 2.1322 |  | 266 | |  | | 0.034 |  | |
| 3 |  | A+ |  | - |  | 5 |  | Y-/Y+ |  | 1.88151 |  | 0.371 | |  | 5.0759 |  | 266 | |  | | < .001 |  | |
| 3 |  | X+/X- |  | - |  | 2 |  | Y-/Y+ |  | 0.63932 |  | 0.371 | |  | 1.7248 |  | 266 | |  | | 0.086 |  | |
| 3 |  | X+/X- |  | - |  | 1 |  | Y-/Y+ |  | 0.80492 |  | 0.371 | |  | 2.1715 |  | 266 | |  | | 0.031 |  | |
| 3 |  | X+/X- |  | - |  | 3 |  | Y-/Y+ |  | 1.28906 |  | 0.371 | |  | 3.4776 |  | 266 | |  | | < .001 |  | |
| 3 |  | X+/X- |  | - |  | 4 |  | X+/X- |  | 0.07422 |  | 0.371 | |  | 0.2002 |  | 266 | |  | | 0.841 |  | |
| 3 |  | X+/X- |  | - |  | 4 |  | Y-/Y+ |  | 1.20703 |  | 0.371 | |  | 3.2563 |  | 266 | |  | | 0.001 |  | |
| 3 |  | X+/X- |  | - |  | 5 |  | X+/X- |  | 0.34375 |  | 0.371 | |  | 0.9274 |  | 266 | |  | | 0.355 |  | |
| 3 |  | X+/X- |  | - |  | 5 |  | Y-/Y+ |  | 1.43490 |  | 0.371 | |  | 3.8711 |  | 266 | |  | | < .001 |  | |
| 3 |  | Y-/Y+ |  | - |  | 4 |  | Y-/Y+ |  | -0.08203 |  | 0.371 | |  | -0.2213 |  | 266 | |  | | 0.825 |  | |
| 3 |  | Y-/Y+ |  | - |  | 5 |  | Y-/Y+ |  | 0.14583 |  | 0.371 | |  | 0.3934 |  | 266 | |  | | 0.694 |  | |
| 4 |  | B- |  | - |  | 2 |  | X+/X- |  | -1.38021 |  | 0.371 | |  | -3.7235 |  | 266 | |  | | < .001 |  | |
| 4 |  | B- |  | - |  | 2 |  | Y-/Y+ |  | -0.69010 |  | 0.371 | |  | -1.8618 |  | 266 | |  | | 0.064 |  | |
| 4 |  | B- |  | - |  | 1 |  | X+/X- |  | -0.67215 |  | 0.371 | |  | -1.8133 |  | 266 | |  | | 0.071 |  | |
| 4 |  | B- |  | - |  | 1 |  | Y-/Y+ |  | -0.52451 |  | 0.371 | |  | -1.4150 |  | 266 | |  | | 0.158 |  | |
| 4 |  | B- |  | - |  | 3 |  | X+/X- |  | -1.32943 |  | 0.371 | |  | -3.5865 |  | 266 | |  | | < .001 |  | |
| 4 |  | B- |  | - |  | 3 |  | Y-/Y+ |  | -0.04036 |  | 0.371 | |  | -0.1089 |  | 266 | |  | | 0.913 |  | |
| 4 |  | B- |  | - |  | 4 |  | X+/X- |  | -1.25521 |  | 0.371 | |  | -3.3863 |  | 266 | |  | | < .001 |  | |
| 4 |  | B- |  | - |  | 4 |  | Y-/Y+ |  | -0.12240 |  | 0.371 | |  | -0.3302 |  | 266 | |  | | 0.742 |  | |
| 4 |  | B- |  | - |  | 5 |  | B- |  | 0.02995 |  | 0.371 | |  | 0.0808 |  | 266 | |  | | 0.936 |  | |
| 4 |  | B- |  | - |  | 5 |  | X+/X- |  | -0.98568 |  | 0.371 | |  | -2.6592 |  | 266 | |  | | 0.008 |  | |
| 4 |  | B- |  | - |  | 5 |  | Y-/Y+ |  | 0.10547 |  | 0.371 | |  | 0.2845 |  | 266 | |  | | 0.776 |  | |
| 4 |  | A+ |  | - |  | 2 |  | B- |  | 1.32682 |  | 0.371 | |  | 3.5795 |  | 266 | |  | | < .001 |  | |
| 4 |  | A+ |  | - |  | 2 |  | X+/X- |  | 0.33984 |  | 0.371 | |  | 0.9168 |  | 266 | |  | | 0.360 |  | |
| 4 |  | A+ |  | - |  | 2 |  | Y-/Y+ |  | 1.02995 |  | 0.371 | |  | 2.7786 |  | 266 | |  | | 0.006 |  | |
| 4 |  | A+ |  | - |  | 1 |  | B- |  | 1.34320 |  | 0.371 | |  | 3.6237 |  | 266 | |  | | < .001 |  | |
| 4 |  | A+ |  | - |  | 1 |  | X+/X- |  | 1.04790 |  | 0.371 | |  | 2.8270 |  | 266 | |  | | 0.005 |  | |
| 4 |  | A+ |  | - |  | 1 |  | Y-/Y+ |  | 1.19554 |  | 0.371 | |  | 3.2253 |  | 266 | |  | | 0.001 |  | |
| 4 |  | A+ |  | - |  | 3 |  | B- |  | 1.44010 |  | 0.371 | |  | 3.8851 |  | 266 | |  | | < .001 |  | |
| 4 |  | A+ |  | - |  | 3 |  | X+/X- |  | 0.39062 |  | 0.371 | |  | 1.0538 |  | 266 | |  | | 0.293 |  | |
| 4 |  | A+ |  | - |  | 3 |  | Y-/Y+ |  | 1.67969 |  | 0.371 | |  | 4.5315 |  | 266 | |  | | < .001 |  | |
| 4 |  | A+ |  | - |  | 4 |  | B- |  | 1.72005 |  | 0.371 | |  | 4.6404 |  | 266 | |  | | < .001 |  | |
| 4 |  | A+ |  | - |  | 4 |  | X+/X- |  | 0.46484 |  | 0.371 | |  | 1.2541 |  | 266 | |  | | 0.211 |  | |
| 4 |  | A+ |  | - |  | 4 |  | Y-/Y+ |  | 1.59766 |  | 0.371 | |  | 4.3102 |  | 266 | |  | | < .001 |  | |
| 4 |  | A+ |  | - |  | 5 |  | B- |  | 1.75000 |  | 0.371 | |  | 4.7211 |  | 266 | |  | | < .001 |  | |
| 4 |  | A+ |  | - |  | 5 |  | A+ |  | 0.34896 |  | 0.371 | |  | 0.9414 |  | 266 | |  | | 0.347 |  | |
| 4 |  | A+ |  | - |  | 5 |  | X+/X- |  | 0.73438 |  | 0.371 | |  | 1.9812 |  | 266 | |  | | 0.049 |  | |
| 4 |  | A+ |  | - |  | 5 |  | Y-/Y+ |  | 1.82552 |  | 0.371 | |  | 4.9249 |  | 266 | |  | | < .001 |  | |
| 4 |  | X+/X- |  | - |  | 2 |  | Y-/Y+ |  | 0.56510 |  | 0.371 | |  | 1.5245 |  | 266 | |  | | 0.129 |  | |
| 4 |  | X+/X- |  | - |  | 1 |  | Y-/Y+ |  | 0.73070 |  | 0.371 | |  | 1.9713 |  | 266 | |  | | 0.050 |  | |
| 4 |  | X+/X- |  | - |  | 3 |  | Y-/Y+ |  | 1.21484 |  | 0.371 | |  | 3.2774 |  | 266 | |  | | 0.001 |  | |
| 4 |  | X+/X- |  | - |  | 4 |  | Y-/Y+ |  | 1.13281 |  | 0.371 | |  | 3.0561 |  | 266 | |  | | 0.002 |  | |
| 4 |  | X+/X- |  | - |  | 5 |  | X+/X- |  | 0.26953 |  | 0.371 | |  | 0.7271 |  | 266 | |  | | 0.468 |  | |
| 4 |  | X+/X- |  | - |  | 5 |  | Y-/Y+ |  | 1.36068 |  | 0.371 | |  | 3.6708 |  | 266 | |  | | < .001 |  | |
| 4 |  | Y-/Y+ |  | - |  | 5 |  | Y-/Y+ |  | 0.22786 |  | 0.371 | |  | 0.6147 |  | 266 | |  | | 0.539 |  | |
| 5 |  | B- |  | - |  | 2 |  | X+/X- |  | -1.41016 |  | 0.371 | |  | -3.8043 |  | 266 | |  | | < .001 |  | |
| 5 |  | B- |  | - |  | 2 |  | Y-/Y+ |  | -0.72005 |  | 0.371 | |  | -1.9426 |  | 266 | |  | | 0.053 |  | |
| 5 |  | B- |  | - |  | 1 |  | X+/X- |  | -0.70210 |  | 0.371 | |  | -1.8941 |  | 266 | |  | | 0.059 |  | |
| 5 |  | B- |  | - |  | 1 |  | Y-/Y+ |  | -0.55446 |  | 0.371 | |  | -1.4958 |  | 266 | |  | | 0.136 |  | |
| 5 |  | B- |  | - |  | 3 |  | X+/X- |  | -1.35938 |  | 0.371 | |  | -3.6673 |  | 266 | |  | | < .001 |  | |
| 5 |  | B- |  | - |  | 3 |  | Y-/Y+ |  | -0.07031 |  | 0.371 | |  | -0.1897 |  | 266 | |  | | 0.850 |  | |
| 5 |  | B- |  | - |  | 4 |  | X+/X- |  | -1.28516 |  | 0.371 | |  | -3.4671 |  | 266 | |  | | < .001 |  | |
| 5 |  | B- |  | - |  | 4 |  | Y-/Y+ |  | -0.15234 |  | 0.371 | |  | -0.4110 |  | 266 | |  | | 0.681 |  | |
| 5 |  | B- |  | - |  | 5 |  | X+/X- |  | -1.01562 |  | 0.371 | |  | -2.7400 |  | 266 | |  | | 0.007 |  | |
| 5 |  | B- |  | - |  | 5 |  | Y-/Y+ |  | 0.07552 |  | 0.371 | |  | 0.2037 |  | 266 | |  | | 0.839 |  | |
| 5 |  | A+ |  | - |  | 2 |  | B- |  | 0.97786 |  | 0.371 | |  | 2.6381 |  | 266 | |  | | 0.009 |  | |
| 5 |  | A+ |  | - |  | 2 |  | X+/X- |  | -0.00911 |  | 0.371 | |  | -0.0246 |  | 266 | |  | | 0.980 |  | |
| 5 |  | A+ |  | - |  | 2 |  | Y-/Y+ |  | 0.68099 |  | 0.371 | |  | 1.8372 |  | 266 | |  | | 0.067 |  | |
| 5 |  | A+ |  | - |  | 1 |  | B- |  | 0.99424 |  | 0.371 | |  | 2.6823 |  | 266 | |  | | 0.008 |  | |
| 5 |  | A+ |  | - |  | 1 |  | X+/X- |  | 0.69894 |  | 0.371 | |  | 1.8856 |  | 266 | |  | | 0.060 |  | |
| 5 |  | A+ |  | - |  | 1 |  | Y-/Y+ |  | 0.84658 |  | 0.371 | |  | 2.2839 |  | 266 | |  | | 0.023 |  | |
| 5 |  | A+ |  | - |  | 3 |  | B- |  | 1.09115 |  | 0.371 | |  | 2.9437 |  | 266 | |  | | 0.004 |  | |
| 5 |  | A+ |  | - |  | 3 |  | X+/X- |  | 0.04167 |  | 0.371 | |  | 0.1124 |  | 266 | |  | | 0.911 |  | |
| 5 |  | A+ |  | - |  | 3 |  | Y-/Y+ |  | 1.33073 |  | 0.371 | |  | 3.5900 |  | 266 | |  | | < .001 |  | |
| 5 |  | A+ |  | - |  | 4 |  | B- |  | 1.37109 |  | 0.371 | |  | 3.6989 |  | 266 | |  | | < .001 |  | |
| 5 |  | A+ |  | - |  | 4 |  | X+/X- |  | 0.11589 |  | 0.371 | |  | 0.3126 |  | 266 | |  | | 0.755 |  | |
| 5 |  | A+ |  | - |  | 4 |  | Y-/Y+ |  | 1.24870 |  | 0.371 | |  | 3.3687 |  | 266 | |  | | < .001 |  | |
| 5 |  | A+ |  | - |  | 5 |  | B- |  | 1.40104 |  | 0.371 | |  | 3.7797 |  | 266 | |  | | < .001 |  | |
| 5 |  | A+ |  | - |  | 5 |  | X+/X- |  | 0.38542 |  | 0.371 | |  | 1.0398 |  | 266 | |  | | 0.299 |  | |
| 5 |  | A+ |  | - |  | 5 |  | Y-/Y+ |  | 1.47656 |  | 0.371 | |  | 3.9835 |  | 266 | |  | | < .001 |  | |
| 5 |  | X+/X- |  | - |  | 2 |  | Y-/Y+ |  | 0.29557 |  | 0.371 | |  | 0.7974 |  | 266 | |  | | 0.426 |  | |
| 5 |  | X+/X- |  | - |  | 1 |  | Y-/Y+ |  | 0.46117 |  | 0.371 | |  | 1.2441 |  | 266 | |  | | 0.215 |  | |
| 5 |  | X+/X- |  | - |  | 3 |  | Y-/Y+ |  | 0.94531 |  | 0.371 | |  | 2.5503 |  | 266 | |  | | 0.011 |  | |
| 5 |  | X+/X- |  | - |  | 4 |  | Y-/Y+ |  | 0.86328 |  | 0.371 | |  | 2.3290 |  | 266 | |  | | 0.021 |  | |
| 5 |  | X+/X- |  | - |  | 5 |  | Y-/Y+ |  | 1.09115 |  | 0.371 | |  | 2.9437 |  | 266 | |  | | 0.004 |  | |
|  | | | | | | | | | | | | | | | | | | | | | | |  |

| Post Hoc Comparisons - Stimulus | | | | | | | | | | | | | | | |
| --- | --- | --- | --- | --- | --- | --- | --- | --- | --- | --- | --- | --- | --- | --- | --- |
| **Comparison** | | | | | |  | | | | | | | | | |
| **Stimulus** | |  | | **Stimulus** | | **Difference** | | **SE** | | **t** | | **df** | | **p** | |
| B- |  | - |  | X+/X- |  | -0.9205 |  | 0.166 |  | -5.553 |  | 266 |  | < .001 |  |
| B- |  | - |  | Y-/Y+ |  | -0.0504 |  | 0.166 |  | -0.304 |  | 266 |  | 0.762 |  |
| A+ |  | - |  | B- |  | 1.1108 |  | 0.166 |  | 6.701 |  | 266 |  | < .001 |  |
| A+ |  | - |  | X+/X- |  | 0.1903 |  | 0.166 |  | 1.148 |  | 266 |  | 0.252 |  |
| A+ |  | - |  | Y-/Y+ |  | 1.0604 |  | 0.166 |  | 6.397 |  | 266 |  | < .001 |  |
| X+/X- |  | - |  | Y-/Y+ |  | 0.8702 |  | 0.166 |  | 5.249 |  | 266 |  | < .001 |  |
|  | | | | | | | | | | | | | | | |

**Compound phase (target trials X and Y)**

| Fixed Effect Omnibus tests | | | | | | | | | |
| --- | --- | --- | --- | --- | --- | --- | --- | --- | --- |
|  |  |  |  |  |  |  |  |  |  |
|  | | **F** | | **Num df** | | **Den df** | | **p** | |
| Group |  | 0.5726 |  | 1 |  | 14.0 |  | 0.462 |  |
| 2Session Blocks |  | 1.7935 |  | 9 |  | 266.0 |  | 0.070 |  |
| Stimulus |  | 0.0457 |  | 1 |  | 266.0 |  | 0.831 |  |
| Group ✻ 2Session Blocks |  | 0.7549 |  | 9 |  | 266.0 |  | 0.658 |  |
| Group ✻ Stimulus |  | 14.6942 |  | 1 |  | 266.0 |  | < .001 |  |
| 2Session Blocks ✻ Stimulus |  | 2.1879 |  | 9 |  | 266.0 |  | 0.023 |  |
| Group ✻ 2Session Blocks ✻ Stimulus |  | 2.1646 |  | 9 |  | 266.0 |  | 0.025 |  |
|  | | | | | | | | | |

**Simple Effects**

| Simple effects of Stimulus : Parameter estimates | | | | | | | | | | | | | | | | | | | |  |
| --- | --- | --- | --- | --- | --- | --- | --- | --- | --- | --- | --- | --- | --- | --- | --- | --- | --- | --- | --- | --- |
| **Moderator levels** | | | |  | | | | | | **95% Confidence Interval** | | | |  | | | | | |  |
| **2Session Blocks** | | **Group** | | **contrast** | | **Estimate** | | **SE** | | **Lower** | | **Upper** | | **df** | | **t** | **p** | | |  |
| 1 |  | 1 |  | Y+ - X- |  | -0.6000 |  | 0.440 |  | -1.4670 |  | 0.26698 |  | 266 |  | -1.3626 |  | 0.174 |  | |
|  |  | 2 |  | Y+ - X- |  | -0.7410 |  | 0.440 |  | -1.6079 |  | 0.12600 |  | 266 |  | -1.6828 |  | 0.094 |  | |
| 2 |  | 1 |  | Y+ - X- |  | -0.5944 |  | 0.440 |  | -1.4614 |  | 0.27253 |  | 266 |  | -1.3500 |  | 0.178 |  | |
|  |  | 2 |  | Y+ - X- |  | -0.5153 |  | 0.440 |  | -1.3823 |  | 0.35170 |  | 266 |  | -1.1702 |  | 0.243 |  | |
| 3 |  | 1 |  | Y+ - X- |  | -0.5285 |  | 0.440 |  | -1.3954 |  | 0.33850 |  | 266 |  | -1.2002 |  | 0.231 |  | |
|  |  | 2 |  | Y+ - X- |  | -0.5542 |  | 0.440 |  | -1.4211 |  | 0.31281 |  | 266 |  | -1.2585 |  | 0.209 |  | |
| 4 |  | 1 |  | Y+ - X- |  | -0.0958 |  | 0.440 |  | -0.9628 |  | 0.77114 |  | 266 |  | -0.2176 |  | 0.828 |  | |
|  |  | 2 |  | Y+ - X- |  | -0.0299 |  | 0.440 |  | -0.8968 |  | 0.83712 |  | 266 |  | -0.0678 |  | 0.946 |  | |
| 5 |  | 1 |  | Y+ - X- |  | 0.1319 |  | 0.440 |  | -0.7350 |  | 0.99892 |  | 266 |  | 0.2996 |  | 0.765 |  | |
|  |  | 2 |  | Y+ - X- |  | 0.0424 |  | 0.440 |  | -0.8246 |  | 0.90934 |  | 266 |  | 0.0962 |  | 0.923 |  | |
| 6 |  | 1 |  | Y+ - X- |  | 0.7111 |  | 0.440 |  | -0.1559 |  | 1.57809 |  | 266 |  | 1.6149 |  | 0.108 |  | |
|  |  | 2 |  | Y+ - X- |  | -0.0701 |  | 0.440 |  | -0.9371 |  | 0.79684 |  | 266 |  | -0.1593 |  | 0.874 |  | |
| 7 |  | 1 |  | Y+ - X- |  | 0.8410 |  | 0.440 |  | -0.0260 |  | 1.70795 |  | 266 |  | 1.9099 |  | 0.057 |  | |
|  |  | 2 |  | Y+ - X- |  | -0.2278 |  | 0.440 |  | -1.0948 |  | 0.63920 |  | 266 |  | -0.5173 |  | 0.605 |  | |
| 8 |  | 1 |  | Y+ - X- |  | 0.8312 |  | 0.440 |  | -0.0357 |  | 1.69823 |  | 266 |  | 1.8878 |  | 0.060 |  | |
|  |  | 2 |  | Y+ - X- |  | -0.2375 |  | 0.440 |  | -1.1045 |  | 0.62948 |  | 266 |  | -0.5394 |  | 0.590 |  | |
| 9 |  | 1 |  | Y+ - X- |  | 1.6243 |  | 0.440 |  | 0.7573 |  | 2.49128 |  | 266 |  | 3.6888 |  | < .001 |  | |
|  |  | 2 |  | Y+ - X- |  | -0.8687 |  | 0.440 |  | -1.7357 |  | -0.00177 |  | 266 |  | -1.9730 |  | 0.050 |  | |
| 10 |  | 1 |  | Y+ - X- |  | 1.6639 |  | 0.440 |  | 0.7969 |  | 2.53087 |  | 266 |  | 3.7787 |  | < .001 |  | |
|  |  | 2 |  | Y+ - X- |  | -0.3618 |  | 0.440 |  | -1.2288 |  | 0.50517 |  | 266 |  | -0.8217 |  | 0.412 |  | |
|  | | | | | | | | | | | | | | | | | | | |  |

**Compound phase (excluding X and Y trials)**

| Fixed Effect Omnibus tests | | | | | | | | | |
| --- | --- | --- | --- | --- | --- | --- | --- | --- | --- |
|  |  |  |  |  |  |  |  |  |  |
|  | | **F** | | **Num df** | | **Den df** | | **p** | |
| Group |  | 0.0131 |  | 1 |  | 14.0 |  | 0.910 |  |
| 2 Session Block |  | 0.4040 |  | 9 |  | 546.0 |  | 0.933 |  |
| Stimulus |  | 138.8247 |  | 3 |  | 546.0 |  | < .001 |  |
| Group ✻ 2 Session Block |  | 0.4738 |  | 9 |  | 546.0 |  | 0.892 |  |
| Group ✻ Stimulus |  | 3.2285 |  | 3 |  | 546.0 |  | 0.022 |  |
| 2 Session Block ✻ Stimulus |  | 0.3968 |  | 27 |  | 546.0 |  | 0.998 |  |
| Group ✻ 2 Session Block ✻ Stimulus |  | 0.2982 |  | 27 |  | 546.0 |  | 1.000 |  |
| Note. Satterthwaite method for degrees of freedom | | | | | | | | | |
|  | | | | | | | | | |

| Post Hoc Comparisons - Group ✻ Stimulus | | | | | | | | | | | | | | | | | | | |
| --- | --- | --- | --- | --- | --- | --- | --- | --- | --- | --- | --- | --- | --- | --- | --- | --- | --- | --- | --- |
| **Comparison** | | | | | | | | | |  | | | | | | | | | |
| **Group** | | **Stimulus** | |  | | **Group** | | **Stimulus** | | **Difference** | | **SE** | | **t** | | **df** | | **p_bonferroni_** | |
| 2 |  | B- |  | - |  | 2 |  | BY- |  | -0.3226 |  | 0.189 |  | -1.7025 |  | 546.0 |  | 1.000 |  |
| 2 |  | B- |  | - |  | 1 |  | BY- |  | -0.2937 |  | 0.594 |  | -0.4943 |  | 16.4 |  | 1.000 |  |
| 2 |  | A+ |  | - |  | 2 |  | B- |  | 1.7548 |  | 0.189 |  | 9.2603 |  | 546.0 |  | < .001 |  |
| 2 |  | A+ |  | - |  | 2 |  | BY- |  | 1.4321 |  | 0.189 |  | 7.5578 |  | 546.0 |  | < .001 |  |
| 2 |  | A+ |  | - |  | 2 |  | AX+ |  | -0.7311 |  | 0.189 |  | -3.8580 |  | 546.0 |  | 0.004 |  |
| 2 |  | A+ |  | - |  | 1 |  | B- |  | 1.7544 |  | 0.594 |  | 2.9527 |  | 16.4 |  | 0.257 |  |
| 2 |  | A+ |  | - |  | 1 |  | BY- |  | 1.4611 |  | 0.594 |  | 2.4590 |  | 16.4 |  | 0.711 |  |
| 2 |  | A+ |  | - |  | 1 |  | AX+ |  | -0.2041 |  | 0.594 |  | -0.3434 |  | 16.4 |  | 1.000 |  |
| 2 |  | AX+ |  | - |  | 2 |  | B- |  | 2.4858 |  | 0.189 |  | 13.1183 |  | 546.0 |  | < .001 |  |
| 2 |  | AX+ |  | - |  | 2 |  | BY- |  | 2.1632 |  | 0.189 |  | 11.4158 |  | 546.0 |  | < .001 |  |
| 2 |  | AX+ |  | - |  | 1 |  | B- |  | 2.4855 |  | 0.594 |  | 4.1831 |  | 16.4 |  | 0.019 |  |
| 2 |  | AX+ |  | - |  | 1 |  | BY- |  | 2.1922 |  | 0.594 |  | 3.6894 |  | 16.4 |  | 0.054 |  |
| 1 |  | B- |  | - |  | 2 |  | B- |  | 3.65e-4 |  | 0.594 |  | 6.14e-4 |  | 16.4 |  | 1.000 |  |
| 1 |  | B- |  | - |  | 2 |  | BY- |  | -0.3222 |  | 0.594 |  | -0.5423 |  | 16.4 |  | 1.000 |  |
| 1 |  | B- |  | - |  | 1 |  | BY- |  | -0.2933 |  | 0.189 |  | -1.5478 |  | 546.0 |  | 1.000 |  |
| 1 |  | BY- |  | - |  | 2 |  | BY- |  | -0.0289 |  | 0.594 |  | -0.0487 |  | 16.4 |  | 1.000 |  |
| 1 |  | A+ |  | - |  | 2 |  | B- |  | 2.0486 |  | 0.594 |  | 3.4478 |  | 16.4 |  | 0.090 |  |
| 1 |  | A+ |  | - |  | 2 |  | BY- |  | 1.7260 |  | 0.594 |  | 2.9048 |  | 16.4 |  | 0.284 |  |
| 1 |  | A+ |  | - |  | 2 |  | A+ |  | 0.2938 |  | 0.594 |  | 0.4945 |  | 16.4 |  | 1.000 |  |
| 1 |  | A+ |  | - |  | 2 |  | AX+ |  | -0.4373 |  | 0.594 |  | -0.7359 |  | 16.4 |  | 1.000 |  |
| 1 |  | A+ |  | - |  | 1 |  | B- |  | 2.0482 |  | 0.189 |  | 10.8089 |  | 546.0 |  | < .001 |  |
| 1 |  | A+ |  | - |  | 1 |  | BY- |  | 1.7549 |  | 0.189 |  | 9.2611 |  | 546.0 |  | < .001 |  |
| 1 |  | A+ |  | - |  | 1 |  | AX+ |  | 0.0898 |  | 0.189 |  | 0.4737 |  | 546.0 |  | 1.000 |  |
| 1 |  | AX+ |  | - |  | 2 |  | B- |  | 1.9588 |  | 0.594 |  | 3.2967 |  | 16.4 |  | 0.124 |  |
| 1 |  | AX+ |  | - |  | 2 |  | BY- |  | 1.6362 |  | 0.594 |  | 2.7537 |  | 16.4 |  | 0.389 |  |
| 1 |  | AX+ |  | - |  | 2 |  | AX+ |  | -0.5270 |  | 0.594 |  | -0.8870 |  | 16.4 |  | 1.000 |  |
| 1 |  | AX+ |  | - |  | 1 |  | B- |  | 1.9584 |  | 0.189 |  | 10.3352 |  | 546.0 |  | < .001 |  |
| 1 |  | AX+ |  | - |  | 1 |  | BY- |  | 1.6651 |  | 0.189 |  | 8.7873 |  | 546.0 |  | < .001 |  |
|  | | | | | | | | | | | | | | | | | | | |

**Experiment 3: Agency rescues competitive credit assignment in the absence of an explicit configural solution**

**Pretraining phase**

| Fixed Effect Omnibus tests | | | | | | | | | |
| --- | --- | --- | --- | --- | --- | --- | --- | --- | --- |
|  |  |  |  |  |  |  |  |  |  |
|  | | **F** | | **Num df** | | **Den df** | | **p** | |
| group |  | 0.2352 |  | 1 |  | 14.0 |  | 0.635 |  |
| Stim |  | 88.7195 |  | 1 |  | 98.0 |  | < .001 |  |
| 2SeBlock |  | 1.4028 |  | 3 |  | 98.0 |  | 0.247 |  |
| group ✻ Stim |  | 2.8776 |  | 1 |  | 98.0 |  | 0.093 |  |
| group ✻ 2SeBlock |  | 0.1606 |  | 3 |  | 98.0 |  | 0.923 |  |
| Stim ✻ 2SeBlock |  | 5.3191 |  | 3 |  | 98.0 |  | 0.002 |  |
| group ✻ Stim ✻ 2SeBlock |  | 0.0274 |  | 3 |  | 98.0 |  | 0.994 |  |
| Note. Satterthwaite method for degrees of freedom | | | | | | | | | |

| Post Hoc Comparisons - Stim ✻ 2SeBlock | | | | | | | | | | | | | | | | | | | |
| --- | --- | --- | --- | --- | --- | --- | --- | --- | --- | --- | --- | --- | --- | --- | --- | --- | --- | --- | --- |
| **Comparison** | | | | | | | | | |  | | | | | | | | | |
| **Stim** | | **2SeBlock** | |  | | **Stim** | | **2SeBlock** | | **Difference** | | **SE** | | **t** | | **df** | | **p** | |
| B- |  | 2 |  | - |  | B- |  | 3 |  | 0.3108 |  | 0.445 |  | 0.699 |  | 98.0 |  | 0.486 |  |
| B- |  | 2 |  | - |  | B- |  | 4 |  | 0.3705 |  | 0.445 |  | 0.833 |  | 98.0 |  | 0.407 |  |
| B- |  | 2 |  | - |  | A+ |  | 3 |  | -2.3186 |  | 0.445 |  | -5.212 |  | 98.0 |  | < .001 |  |
| B- |  | 2 |  | - |  | A+ |  | 4 |  | -2.5468 |  | 0.445 |  | -5.725 |  | 98.0 |  | < .001 |  |
| B- |  | 1 |  | - |  | B- |  | 2 |  | 0.2383 |  | 0.445 |  | 0.536 |  | 98.0 |  | 0.593 |  |
| B- |  | 1 |  | - |  | B- |  | 3 |  | 0.5491 |  | 0.445 |  | 1.234 |  | 98.0 |  | 0.220 |  |
| B- |  | 1 |  | - |  | B- |  | 4 |  | 0.6088 |  | 0.445 |  | 1.369 |  | 98.0 |  | 0.174 |  |
| B- |  | 1 |  | - |  | A+ |  | 2 |  | -1.9765 |  | 0.445 |  | -4.443 |  | 98.0 |  | < .001 |  |
| B- |  | 1 |  | - |  | A+ |  | 3 |  | -2.0803 |  | 0.445 |  | -4.677 |  | 98.0 |  | < .001 |  |
| B- |  | 1 |  | - |  | A+ |  | 4 |  | -2.3085 |  | 0.445 |  | -5.190 |  | 98.0 |  | < .001 |  |
| B- |  | 3 |  | - |  | B- |  | 4 |  | 0.0597 |  | 0.445 |  | 0.134 |  | 98.0 |  | 0.893 |  |
| B- |  | 3 |  | - |  | A+ |  | 4 |  | -2.8576 |  | 0.445 |  | -6.424 |  | 98.0 |  | < .001 |  |
| A+ |  | 2 |  | - |  | B- |  | 2 |  | 2.2148 |  | 0.445 |  | 4.979 |  | 98.0 |  | < .001 |  |
| A+ |  | 2 |  | - |  | B- |  | 3 |  | 2.5256 |  | 0.445 |  | 5.678 |  | 98.0 |  | < .001 |  |
| A+ |  | 2 |  | - |  | B- |  | 4 |  | 2.5853 |  | 0.445 |  | 5.812 |  | 98.0 |  | < .001 |  |
| A+ |  | 2 |  | - |  | A+ |  | 3 |  | -0.1038 |  | 0.445 |  | -0.233 |  | 98.0 |  | 0.816 |  |
| A+ |  | 2 |  | - |  | A+ |  | 4 |  | -0.3320 |  | 0.445 |  | -0.746 |  | 98.0 |  | 0.457 |  |
| A+ |  | 1 |  | - |  | B- |  | 2 |  | 0.8564 |  | 0.445 |  | 1.925 |  | 98.0 |  | 0.057 |  |
| A+ |  | 1 |  | - |  | B- |  | 1 |  | 0.6181 |  | 0.445 |  | 1.390 |  | 98.0 |  | 0.168 |  |
| A+ |  | 1 |  | - |  | B- |  | 3 |  | 1.1672 |  | 0.445 |  | 2.624 |  | 98.0 |  | 0.010 |  |
| A+ |  | 1 |  | - |  | B- |  | 4 |  | 1.2269 |  | 0.445 |  | 2.758 |  | 98.0 |  | 0.007 |  |
| A+ |  | 1 |  | - |  | A+ |  | 2 |  | -1.3584 |  | 0.445 |  | -3.054 |  | 98.0 |  | 0.003 |  |
| A+ |  | 1 |  | - |  | A+ |  | 3 |  | -1.4622 |  | 0.445 |  | -3.287 |  | 98.0 |  | 0.001 |  |
| A+ |  | 1 |  | - |  | A+ |  | 4 |  | -1.6904 |  | 0.445 |  | -3.800 |  | 98.0 |  | < .001 |  |
| A+ |  | 3 |  | - |  | B- |  | 3 |  | 2.6294 |  | 0.445 |  | 5.911 |  | 98.0 |  | < .001 |  |
| A+ |  | 3 |  | - |  | B- |  | 4 |  | 2.6891 |  | 0.445 |  | 6.045 |  | 98.0 |  | < .001 |  |
| A+ |  | 3 |  | - |  | A+ |  | 4 |  | -0.2282 |  | 0.445 |  | -0.513 |  | 98.0 |  | 0.609 |  |
| A+ |  | 4 |  | - |  | B- |  | 4 |  | 2.9173 |  | 0.445 |  | 6.558 |  | 98.0 |  | < .001 |  |
|  | | | | | | | | | | | | | | | | | | | |

**Compound phase (excluding X and Y probe trials)**

| Fixed Effect Omnibus tests | | | | | | | | | |
| --- | --- | --- | --- | --- | --- | --- | --- | --- | --- |
|  |  |  |  |  |  |  |  |  |  |
|  | | **F** | | **Num df** | | **Den df** | | **p** | |
| group |  | 0.00198 |  | 1 |  | 14.0 |  | 0.965 |  |
| 4Session Block |  | 1.62014 |  | 7 |  | 434.0 |  | 0.128 |  |
| Stimulus |  | 142.32809 |  | 3 |  | 434.0 |  | < .001 |  |
| group ✻ 4Session Block |  | 0.63142 |  | 7 |  | 434.0 |  | 0.730 |  |
| group ✻ Stimulus |  | 7.87936 |  | 3 |  | 434.0 |  | < .001 |  |
| 4Session Block ✻ Stimulus |  | 0.89007 |  | 21 |  | 434.0 |  | 0.605 |  |
| group ✻ 4Session Block ✻ Stimulus |  | 0.20714 |  | 21 |  | 434.0 |  | 1.000 |  |
| Note. Satterthwaite method for degrees of freedom | | | | | | | | | |
|  | | | | | | | | | |

| Post Hoc Comparisons - group ✻ Stimulus | | | | | | | | | | | | | | | | | | | |
| --- | --- | --- | --- | --- | --- | --- | --- | --- | --- | --- | --- | --- | --- | --- | --- | --- | --- | --- | --- |
| **Comparison** | | | | | | | | | |  | | | | | | | | | |
| **group** | | **Stimulus** | |  | | **group** | | **Stimulus** | | **Difference** | | **SE** | | **t** | | **df** | | **p_bonferroni_** | |
| 2 |  | B- |  | - |  | 2 |  | BY+/BY- |  | -0.61177 |  | 0.185 |  | -3.3013 |  | 434.0 |  | 0.029 |  |
| 2 |  | B- |  | - |  | 1 |  | BY+/BY- |  | -1.36908 |  | 0.624 |  | -2.1928 |  | 16.0 |  | 1.000 |  |
| 2 |  | A+ |  | - |  | 2 |  | B- |  | 2.46173 |  | 0.185 |  | 13.2841 |  | 434.0 |  | < .001 |  |
| 2 |  | A+ |  | - |  | 2 |  | BY+/BY- |  | 1.84996 |  | 0.185 |  | 9.9828 |  | 434.0 |  | < .001 |  |
| 2 |  | A+ |  | - |  | 2 |  | AX+/AX- |  | 0.00332 |  | 0.185 |  | 0.0179 |  | 434.0 |  | 1.000 |  |
| 2 |  | A+ |  | - |  | 1 |  | B- |  | 2.42896 |  | 0.624 |  | 3.8904 |  | 16.0 |  | 0.036 |  |
| 2 |  | A+ |  | - |  | 1 |  | BY+/BY- |  | 1.09264 |  | 0.624 |  | 1.7501 |  | 16.0 |  | 1.000 |  |
| 2 |  | A+ |  | - |  | 1 |  | AX+/AX- |  | 0.28515 |  | 0.624 |  | 0.4567 |  | 16.0 |  | 1.000 |  |
| 2 |  | AX+/AX- |  | - |  | 2 |  | B- |  | 2.45840 |  | 0.185 |  | 13.2662 |  | 434.0 |  | < .001 |  |
| 2 |  | AX+/AX- |  | - |  | 2 |  | BY+/BY- |  | 1.84663 |  | 0.185 |  | 9.9649 |  | 434.0 |  | < .001 |  |
| 2 |  | AX+/AX- |  | - |  | 1 |  | B- |  | 2.42564 |  | 0.624 |  | 3.8851 |  | 16.0 |  | 0.037 |  |
| 2 |  | AX+/AX- |  | - |  | 1 |  | BY+/BY- |  | 1.08932 |  | 0.624 |  | 1.7448 |  | 16.0 |  | 1.000 |  |
| 1 |  | B- |  | - |  | 2 |  | B- |  | 0.03276 |  | 0.624 |  | 0.0525 |  | 16.0 |  | 1.000 |  |
| 1 |  | B- |  | - |  | 2 |  | BY+/BY- |  | -0.57901 |  | 0.624 |  | -0.9274 |  | 16.0 |  | 1.000 |  |
| 1 |  | B- |  | - |  | 1 |  | BY+/BY- |  | -1.33632 |  | 0.185 |  | -7.2111 |  | 434.0 |  | < .001 |  |
| 1 |  | BY+/BY- |  | - |  | 2 |  | BY+/BY- |  | 0.75731 |  | 0.624 |  | 1.2130 |  | 16.0 |  | 1.000 |  |
| 1 |  | A+ |  | - |  | 2 |  | B- |  | 2.06095 |  | 0.624 |  | 3.3010 |  | 16.0 |  | 0.126 |  |
| 1 |  | A+ |  | - |  | 2 |  | BY+/BY- |  | 1.44918 |  | 0.624 |  | 2.3211 |  | 16.0 |  | 0.945 |  |
| 1 |  | A+ |  | - |  | 2 |  | A+ |  | -0.40078 |  | 0.624 |  | -0.6419 |  | 16.0 |  | 1.000 |  |
| 1 |  | A+ |  | - |  | 2 |  | AX+/AX- |  | -0.39746 |  | 0.624 |  | -0.6366 |  | 16.0 |  | 1.000 |  |
| 1 |  | A+ |  | - |  | 1 |  | B- |  | 2.02818 |  | 0.185 |  | 10.9446 |  | 434.0 |  | < .001 |  |
| 1 |  | A+ |  | - |  | 1 |  | BY+/BY- |  | 0.69186 |  | 0.185 |  | 3.7335 |  | 434.0 |  | 0.006 |  |
| 1 |  | A+ |  | - |  | 1 |  | AX+/AX- |  | -0.11563 |  | 0.185 |  | -0.6240 |  | 434.0 |  | 1.000 |  |
| 1 |  | AX+/AX- |  | - |  | 2 |  | B- |  | 2.17658 |  | 0.624 |  | 3.4862 |  | 16.0 |  | 0.085 |  |
| 1 |  | AX+/AX- |  | - |  | 2 |  | BY+/BY- |  | 1.56481 |  | 0.624 |  | 2.5063 |  | 16.0 |  | 0.653 |  |
| 1 |  | AX+/AX- |  | - |  | 2 |  | AX+/AX- |  | -0.28183 |  | 0.624 |  | -0.4514 |  | 16.0 |  | 1.000 |  |
| 1 |  | AX+/AX- |  | - |  | 1 |  | B- |  | 2.14382 |  | 0.185 |  | 11.5686 |  | 434.0 |  | < .001 |  |
| 1 |  | AX+/AX- |  | - |  | 1 |  | BY+/BY- |  | 0.80749 |  | 0.185 |  | 4.3574 |  | 434.0 |  | < .001 |  |
|  | | | | | | | | | | | | | | | | | | | |

**Compound phase (probe trials X and Y)**

| Fixed Effect Omnibus tests | | | | | | | | | |
| --- | --- | --- | --- | --- | --- | --- | --- | --- | --- |
|  |  |  |  |  |  |  |  |  |  |
|  | | **F** | | **Num df** | | **Den df** | | **p** | |
| Group |  | 6.89e-4 |  | 1 |  | 14.0 |  | 0.979 |  |
| 4SessionBlock |  | 2.7234 |  | 4 |  | 126.0 |  | 0.032 |  |
| Stimulus |  | 3.8615 |  | 1 |  | 126.0 |  | 0.052 |  |
| Group ✻ 4SessionBlock |  | 0.3480 |  | 4 |  | 126.0 |  | 0.845 |  |
| Group ✻ Stimulus |  | 5.4289 |  | 1 |  | 126.0 |  | 0.021 |  |
| 4SessionBlock ✻ Stimulus |  | 0.6083 |  | 4 |  | 126.0 |  | 0.657 |  |
| Group ✻ 4SessionBlock ✻ Stimulus |  | 0.0549 |  | 4 |  | 126.0 |  | 0.994 |  |
| Note. Satterthwaite method for degrees of freedom | | | | | | | | | |
|  | | | | | | | | | |

**Simple Effects**

| Simple effects of Stimulus : Parameter estimates | | | | | | | | | | | | | | | | |  |  |
| --- | --- | --- | --- | --- | --- | --- | --- | --- | --- | --- | --- | --- | --- | --- | --- | --- | --- | --- |
| **Moderator levels** | |  | | | | | | **95% Confidence Interval** | | | |  | | | | | | |
| **Group** | | **contrast** | | **Estimate** | | **SE** | | **Lower** | | **Upper** | | **df** | | **t** | | **p** | | |
| 1 |  | Y- - X- |  | 0.4781 |  | 0.157 |  | 0.167 |  | 0.790 |  | 126 |  | 3.037 |  | 0.003 | |  |
| 2 |  | Y- - X- |  | -0.0406 |  | 0.157 |  | -0.352 |  | 0.271 |  | 126 |  | -0.258 |  | 0.797 | |  |
| Note. Simple effects are estimated keeping constant other independent variable(s) in the model | | | | | | | | | | | | | | | | | | |
|  | | | | | | | | | | | | | | | | | | |

**Experiment 4: Ruling out alternatives to the role of agency in competitive credit assignment**

**Negative patterning discrimination**

| Fixed Effect Omnibus tests | | | | | | | | | |
| --- | --- | --- | --- | --- | --- | --- | --- | --- | --- |
|  |  |  |  |  |  |  |  |  |  |
|  | | **F** | | **Num df** | | **Den df** | | **p** | |
| Group |  | 1.922 |  | 1 |  | 14.0 |  | 0.187 |  |
| 3SessionBlock |  | 1.272 |  | 13 |  | 378.0 |  | 0.227 |  |
| Stimulus |  | 91.600 |  | 1 |  | 378.0 |  | < .001 |  |
| Group ✻ 3SessionBlock |  | 0.928 |  | 13 |  | 378.0 |  | 0.524 |  |
| Group ✻ Stimulus |  | 33.477 |  | 1 |  | 378.0 |  | < .001 |  |
| 3SessionBlock ✻ Stimulus |  | 5.108 |  | 13 |  | 378.0 |  | < .001 |  |
| Group ✻ 3SessionBlock ✻ Stimulus |  | 1.058 |  | 13 |  | 378.0 |  | 0.395 |  |
| Note. Satterthwaite method for degrees of freedom | | | | | | | | | |
|  | | | | | | | | | |

## Simple Effects

| Simple effects of Stimulus : Parameter estimates | | | | | | | | | | | | | | | | | |
| --- | --- | --- | --- | --- | --- | --- | --- | --- | --- | --- | --- | --- | --- | --- | --- | --- | --- |
| **Moderator levels** | |  | | | | | | **95% Confidence Interval** | | | |  | | | | | |
| **Group** | | **contrast** | | **Estimate** | | **SE** | | **Lower** | | **Upper** | | **df** | | **t** | | **p** | |
| 1 |  | AX- - A/X+ |  | -0.376 |  | 0.140 |  | -0.652 |  | -0.0997 |  | 378 |  | -2.68 |  | 0.008 |  |
| 2 |  | AX- - A/X+ |  | -1.525 |  | 0.140 |  | -1.801 |  | -1.2488 |  | 378 |  | -10.86 |  | < .001 |  |
| Note. Simple effects are estimated keeping constant other independent variable(s) in the model | | | | | | | | | | | | | | | | | |
|  | | | | | | | | | | | | | | | | | |

**Positive patterning discrimination**

| Fixed Effect Omnibus tests | | | | | | | | | |
| --- | --- | --- | --- | --- | --- | --- | --- | --- | --- |
|  |  |  |  |  |  |  |  |  |  |
|  | | **F** | | **Num df** | | **Den df** | | **p** | |
| Group |  | 1.079 |  | 1 |  | 14.0 |  | 0.317 |  |
| 3SessionBlock |  | 0.834 |  | 13 |  | 378.0 |  | 0.624 |  |
| Stimulus |  | 106.558 |  | 1 |  | 378.0 |  | < .001 |  |
| Group ✻ 3SessionBlock |  | 1.038 |  | 13 |  | 378.0 |  | 0.413 |  |
| Group ✻ Stimulus |  | 0.105 |  | 1 |  | 378.0 |  | 0.747 |  |
| 3SessionBlock ✻ Stimulus |  | 1.328 |  | 13 |  | 378.0 |  | 0.194 |  |
| Group ✻ 3SessionBlock ✻ Stimulus |  | 0.351 |  | 13 |  | 378.0 |  | 0.983 |  |
| Note. Satterthwaite method for degrees of freedom | | | | | | | | | |
|  | | | | | | | | | |

**Exp. S1: Piloting a novel cue-competition task in a standard Pavlovian magazine-approach setting**

**Pretraining phase**

| Within Subjects Effects | | | | | | | | | | | |
| --- | --- | --- | --- | --- | --- | --- | --- | --- | --- | --- | --- |
|  |  |  |  |  |  |  |  |  |  |  |  |
|  | | **Sum of Squares** | | **df** | | **Mean Square** | | **F** | | **p** | |
| Stim |  | 88.6 |  | 3 |  | 29.529 |  | 34.50 |  | < .001 |  |
| Residual |  | 18.0 |  | 21 |  | 0.856 |  |  |  |  |  |
| block |  | 106.9 |  | 4 |  | 26.719 |  | 11.79 |  | < .001 |  |
| Residual |  | 63.4 |  | 28 |  | 2.266 |  |  |  |  |  |
| Stim ✻ block |  | 24.3 |  | 12 |  | 2.029 |  | 4.79 |  | < .001 |  |
| Residual |  | 35.6 |  | 84 |  | 0.423 |  |  |  |  |  |
| Note. Type 3 Sums of Squares | | | | | | | | | | | |
|  | | | | | | | | | | | |

| Post Hoc Comparisons - Stim ✻ block | | | | | | | | | | | | | | | | | | | |
| --- | --- | --- | --- | --- | --- | --- | --- | --- | --- | --- | --- | --- | --- | --- | --- | --- | --- | --- | --- |
| **Comparison** | | | | | | | | | |  | | | | | | | | | |
| **Stim** | | **block** | |  | | **Stim** | | **block** | | **Mean Difference** | | **SE** | | **df** | | **t** | | **p** | |
| a |  | 1 |  | - |  | a |  | 2 |  | -1.7852 |  | 0.3203 |  | 7.00 |  | -5.5725 |  | < .001 |  |
|  |  |  |  | - |  | a |  | 3 |  | -2.7656 |  | 0.5476 |  | 7.00 |  | -5.0507 |  | 0.001 |  |
|  |  |  |  | - |  | a |  | 4 |  | -3.4336 |  | 0.4960 |  | 7.00 |  | -6.9222 |  | < .001 |  |
|  |  |  |  | - |  | a |  | 5 |  | -3.1563 |  | 0.5866 |  | 7.00 |  | -5.3803 |  | 0.001 |  |
|  |  |  |  | - |  | b |  | 1 |  | 0.1250 |  | 0.0992 |  | 7.00 |  | 1.2604 |  | 0.248 |  |
|  |  |  |  | - |  | b |  | 2 |  | -0.8516 |  | 0.2602 |  | 7.00 |  | -3.2723 |  | 0.014 |  |
|  |  |  |  | - |  | b |  | 3 |  | -1.3398 |  | 0.4494 |  | 7.00 |  | -2.9812 |  | 0.020 |  |
|  |  |  |  | - |  | b |  | 4 |  | -0.9297 |  | 0.2320 |  | 7.00 |  | -4.0066 |  | 0.005 |  |
|  |  |  |  | - |  | b |  | 5 |  | -0.6992 |  | 0.2816 |  | 7.00 |  | -2.4834 |  | 0.042 |  |
|  |  |  |  | - |  | x |  | 1 |  | -0.7187 |  | 0.1537 |  | 7.00 |  | -4.6775 |  | 0.002 |  |
|  |  |  |  | - |  | x |  | 2 |  | -2.0743 |  | 0.5034 |  | 7.00 |  | -4.1208 |  | 0.004 |  |
|  |  |  |  | - |  | x |  | 3 |  | -3.5508 |  | 0.7856 |  | 7.00 |  | -4.5200 |  | 0.003 |  |
|  |  |  |  | - |  | x |  | 4 |  | -3.5117 |  | 0.5980 |  | 7.00 |  | -5.8728 |  | < .001 |  |
|  |  |  |  | - |  | x |  | 5 |  | -3.3203 |  | 0.5371 |  | 7.00 |  | -6.1817 |  | < .001 |  |
|  |  |  |  | - |  | y |  | 1 |  | -0.3828 |  | 0.0818 |  | 7.00 |  | -4.6811 |  | 0.002 |  |
|  |  |  |  | - |  | y |  | 2 |  | -1.0977 |  | 0.2203 |  | 7.00 |  | -4.9827 |  | 0.002 |  |
|  |  |  |  | - |  | y |  | 3 |  | -1.6055 |  | 0.4908 |  | 7.00 |  | -3.2708 |  | 0.014 |  |
|  |  |  |  | - |  | y |  | 4 |  | -1.6914 |  | 0.4345 |  | 7.00 |  | -3.8926 |  | 0.006 |  |
|  |  |  |  | - |  | y |  | 5 |  | -1.8398 |  | 0.4368 |  | 7.00 |  | -4.2117 |  | 0.004 |  |
|  |  | 2 |  | - |  | a |  | 3 |  | -0.9805 |  | 0.3209 |  | 7.00 |  | -3.0552 |  | 0.018 |  |
|  |  |  |  | - |  | a |  | 4 |  | -1.6484 |  | 0.4571 |  | 7.00 |  | -3.6060 |  | 0.009 |  |
|  |  |  |  | - |  | a |  | 5 |  | -1.3711 |  | 0.7331 |  | 7.00 |  | -1.8703 |  | 0.104 |  |
|  |  |  |  | - |  | b |  | 1 |  | 1.9102 |  | 0.2768 |  | 7.00 |  | 6.8998 |  | < .001 |  |
|  |  |  |  | - |  | b |  | 2 |  | 0.9336 |  | 0.1590 |  | 7.00 |  | 5.8734 |  | < .001 |  |
|  |  |  |  | - |  | b |  | 3 |  | 0.4453 |  | 0.3745 |  | 7.00 |  | 1.1890 |  | 0.273 |  |
|  |  |  |  | - |  | b |  | 4 |  | 0.8555 |  | 0.3943 |  | 7.00 |  | 2.1695 |  | 0.067 |  |
|  |  |  |  | - |  | b |  | 5 |  | 1.0859 |  | 0.3853 |  | 7.00 |  | 2.8187 |  | 0.026 |  |
|  |  |  |  | - |  | x |  | 1 |  | 1.0664 |  | 0.3962 |  | 7.00 |  | 2.6914 |  | 0.031 |  |
|  |  |  |  | - |  | x |  | 2 |  | -0.2891 |  | 0.3870 |  | 7.00 |  | -0.7470 |  | 0.479 |  |
|  |  |  |  | - |  | x |  | 3 |  | -1.7656 |  | 0.5532 |  | 7.00 |  | -3.1916 |  | 0.015 |  |
|  |  |  |  | - |  | x |  | 4 |  | -1.7266 |  | 0.4039 |  | 7.00 |  | -4.2746 |  | 0.004 |  |
|  |  |  |  | - |  | x |  | 5 |  | -1.5352 |  | 0.6202 |  | 7.00 |  | -2.4753 |  | 0.043 |  |
|  |  |  |  | - |  | y |  | 1 |  | 1.4023 |  | 0.3594 |  | 7.00 |  | 3.9014 |  | 0.006 |  |
|  |  |  |  | - |  | y |  | 2 |  | 0.6875 |  | 0.2423 |  | 7.00 |  | 2.8377 |  | 0.025 |  |
|  |  |  |  | - |  | y |  | 3 |  | 0.1797 |  | 0.4704 |  | 7.00 |  | 0.3819 |  | 0.714 |  |
|  |  |  |  | - |  | y |  | 4 |  | 0.0938 |  | 0.3924 |  | 7.00 |  | 0.2389 |  | 0.818 |  |
|  |  |  |  | - |  | y |  | 5 |  | -0.0547 |  | 0.4206 |  | 7.00 |  | -0.1300 |  | 0.900 |  |
|  |  | 3 |  | - |  | a |  | 4 |  | -0.6680 |  | 0.4649 |  | 7.00 |  | -1.4369 |  | 0.194 |  |
|  |  |  |  | - |  | a |  | 5 |  | -0.3906 |  | 0.7678 |  | 7.00 |  | -0.5088 |  | 0.627 |  |
|  |  |  |  | - |  | b |  | 1 |  | 2.8906 |  | 0.5155 |  | 7.00 |  | 5.6075 |  | < .001 |  |
|  |  |  |  | - |  | b |  | 2 |  | 1.9141 |  | 0.4180 |  | 7.00 |  | 4.5786 |  | 0.003 |  |
|  |  |  |  | - |  | b |  | 3 |  | 1.4258 |  | 0.4036 |  | 7.00 |  | 3.5329 |  | 0.010 |  |
|  |  |  |  | - |  | b |  | 4 |  | 1.8359 |  | 0.5519 |  | 7.00 |  | 3.3265 |  | 0.013 |  |
|  |  |  |  | - |  | b |  | 5 |  | 2.0664 |  | 0.5052 |  | 7.00 |  | 4.0907 |  | 0.005 |  |
|  |  |  |  | - |  | x |  | 1 |  | 2.0469 |  | 0.5830 |  | 7.00 |  | 3.5108 |  | 0.010 |  |
|  |  |  |  | - |  | x |  | 2 |  | 0.6913 |  | 0.4051 |  | 7.00 |  | 1.7066 |  | 0.132 |  |
|  |  |  |  | - |  | x |  | 3 |  | -0.7852 |  | 0.3014 |  | 7.00 |  | -2.6051 |  | 0.035 |  |
|  |  |  |  | - |  | x |  | 4 |  | -0.7461 |  | 0.1996 |  | 7.00 |  | -3.7377 |  | 0.007 |  |
|  |  |  |  | - |  | x |  | 5 |  | -0.5547 |  | 0.6178 |  | 7.00 |  | -0.8978 |  | 0.399 |  |
|  |  |  |  | - |  | y |  | 1 |  | 2.3828 |  | 0.5670 |  | 7.00 |  | 4.2027 |  | 0.004 |  |
|  |  |  |  | - |  | y |  | 2 |  | 1.6680 |  | 0.4430 |  | 7.00 |  | 3.7651 |  | 0.007 |  |
|  |  |  |  | - |  | y |  | 3 |  | 1.1602 |  | 0.4654 |  | 7.00 |  | 2.4928 |  | 0.041 |  |
|  |  |  |  | - |  | y |  | 4 |  | 1.0742 |  | 0.4534 |  | 7.00 |  | 2.3693 |  | 0.050 |  |
|  |  |  |  | - |  | y |  | 5 |  | 0.9258 |  | 0.3761 |  | 7.00 |  | 2.4618 |  | 0.043 |  |
|  |  | 4 |  | - |  | a |  | 5 |  | 0.2773 |  | 0.4414 |  | 7.00 |  | 0.6283 |  | 0.550 |  |
|  |  |  |  | - |  | b |  | 1 |  | 3.5586 |  | 0.4436 |  | 7.00 |  | 8.0226 |  | < .001 |  |
|  |  |  |  | - |  | b |  | 2 |  | 2.5820 |  | 0.4695 |  | 7.00 |  | 5.4992 |  | < .001 |  |
|  |  |  |  | - |  | b |  | 3 |  | 2.0937 |  | 0.3526 |  | 7.00 |  | 5.9386 |  | < .001 |  |
|  |  |  |  | - |  | b |  | 4 |  | 2.5039 |  | 0.3566 |  | 7.00 |  | 7.0222 |  | < .001 |  |
|  |  |  |  | - |  | b |  | 5 |  | 2.7344 |  | 0.3550 |  | 7.00 |  | 7.7029 |  | < .001 |  |
|  |  |  |  | - |  | x |  | 1 |  | 2.7148 |  | 0.4629 |  | 7.00 |  | 5.8655 |  | < .001 |  |
|  |  |  |  | - |  | x |  | 2 |  | 1.3593 |  | 0.5602 |  | 7.00 |  | 2.4265 |  | 0.046 |  |
|  |  |  |  | - |  | x |  | 3 |  | -0.1172 |  | 0.5957 |  | 7.00 |  | -0.1967 |  | 0.850 |  |
|  |  |  |  | - |  | x |  | 4 |  | -0.0781 |  | 0.3825 |  | 7.00 |  | -0.2043 |  | 0.844 |  |
|  |  |  |  | - |  | x |  | 5 |  | 0.1133 |  | 0.4545 |  | 7.00 |  | 0.2492 |  | 0.810 |  |
|  |  |  |  | - |  | y |  | 1 |  | 3.0508 |  | 0.4847 |  | 7.00 |  | 6.2942 |  | < .001 |  |
|  |  |  |  | - |  | y |  | 2 |  | 2.3359 |  | 0.4423 |  | 7.00 |  | 5.2810 |  | 0.001 |  |
|  |  |  |  | - |  | y |  | 3 |  | 1.8281 |  | 0.4327 |  | 7.00 |  | 4.2246 |  | 0.004 |  |
|  |  |  |  | - |  | y |  | 4 |  | 1.7422 |  | 0.2660 |  | 7.00 |  | 6.5495 |  | < .001 |  |
|  |  |  |  | - |  | y |  | 5 |  | 1.5937 |  | 0.3242 |  | 7.00 |  | 4.9164 |  | 0.002 |  |
|  |  | 5 |  | - |  | b |  | 1 |  | 3.2813 |  | 0.5922 |  | 7.00 |  | 5.5411 |  | < .001 |  |
|  |  |  |  | - |  | b |  | 2 |  | 2.3047 |  | 0.7110 |  | 7.00 |  | 3.2413 |  | 0.014 |  |
|  |  |  |  | - |  | b |  | 3 |  | 1.8164 |  | 0.6298 |  | 7.00 |  | 2.8840 |  | 0.024 |  |
|  |  |  |  | - |  | b |  | 4 |  | 2.2266 |  | 0.4770 |  | 7.00 |  | 4.6682 |  | 0.002 |  |
|  |  |  |  | - |  | b |  | 5 |  | 2.4570 |  | 0.4615 |  | 7.00 |  | 5.3243 |  | 0.001 |  |
|  |  |  |  | - |  | x |  | 1 |  | 2.4375 |  | 0.5188 |  | 7.00 |  | 4.6984 |  | 0.002 |  |
|  |  |  |  | - |  | x |  | 2 |  | 1.0820 |  | 0.7167 |  | 7.00 |  | 1.5096 |  | 0.175 |  |
|  |  |  |  | - |  | x |  | 3 |  | -0.3945 |  | 0.9064 |  | 7.00 |  | -0.4353 |  | 0.676 |  |
|  |  |  |  | - |  | x |  | 4 |  | -0.3555 |  | 0.7087 |  | 7.00 |  | -0.5016 |  | 0.631 |  |
|  |  |  |  | - |  | x |  | 5 |  | -0.1641 |  | 0.3082 |  | 7.00 |  | -0.5324 |  | 0.611 |  |
|  |  |  |  | - |  | y |  | 1 |  | 2.7734 |  | 0.5675 |  | 7.00 |  | 4.8869 |  | 0.002 |  |
|  |  |  |  | - |  | y |  | 2 |  | 2.0586 |  | 0.6264 |  | 7.00 |  | 3.2862 |  | 0.013 |  |
|  |  |  |  | - |  | y |  | 3 |  | 1.5508 |  | 0.6436 |  | 7.00 |  | 2.4096 |  | 0.047 |  |
|  |  |  |  | - |  | y |  | 4 |  | 1.4648 |  | 0.5406 |  | 7.00 |  | 2.7098 |  | 0.030 |  |
|  |  |  |  | - |  | y |  | 5 |  | 1.3164 |  | 0.4598 |  | 7.00 |  | 2.8633 |  | 0.024 |  |
| b |  | 1 |  | - |  | b |  | 2 |  | -0.9766 |  | 0.2154 |  | 7.00 |  | -4.5329 |  | 0.003 |  |
|  |  |  |  | - |  | b |  | 3 |  | -1.4648 |  | 0.4247 |  | 7.00 |  | -3.4495 |  | 0.011 |  |
|  |  |  |  | - |  | b |  | 4 |  | -1.0547 |  | 0.2197 |  | 7.00 |  | -4.8009 |  | 0.002 |  |
|  |  |  |  | - |  | b |  | 5 |  | -0.8242 |  | 0.2873 |  | 7.00 |  | -2.8687 |  | 0.024 |  |
|  |  |  |  | - |  | x |  | 1 |  | -0.8438 |  | 0.1702 |  | 7.00 |  | -4.9561 |  | 0.002 |  |
|  |  |  |  | - |  | x |  | 2 |  | -2.1993 |  | 0.5199 |  | 7.00 |  | -4.2304 |  | 0.004 |  |
|  |  |  |  | - |  | x |  | 3 |  | -3.6758 |  | 0.7553 |  | 7.00 |  | -4.8668 |  | 0.002 |  |
|  |  |  |  | - |  | x |  | 4 |  | -3.6367 |  | 0.5497 |  | 7.00 |  | -6.6158 |  | < .001 |  |
|  |  |  |  | - |  | x |  | 5 |  | -3.4453 |  | 0.5375 |  | 7.00 |  | -6.4098 |  | < .001 |  |
|  |  |  |  | - |  | y |  | 1 |  | -0.5078 |  | 0.1279 |  | 7.00 |  | -3.9716 |  | 0.005 |  |
|  |  |  |  | - |  | y |  | 2 |  | -1.2227 |  | 0.2331 |  | 7.00 |  | -5.2443 |  | 0.001 |  |
|  |  |  |  | - |  | y |  | 3 |  | -1.7305 |  | 0.4875 |  | 7.00 |  | -3.5500 |  | 0.009 |  |
|  |  |  |  | - |  | y |  | 4 |  | -1.8164 |  | 0.4114 |  | 7.00 |  | -4.4149 |  | 0.003 |  |
|  |  |  |  | - |  | y |  | 5 |  | -1.9648 |  | 0.4334 |  | 7.00 |  | -4.5332 |  | 0.003 |  |
|  |  | 2 |  | - |  | b |  | 3 |  | -0.4883 |  | 0.3275 |  | 7.00 |  | -1.4908 |  | 0.180 |  |
|  |  |  |  | - |  | b |  | 4 |  | -0.0781 |  | 0.3781 |  | 7.00 |  | -0.2066 |  | 0.842 |  |
|  |  |  |  | - |  | b |  | 5 |  | 0.1523 |  | 0.4131 |  | 7.00 |  | 0.3688 |  | 0.723 |  |
|  |  |  |  | - |  | x |  | 1 |  | 0.1328 |  | 0.3020 |  | 7.00 |  | 0.4398 |  | 0.673 |  |
|  |  |  |  | - |  | x |  | 2 |  | -1.2227 |  | 0.5000 |  | 7.00 |  | -2.4456 |  | 0.044 |  |
|  |  |  |  | - |  | x |  | 3 |  | -2.6992 |  | 0.6166 |  | 7.00 |  | -4.3778 |  | 0.003 |  |
|  |  |  |  | - |  | x |  | 4 |  | -2.6602 |  | 0.4541 |  | 7.00 |  | -5.8580 |  | < .001 |  |
|  |  |  |  | - |  | x |  | 5 |  | -2.4687 |  | 0.5981 |  | 7.00 |  | -4.1276 |  | 0.004 |  |
|  |  |  |  | - |  | y |  | 1 |  | 0.4687 |  | 0.2770 |  | 7.00 |  | 1.6922 |  | 0.134 |  |
|  |  |  |  | - |  | y |  | 2 |  | -0.2461 |  | 0.2681 |  | 7.00 |  | -0.9178 |  | 0.389 |  |
|  |  |  |  | - |  | y |  | 3 |  | -0.7539 |  | 0.4322 |  | 7.00 |  | -1.7444 |  | 0.125 |  |
|  |  |  |  | - |  | y |  | 4 |  | -0.8398 |  | 0.4008 |  | 7.00 |  | -2.0953 |  | 0.074 |  |
|  |  |  |  | - |  | y |  | 5 |  | -0.9883 |  | 0.4314 |  | 7.00 |  | -2.2909 |  | 0.056 |  |
|  |  | 3 |  | - |  | b |  | 4 |  | 0.4102 |  | 0.4350 |  | 7.00 |  | 0.9430 |  | 0.377 |  |
|  |  |  |  | - |  | b |  | 5 |  | 0.6406 |  | 0.4512 |  | 7.00 |  | 1.4198 |  | 0.199 |  |
|  |  |  |  | - |  | x |  | 1 |  | 0.6211 |  | 0.4138 |  | 7.00 |  | 1.5010 |  | 0.177 |  |
|  |  |  |  | - |  | x |  | 2 |  | -0.7344 |  | 0.5451 |  | 7.00 |  | -1.3472 |  | 0.220 |  |
|  |  |  |  | - |  | x |  | 3 |  | -2.2109 |  | 0.4766 |  | 7.00 |  | -4.6386 |  | 0.002 |  |
|  |  |  |  | - |  | x |  | 4 |  | -2.1719 |  | 0.3623 |  | 7.00 |  | -5.9951 |  | < .001 |  |
|  |  |  |  | - |  | x |  | 5 |  | -1.9805 |  | 0.5556 |  | 7.00 |  | -3.5643 |  | 0.009 |  |
|  |  |  |  | - |  | y |  | 1 |  | 0.9570 |  | 0.4227 |  | 7.00 |  | 2.2639 |  | 0.058 |  |
|  |  |  |  | - |  | y |  | 2 |  | 0.2422 |  | 0.3803 |  | 7.00 |  | 0.6368 |  | 0.545 |  |
|  |  |  |  | - |  | y |  | 3 |  | -0.2656 |  | 0.1976 |  | 7.00 |  | -1.3446 |  | 0.221 |  |
|  |  |  |  | - |  | y |  | 4 |  | -0.3516 |  | 0.2534 |  | 7.00 |  | -1.3871 |  | 0.208 |  |
|  |  |  |  | - |  | y |  | 5 |  | -0.5000 |  | 0.3092 |  | 7.00 |  | -1.6168 |  | 0.150 |  |
|  |  | 4 |  | - |  | b |  | 5 |  | 0.2305 |  | 0.1396 |  | 7.00 |  | 1.6507 |  | 0.143 |  |
|  |  |  |  | - |  | x |  | 1 |  | 0.2109 |  | 0.2459 |  | 7.00 |  | 0.8578 |  | 0.419 |  |
|  |  |  |  | - |  | x |  | 2 |  | -1.1446 |  | 0.5118 |  | 7.00 |  | -2.2364 |  | 0.060 |  |
|  |  |  |  | - |  | x |  | 3 |  | -2.6211 |  | 0.7828 |  | 7.00 |  | -3.3485 |  | 0.012 |  |
|  |  |  |  | - |  | x |  | 4 |  | -2.5820 |  | 0.5772 |  | 7.00 |  | -4.4730 |  | 0.003 |  |
|  |  |  |  | - |  | x |  | 5 |  | -2.3906 |  | 0.5281 |  | 7.00 |  | -4.5266 |  | 0.003 |  |
|  |  |  |  | - |  | y |  | 1 |  | 0.5469 |  | 0.2210 |  | 7.00 |  | 2.4749 |  | 0.043 |  |
|  |  |  |  | - |  | y |  | 2 |  | -0.1680 |  | 0.2403 |  | 7.00 |  | -0.6989 |  | 0.507 |  |
|  |  |  |  | - |  | y |  | 3 |  | -0.6758 |  | 0.4510 |  | 7.00 |  | -1.4985 |  | 0.178 |  |
|  |  |  |  | - |  | y |  | 4 |  | -0.7617 |  | 0.3203 |  | 7.00 |  | -2.3780 |  | 0.049 |  |
|  |  |  |  | - |  | y |  | 5 |  | -0.9102 |  | 0.3895 |  | 7.00 |  | -2.3368 |  | 0.052 |  |
|  |  | 5 |  | - |  | x |  | 1 |  | -0.0195 |  | 0.3278 |  | 7.00 |  | -0.0596 |  | 0.954 |  |
|  |  |  |  | - |  | x |  | 2 |  | -1.3751 |  | 0.3957 |  | 7.00 |  | -3.4747 |  | 0.010 |  |
|  |  |  |  | - |  | x |  | 3 |  | -2.8516 |  | 0.7364 |  | 7.00 |  | -3.8723 |  | 0.006 |  |
|  |  |  |  | - |  | x |  | 4 |  | -2.8125 |  | 0.5540 |  | 7.00 |  | -5.0770 |  | 0.001 |  |
|  |  |  |  | - |  | x |  | 5 |  | -2.6211 |  | 0.5053 |  | 7.00 |  | -5.1873 |  | 0.001 |  |
|  |  |  |  | - |  | y |  | 1 |  | 0.3164 |  | 0.2972 |  | 7.00 |  | 1.0646 |  | 0.322 |  |
|  |  |  |  | - |  | y |  | 2 |  | -0.3984 |  | 0.2144 |  | 7.00 |  | -1.8585 |  | 0.105 |  |
|  |  |  |  | - |  | y |  | 3 |  | -0.9063 |  | 0.4663 |  | 7.00 |  | -1.9434 |  | 0.093 |  |
|  |  |  |  | - |  | y |  | 4 |  | -0.9922 |  | 0.3070 |  | 7.00 |  | -3.2322 |  | 0.014 |  |
|  |  |  |  | - |  | y |  | 5 |  | -1.1406 |  | 0.3493 |  | 7.00 |  | -3.2651 |  | 0.014 |  |
| x |  | 1 |  | - |  | x |  | 2 |  | -1.3555 |  | 0.5941 |  | 7.00 |  | -2.2816 |  | 0.057 |  |
|  |  |  |  | - |  | x |  | 3 |  | -2.8320 |  | 0.7963 |  | 7.00 |  | -3.5566 |  | 0.009 |  |
|  |  |  |  | - |  | x |  | 4 |  | -2.7930 |  | 0.5893 |  | 7.00 |  | -4.7395 |  | 0.002 |  |
|  |  |  |  | - |  | x |  | 5 |  | -2.6016 |  | 0.4759 |  | 7.00 |  | -5.4666 |  | < .001 |  |
|  |  |  |  | - |  | y |  | 1 |  | 0.3359 |  | 0.0930 |  | 7.00 |  | 3.6139 |  | 0.009 |  |
|  |  |  |  | - |  | y |  | 2 |  | -0.3789 |  | 0.3112 |  | 7.00 |  | -1.2176 |  | 0.263 |  |
|  |  |  |  | - |  | y |  | 3 |  | -0.8867 |  | 0.4376 |  | 7.00 |  | -2.0262 |  | 0.082 |  |
|  |  |  |  | - |  | y |  | 4 |  | -0.9727 |  | 0.4174 |  | 7.00 |  | -2.3305 |  | 0.053 |  |
|  |  |  |  | - |  | y |  | 5 |  | -1.1211 |  | 0.3961 |  | 7.00 |  | -2.8300 |  | 0.025 |  |
|  |  | 2 |  | - |  | x |  | 3 |  | -1.4765 |  | 0.5878 |  | 7.00 |  | -2.5117 |  | 0.040 |  |
|  |  |  |  | - |  | x |  | 4 |  | -1.4374 |  | 0.5395 |  | 7.00 |  | -2.6643 |  | 0.032 |  |
|  |  |  |  | - |  | x |  | 5 |  | -1.2460 |  | 0.6448 |  | 7.00 |  | -1.9325 |  | 0.095 |  |
|  |  |  |  | - |  | y |  | 1 |  | 1.6915 |  | 0.5522 |  | 7.00 |  | 3.0634 |  | 0.018 |  |
|  |  |  |  | - |  | y |  | 2 |  | 0.9766 |  | 0.3459 |  | 7.00 |  | 2.8236 |  | 0.026 |  |
|  |  |  |  | - |  | y |  | 3 |  | 0.4688 |  | 0.5840 |  | 7.00 |  | 0.8027 |  | 0.449 |  |
|  |  |  |  | - |  | y |  | 4 |  | 0.3829 |  | 0.4663 |  | 7.00 |  | 0.8211 |  | 0.439 |  |
|  |  |  |  | - |  | y |  | 5 |  | 0.2344 |  | 0.4459 |  | 7.00 |  | 0.5258 |  | 0.615 |  |
|  |  | 3 |  | - |  | x |  | 4 |  | 0.0391 |  | 0.2839 |  | 7.00 |  | 0.1376 |  | 0.894 |  |
|  |  |  |  | - |  | x |  | 5 |  | 0.2305 |  | 0.7440 |  | 7.00 |  | 0.3098 |  | 0.766 |  |
|  |  |  |  | - |  | y |  | 1 |  | 3.1680 |  | 0.7933 |  | 7.00 |  | 3.9932 |  | 0.005 |  |
|  |  |  |  | - |  | y |  | 2 |  | 2.4531 |  | 0.6721 |  | 7.00 |  | 3.6502 |  | 0.008 |  |
|  |  |  |  | - |  | y |  | 3 |  | 1.9453 |  | 0.5519 |  | 7.00 |  | 3.5251 |  | 0.010 |  |
|  |  |  |  | - |  | y |  | 4 |  | 1.8594 |  | 0.5869 |  | 7.00 |  | 3.1680 |  | 0.016 |  |
|  |  |  |  | - |  | y |  | 5 |  | 1.7109 |  | 0.5243 |  | 7.00 |  | 3.2631 |  | 0.014 |  |
|  |  | 4 |  | - |  | x |  | 5 |  | 0.1914 |  | 0.5487 |  | 7.00 |  | 0.3488 |  | 0.737 |  |
|  |  |  |  | - |  | y |  | 1 |  | 3.1289 |  | 0.6008 |  | 7.00 |  | 5.2082 |  | 0.001 |  |
|  |  |  |  | - |  | y |  | 2 |  | 2.4141 |  | 0.5290 |  | 7.00 |  | 4.5634 |  | 0.003 |  |
|  |  |  |  | - |  | y |  | 3 |  | 1.9062 |  | 0.4510 |  | 7.00 |  | 4.2267 |  | 0.004 |  |
|  |  |  |  | - |  | y |  | 4 |  | 1.8203 |  | 0.4450 |  | 7.00 |  | 4.0906 |  | 0.005 |  |
|  |  |  |  | - |  | y |  | 5 |  | 1.6719 |  | 0.3546 |  | 7.00 |  | 4.7143 |  | 0.002 |  |
|  |  | 5 |  | - |  | y |  | 1 |  | 2.9375 |  | 0.5317 |  | 7.00 |  | 5.5246 |  | < .001 |  |
|  |  |  |  | - |  | y |  | 2 |  | 2.2227 |  | 0.5997 |  | 7.00 |  | 3.7065 |  | 0.008 |  |
|  |  |  |  | - |  | y |  | 3 |  | 1.7148 |  | 0.6070 |  | 7.00 |  | 2.8253 |  | 0.026 |  |
|  |  |  |  | - |  | y |  | 4 |  | 1.6289 |  | 0.5641 |  | 7.00 |  | 2.8877 |  | 0.023 |  |
|  |  |  |  | - |  | y |  | 5 |  | 1.4805 |  | 0.3727 |  | 7.00 |  | 3.9727 |  | 0.005 |  |
| y |  | 1 |  | - |  | y |  | 2 |  | -0.7148 |  | 0.2504 |  | 7.00 |  | -2.8550 |  | 0.025 |  |
|  |  |  |  | - |  | y |  | 3 |  | -1.2227 |  | 0.4479 |  | 7.00 |  | -2.7297 |  | 0.029 |  |
|  |  |  |  | - |  | y |  | 4 |  | -1.3086 |  | 0.4171 |  | 7.00 |  | -3.1376 |  | 0.016 |  |
|  |  |  |  | - |  | y |  | 5 |  | -1.4570 |  | 0.4222 |  | 7.00 |  | -3.4507 |  | 0.011 |  |
|  |  | 2 |  | - |  | y |  | 3 |  | -0.5078 |  | 0.4109 |  | 7.00 |  | -1.2359 |  | 0.256 |  |
|  |  |  |  | - |  | y |  | 4 |  | -0.5938 |  | 0.3044 |  | 7.00 |  | -1.9508 |  | 0.092 |  |
|  |  |  |  | - |  | y |  | 5 |  | -0.7422 |  | 0.3784 |  | 7.00 |  | -1.9615 |  | 0.091 |  |
|  |  | 3 |  | - |  | y |  | 4 |  | -0.0859 |  | 0.2688 |  | 7.00 |  | -0.3197 |  | 0.759 |  |
|  |  |  |  | - |  | y |  | 5 |  | -0.2344 |  | 0.2954 |  | 7.00 |  | -0.7934 |  | 0.454 |  |
|  |  | 4 |  | - |  | y |  | 5 |  | -0.1484 |  | 0.2797 |  | 7.00 |  | -0.5306 |  | 0.612 |  |
|  | | | | | | | | | | | | | | | | | | | |

**Compound phase (target trials X and Y)**

| Within Subjects Effects | | | | | | | | | | | | | | | |
| --- | --- | --- | --- | --- | --- | --- | --- | --- | --- | --- | --- | --- | --- | --- | --- |
|  |  |  |  |  |  |  |  |  |  |  |  |  |  |  |  |
|  | | **Sum of Squares** | | **df** | | **Mean Square** | | **F** | | **p** | | **η²** | | **η²_p_** | |
| stimulus |  | 3.31 |  | 1 |  | 3.306 |  | 0.659 |  | 0.444 |  | 0.012 |  | 0.086 |  |
| Residual |  | 35.11 |  | 7 |  | 5.016 |  |  |  |  |  |  |  |  |  |
| block |  | 8.76 |  | 9 |  | 0.973 |  | 1.184 |  | 0.321 |  | 0.031 |  | 0.145 |  |
| Residual |  | 51.80 |  | 63 |  | 0.822 |  |  |  |  |  |  |  |  |  |
| stimulus ✻ block |  | 37.80 |  | 9 |  | 4.200 |  | 4.449 |  | < .001 |  | 0.134 |  | 0.389 |  |
| Residual |  | 59.47 |  | 63 |  | 0.944 |  |  |  |  |  |  |  |  |  |
| Note. Type 3 Sums of Squares | | | | | | | | | | | | | | | |
|  | | | | | | | | | | | | | | | |

| Post Hoc Comparisons - stimulus ✻ block | | | | | | | | | | | | | | | | | | | |
| --- | --- | --- | --- | --- | --- | --- | --- | --- | --- | --- | --- | --- | --- | --- | --- | --- | --- | --- | --- |
| **Comparison** | | | | | | | | | |  | | | | | | | | | |
| **stimulus** | | **block** | |  | | **stimulus** | | **block** | | **Mean Difference** | | **SE** | | **df** | | **t** | | **p** | |
| X |  | 1 |  | - |  | X |  | 2 |  | 0.0833 |  | 0.518 |  | 7.00 |  | 0.1609 |  | 0.877 |  |
|  |  |  |  | - |  | X |  | 3 |  | -0.2813 |  | 0.577 |  | 7.00 |  | -0.4872 |  | 0.641 |  |
|  |  |  |  | - |  | X |  | 4 |  | -0.3437 |  | 0.381 |  | 7.00 |  | -0.9021 |  | 0.397 |  |
|  |  |  |  | - |  | X |  | 5 |  | -0.0625 |  | 0.515 |  | 7.00 |  | -0.1215 |  | 0.907 |  |
|  |  |  |  | - |  | X |  | 6 |  | 0.2813 |  | 0.608 |  | 7.00 |  | 0.4628 |  | 0.658 |  |
|  |  |  |  | - |  | X |  | 7 |  | 0.4687 |  | 0.509 |  | 7.00 |  | 0.9203 |  | 0.388 |  |
|  |  |  |  | - |  | X |  | 8 |  | 0.8958 |  | 0.445 |  | 7.00 |  | 2.0127 |  | 0.084 |  |
|  |  |  |  | - |  | X |  | 9 |  | 1.2396 |  | 0.588 |  | 7.00 |  | 2.1093 |  | 0.073 |  |
|  |  |  |  | - |  | X |  | 10 |  | 0.9896 |  | 0.322 |  | 7.00 |  | 3.0755 |  | 0.018 |  |
|  |  |  |  | - |  | Y |  | 1 |  | 1.1354 |  | 0.437 |  | 7.00 |  | 2.6000 |  | 0.035 |  |
|  |  |  |  | - |  | Y |  | 2 |  | 0.9167 |  | 0.600 |  | 7.00 |  | 1.5275 |  | 0.170 |  |
|  |  |  |  | - |  | Y |  | 3 |  | 0.0312 |  | 0.446 |  | 7.00 |  | 0.0701 |  | 0.946 |  |
|  |  |  |  | - |  | Y |  | 4 |  | 0.3125 |  | 0.607 |  | 7.00 |  | 0.5150 |  | 0.622 |  |
|  |  |  |  | - |  | Y |  | 5 |  | -0.1979 |  | 0.665 |  | 7.00 |  | -0.2976 |  | 0.775 |  |
|  |  |  |  | - |  | Y |  | 6 |  | -0.2292 |  | 0.586 |  | 7.00 |  | -0.3908 |  | 0.708 |  |
|  |  |  |  | - |  | Y |  | 7 |  | -0.1563 |  | 0.536 |  | 7.00 |  | -0.2914 |  | 0.779 |  |
|  |  |  |  | - |  | Y |  | 8 |  | -0.2604 |  | 0.666 |  | 7.00 |  | -0.3911 |  | 0.707 |  |
|  |  |  |  | - |  | Y |  | 9 |  | -0.5729 |  | 0.575 |  | 7.00 |  | -0.9959 |  | 0.352 |  |
|  |  |  |  | - |  | Y |  | 10 |  | -0.5833 |  | 0.976 |  | 7.00 |  | -0.5976 |  | 0.569 |  |
|  |  | 2 |  | - |  | X |  | 3 |  | -0.3646 |  | 0.176 |  | 7.00 |  | -2.0711 |  | 0.077 |  |
|  |  |  |  | - |  | X |  | 4 |  | -0.4271 |  | 0.280 |  | 7.00 |  | -1.5269 |  | 0.171 |  |
|  |  |  |  | - |  | X |  | 5 |  | -0.1458 |  | 0.268 |  | 7.00 |  | -0.5449 |  | 0.603 |  |
|  |  |  |  | - |  | X |  | 6 |  | 0.1979 |  | 0.273 |  | 7.00 |  | 0.7244 |  | 0.492 |  |
|  |  |  |  | - |  | X |  | 7 |  | 0.3854 |  | 0.317 |  | 7.00 |  | 1.2147 |  | 0.264 |  |
|  |  |  |  | - |  | X |  | 8 |  | 0.8125 |  | 0.473 |  | 7.00 |  | 1.7171 |  | 0.130 |  |
|  |  |  |  | - |  | X |  | 9 |  | 1.1563 |  | 0.633 |  | 7.00 |  | 1.8256 |  | 0.111 |  |
|  |  |  |  | - |  | X |  | 10 |  | 0.9062 |  | 0.455 |  | 7.00 |  | 1.9909 |  | 0.087 |  |
|  |  |  |  | - |  | Y |  | 1 |  | 1.0521 |  | 0.326 |  | 7.00 |  | 3.2294 |  | 0.014 |  |
|  |  |  |  | - |  | Y |  | 2 |  | 0.8333 |  | 0.380 |  | 7.00 |  | 2.1953 |  | 0.064 |  |
|  |  |  |  | - |  | Y |  | 3 |  | -0.0521 |  | 0.370 |  | 7.00 |  | -0.1409 |  | 0.892 |  |
|  |  |  |  | - |  | Y |  | 4 |  | 0.2292 |  | 0.371 |  | 7.00 |  | 0.6184 |  | 0.556 |  |
|  |  |  |  | - |  | Y |  | 5 |  | -0.2812 |  | 0.480 |  | 7.00 |  | -0.5863 |  | 0.576 |  |
|  |  |  |  | - |  | Y |  | 6 |  | -0.3125 |  | 0.528 |  | 7.00 |  | -0.5917 |  | 0.573 |  |
|  |  |  |  | - |  | Y |  | 7 |  | -0.2396 |  | 0.480 |  | 7.00 |  | -0.4990 |  | 0.633 |  |
|  |  |  |  | - |  | Y |  | 8 |  | -0.3438 |  | 0.550 |  | 7.00 |  | -0.6251 |  | 0.552 |  |
|  |  |  |  | - |  | Y |  | 9 |  | -0.6563 |  | 0.639 |  | 7.00 |  | -1.0276 |  | 0.338 |  |
|  |  |  |  | - |  | Y |  | 10 |  | -0.6667 |  | 0.900 |  | 7.00 |  | -0.7404 |  | 0.483 |  |
|  |  | 3 |  | - |  | X |  | 4 |  | -0.0625 |  | 0.361 |  | 7.00 |  | -0.1732 |  | 0.867 |  |
|  |  |  |  | - |  | X |  | 5 |  | 0.2188 |  | 0.300 |  | 7.00 |  | 0.7291 |  | 0.490 |  |
|  |  |  |  | - |  | X |  | 6 |  | 0.5625 |  | 0.267 |  | 7.00 |  | 2.1093 |  | 0.073 |  |
|  |  |  |  | - |  | X |  | 7 |  | 0.7500 |  | 0.336 |  | 7.00 |  | 2.2326 |  | 0.061 |  |
|  |  |  |  | - |  | X |  | 8 |  | 1.1771 |  | 0.464 |  | 7.00 |  | 2.5348 |  | 0.039 |  |
|  |  |  |  | - |  | X |  | 9 |  | 1.5208 |  | 0.690 |  | 7.00 |  | 2.2035 |  | 0.063 |  |
|  |  |  |  | - |  | X |  | 10 |  | 1.2708 |  | 0.437 |  | 7.00 |  | 2.9085 |  | 0.023 |  |
|  |  |  |  | - |  | Y |  | 1 |  | 1.4167 |  | 0.388 |  | 7.00 |  | 3.6482 |  | 0.008 |  |
|  |  |  |  | - |  | Y |  | 2 |  | 1.1979 |  | 0.443 |  | 7.00 |  | 2.7030 |  | 0.031 |  |
|  |  |  |  | - |  | Y |  | 3 |  | 0.3125 |  | 0.425 |  | 7.00 |  | 0.7346 |  | 0.486 |  |
|  |  |  |  | - |  | Y |  | 4 |  | 0.5938 |  | 0.379 |  | 7.00 |  | 1.5676 |  | 0.161 |  |
|  |  |  |  | - |  | Y |  | 5 |  | 0.0833 |  | 0.549 |  | 7.00 |  | 0.1519 |  | 0.884 |  |
|  |  |  |  | - |  | Y |  | 6 |  | 0.0521 |  | 0.604 |  | 7.00 |  | 0.0863 |  | 0.934 |  |
|  |  |  |  | - |  | Y |  | 7 |  | 0.1250 |  | 0.497 |  | 7.00 |  | 0.2515 |  | 0.809 |  |
|  |  |  |  | - |  | Y |  | 8 |  | 0.0208 |  | 0.499 |  | 7.00 |  | 0.0418 |  | 0.968 |  |
|  |  |  |  | - |  | Y |  | 9 |  | -0.2917 |  | 0.668 |  | 7.00 |  | -0.4364 |  | 0.676 |  |
|  |  |  |  | - |  | Y |  | 10 |  | -0.3021 |  | 0.921 |  | 7.00 |  | -0.3280 |  | 0.753 |  |
|  |  | 4 |  | - |  | X |  | 5 |  | 0.2812 |  | 0.286 |  | 7.00 |  | 0.9832 |  | 0.358 |  |
|  |  |  |  | - |  | X |  | 6 |  | 0.6250 |  | 0.377 |  | 7.00 |  | 1.6579 |  | 0.141 |  |
|  |  |  |  | - |  | X |  | 7 |  | 0.8125 |  | 0.298 |  | 7.00 |  | 2.7277 |  | 0.029 |  |
|  |  |  |  | - |  | X |  | 8 |  | 1.2396 |  | 0.369 |  | 7.00 |  | 3.3610 |  | 0.012 |  |
|  |  |  |  | - |  | X |  | 9 |  | 1.5833 |  | 0.483 |  | 7.00 |  | 3.2757 |  | 0.014 |  |
|  |  |  |  | - |  | X |  | 10 |  | 1.3333 |  | 0.343 |  | 7.00 |  | 3.8887 |  | 0.006 |  |
|  |  |  |  | - |  | Y |  | 1 |  | 1.4792 |  | 0.286 |  | 7.00 |  | 5.1723 |  | 0.001 |  |
|  |  |  |  | - |  | Y |  | 2 |  | 1.2604 |  | 0.440 |  | 7.00 |  | 2.8667 |  | 0.024 |  |
|  |  |  |  | - |  | Y |  | 3 |  | 0.3750 |  | 0.351 |  | 7.00 |  | 1.0681 |  | 0.321 |  |
|  |  |  |  | - |  | Y |  | 4 |  | 0.6562 |  | 0.433 |  | 7.00 |  | 1.5141 |  | 0.174 |  |
|  |  |  |  | - |  | Y |  | 5 |  | 0.1458 |  | 0.559 |  | 7.00 |  | 0.2607 |  | 0.802 |  |
|  |  |  |  | - |  | Y |  | 6 |  | 0.1146 |  | 0.561 |  | 7.00 |  | 0.2043 |  | 0.844 |  |
|  |  |  |  | - |  | Y |  | 7 |  | 0.1875 |  | 0.567 |  | 7.00 |  | 0.3308 |  | 0.751 |  |
|  |  |  |  | - |  | Y |  | 8 |  | 0.0833 |  | 0.650 |  | 7.00 |  | 0.1283 |  | 0.902 |  |
|  |  |  |  | - |  | Y |  | 9 |  | -0.2292 |  | 0.644 |  | 7.00 |  | -0.3557 |  | 0.733 |  |
|  |  |  |  | - |  | Y |  | 10 |  | -0.2396 |  | 0.913 |  | 7.00 |  | -0.2623 |  | 0.801 |  |
|  |  | 5 |  | - |  | X |  | 6 |  | 0.3438 |  | 0.291 |  | 7.00 |  | 1.1813 |  | 0.276 |  |
|  |  |  |  | - |  | X |  | 7 |  | 0.5312 |  | 0.283 |  | 7.00 |  | 1.8743 |  | 0.103 |  |
|  |  |  |  | - |  | X |  | 8 |  | 0.9583 |  | 0.274 |  | 7.00 |  | 3.5017 |  | 0.010 |  |
|  |  |  |  | - |  | X |  | 9 |  | 1.3021 |  | 0.509 |  | 7.00 |  | 2.5577 |  | 0.038 |  |
|  |  |  |  | - |  | X |  | 10 |  | 1.0521 |  | 0.353 |  | 7.00 |  | 2.9789 |  | 0.021 |  |
|  |  |  |  | - |  | Y |  | 1 |  | 1.1979 |  | 0.241 |  | 7.00 |  | 4.9732 |  | 0.002 |  |
|  |  |  |  | - |  | Y |  | 2 |  | 0.9792 |  | 0.307 |  | 7.00 |  | 3.1906 |  | 0.015 |  |
|  |  |  |  | - |  | Y |  | 3 |  | 0.0937 |  | 0.235 |  | 7.00 |  | 0.3982 |  | 0.702 |  |
|  |  |  |  | - |  | Y |  | 4 |  | 0.3750 |  | 0.155 |  | 7.00 |  | 2.4177 |  | 0.046 |  |
|  |  |  |  | - |  | Y |  | 5 |  | -0.1354 |  | 0.395 |  | 7.00 |  | -0.3429 |  | 0.742 |  |
|  |  |  |  | - |  | Y |  | 6 |  | -0.1667 |  | 0.416 |  | 7.00 |  | -0.4006 |  | 0.701 |  |
|  |  |  |  | - |  | Y |  | 7 |  | -0.0938 |  | 0.445 |  | 7.00 |  | -0.2105 |  | 0.839 |  |
|  |  |  |  | - |  | Y |  | 8 |  | -0.1979 |  | 0.534 |  | 7.00 |  | -0.3706 |  | 0.722 |  |
|  |  |  |  | - |  | Y |  | 9 |  | -0.5104 |  | 0.560 |  | 7.00 |  | -0.9107 |  | 0.393 |  |
|  |  |  |  | - |  | Y |  | 10 |  | -0.5208 |  | 0.700 |  | 7.00 |  | -0.7440 |  | 0.481 |  |
|  |  | 6 |  | - |  | X |  | 7 |  | 0.1875 |  | 0.163 |  | 7.00 |  | 1.1523 |  | 0.287 |  |
|  |  |  |  | - |  | X |  | 8 |  | 0.6146 |  | 0.417 |  | 7.00 |  | 1.4730 |  | 0.184 |  |
|  |  |  |  | - |  | X |  | 9 |  | 0.9583 |  | 0.534 |  | 7.00 |  | 1.7952 |  | 0.116 |  |
|  |  |  |  | - |  | X |  | 10 |  | 0.7083 |  | 0.425 |  | 7.00 |  | 1.6681 |  | 0.139 |  |
|  |  |  |  | - |  | Y |  | 1 |  | 0.8542 |  | 0.379 |  | 7.00 |  | 2.2526 |  | 0.059 |  |
|  |  |  |  | - |  | Y |  | 2 |  | 0.6354 |  | 0.393 |  | 7.00 |  | 1.6166 |  | 0.150 |  |
|  |  |  |  | - |  | Y |  | 3 |  | -0.2500 |  | 0.375 |  | 7.00 |  | -0.6667 |  | 0.526 |  |
|  |  |  |  | - |  | Y |  | 4 |  | 0.0312 |  | 0.374 |  | 7.00 |  | 0.0836 |  | 0.936 |  |
|  |  |  |  | - |  | Y |  | 5 |  | -0.4792 |  | 0.617 |  | 7.00 |  | -0.7772 |  | 0.463 |  |
|  |  |  |  | - |  | Y |  | 6 |  | -0.5104 |  | 0.606 |  | 7.00 |  | -0.8426 |  | 0.427 |  |
|  |  |  |  | - |  | Y |  | 7 |  | -0.4375 |  | 0.543 |  | 7.00 |  | -0.8054 |  | 0.447 |  |
|  |  |  |  | - |  | Y |  | 8 |  | -0.5417 |  | 0.554 |  | 7.00 |  | -0.9779 |  | 0.361 |  |
|  |  |  |  | - |  | Y |  | 9 |  | -0.8542 |  | 0.684 |  | 7.00 |  | -1.2487 |  | 0.252 |  |
|  |  |  |  | - |  | Y |  | 10 |  | -0.8646 |  | 0.934 |  | 7.00 |  | -0.9261 |  | 0.385 |  |
|  |  | 7 |  | - |  | X |  | 8 |  | 0.4271 |  | 0.320 |  | 7.00 |  | 1.3353 |  | 0.224 |  |
|  |  |  |  | - |  | X |  | 9 |  | 0.7708 |  | 0.416 |  | 7.00 |  | 1.8530 |  | 0.106 |  |
|  |  |  |  | - |  | X |  | 10 |  | 0.5208 |  | 0.328 |  | 7.00 |  | 1.5880 |  | 0.156 |  |
|  |  |  |  | - |  | Y |  | 1 |  | 0.6667 |  | 0.326 |  | 7.00 |  | 2.0438 |  | 0.080 |  |
|  |  |  |  | - |  | Y |  | 2 |  | 0.4479 |  | 0.369 |  | 7.00 |  | 1.2128 |  | 0.265 |  |
|  |  |  |  | - |  | Y |  | 3 |  | -0.4375 |  | 0.356 |  | 7.00 |  | -1.2304 |  | 0.258 |  |
|  |  |  |  | - |  | Y |  | 4 |  | -0.1562 |  | 0.396 |  | 7.00 |  | -0.3942 |  | 0.705 |  |
|  |  |  |  | - |  | Y |  | 5 |  | -0.6667 |  | 0.648 |  | 7.00 |  | -1.0285 |  | 0.338 |  |
|  |  |  |  | - |  | Y |  | 6 |  | -0.6979 |  | 0.617 |  | 7.00 |  | -1.1304 |  | 0.296 |  |
|  |  |  |  | - |  | Y |  | 7 |  | -0.6250 |  | 0.573 |  | 7.00 |  | -1.0907 |  | 0.312 |  |
|  |  |  |  | - |  | Y |  | 8 |  | -0.7292 |  | 0.610 |  | 7.00 |  | -1.1952 |  | 0.271 |  |
|  |  |  |  | - |  | Y |  | 9 |  | -1.0417 |  | 0.691 |  | 7.00 |  | -1.5068 |  | 0.176 |  |
|  |  |  |  | - |  | Y |  | 10 |  | -1.0521 |  | 0.948 |  | 7.00 |  | -1.1099 |  | 0.304 |  |
|  |  | 8 |  | - |  | X |  | 9 |  | 0.3438 |  | 0.431 |  | 7.00 |  | 0.7968 |  | 0.452 |  |
|  |  |  |  | - |  | X |  | 10 |  | 0.0938 |  | 0.196 |  | 7.00 |  | 0.4775 |  | 0.648 |  |
|  |  |  |  | - |  | Y |  | 1 |  | 0.2396 |  | 0.326 |  | 7.00 |  | 0.7341 |  | 0.487 |  |
|  |  |  |  | - |  | Y |  | 2 |  | 0.0208 |  | 0.417 |  | 7.00 |  | 0.0499 |  | 0.962 |  |
|  |  |  |  | - |  | Y |  | 3 |  | -0.8646 |  | 0.323 |  | 7.00 |  | -2.6790 |  | 0.032 |  |
|  |  |  |  | - |  | Y |  | 4 |  | -0.5833 |  | 0.326 |  | 7.00 |  | -1.7883 |  | 0.117 |  |
|  |  |  |  | - |  | Y |  | 5 |  | -1.0937 |  | 0.583 |  | 7.00 |  | -1.8774 |  | 0.103 |  |
|  |  |  |  | - |  | Y |  | 6 |  | -1.1250 |  | 0.533 |  | 7.00 |  | -2.1093 |  | 0.073 |  |
|  |  |  |  | - |  | Y |  | 7 |  | -1.0521 |  | 0.522 |  | 7.00 |  | -2.0161 |  | 0.084 |  |
|  |  |  |  | - |  | Y |  | 8 |  | -1.1562 |  | 0.592 |  | 7.00 |  | -1.9543 |  | 0.092 |  |
|  |  |  |  | - |  | Y |  | 9 |  | -1.4688 |  | 0.595 |  | 7.00 |  | -2.4698 |  | 0.043 |  |
|  |  |  |  | - |  | Y |  | 10 |  | -1.4792 |  | 0.764 |  | 7.00 |  | -1.9368 |  | 0.094 |  |
|  |  | 9 |  | - |  | X |  | 10 |  | -0.2500 |  | 0.487 |  | 7.00 |  | -0.5134 |  | 0.623 |  |
|  |  |  |  | - |  | Y |  | 1 |  | -0.1042 |  | 0.401 |  | 7.00 |  | -0.2601 |  | 0.802 |  |
|  |  |  |  | - |  | Y |  | 2 |  | -0.3229 |  | 0.479 |  | 7.00 |  | -0.6744 |  | 0.522 |  |
|  |  |  |  | - |  | Y |  | 3 |  | -1.2083 |  | 0.449 |  | 7.00 |  | -2.6893 |  | 0.031 |  |
|  |  |  |  | - |  | Y |  | 4 |  | -0.9271 |  | 0.604 |  | 7.00 |  | -1.5341 |  | 0.169 |  |
|  |  |  |  | - |  | Y |  | 5 |  | -1.4375 |  | 0.768 |  | 7.00 |  | -1.8711 |  | 0.104 |  |
|  |  |  |  | - |  | Y |  | 6 |  | -1.4688 |  | 0.720 |  | 7.00 |  | -2.0396 |  | 0.081 |  |
|  |  |  |  | - |  | Y |  | 7 |  | -1.3958 |  | 0.813 |  | 7.00 |  | -1.7176 |  | 0.130 |  |
|  |  |  |  | - |  | Y |  | 8 |  | -1.5000 |  | 0.915 |  | 7.00 |  | -1.6400 |  | 0.145 |  |
|  |  |  |  | - |  | Y |  | 9 |  | -1.8125 |  | 0.841 |  | 7.00 |  | -2.1563 |  | 0.068 |  |
|  |  |  |  | - |  | Y |  | 10 |  | -1.8229 |  | 0.947 |  | 7.00 |  | -1.9251 |  | 0.096 |  |
|  |  | 10 |  | - |  | Y |  | 1 |  | 0.1458 |  | 0.360 |  | 7.00 |  | 0.4050 |  | 0.698 |  |
|  |  |  |  | - |  | Y |  | 2 |  | -0.0729 |  | 0.508 |  | 7.00 |  | -0.1435 |  | 0.890 |  |
|  |  |  |  | - |  | Y |  | 3 |  | -0.9583 |  | 0.340 |  | 7.00 |  | -2.8159 |  | 0.026 |  |
|  |  |  |  | - |  | Y |  | 4 |  | -0.6771 |  | 0.433 |  | 7.00 |  | -1.5642 |  | 0.162 |  |
|  |  |  |  | - |  | Y |  | 5 |  | -1.1875 |  | 0.638 |  | 7.00 |  | -1.8615 |  | 0.105 |  |
|  |  |  |  | - |  | Y |  | 6 |  | -1.2187 |  | 0.568 |  | 7.00 |  | -2.1456 |  | 0.069 |  |
|  |  |  |  | - |  | Y |  | 7 |  | -1.1458 |  | 0.485 |  | 7.00 |  | -2.3634 |  | 0.050 |  |
|  |  |  |  | - |  | Y |  | 8 |  | -1.2500 |  | 0.534 |  | 7.00 |  | -2.3406 |  | 0.052 |  |
|  |  |  |  | - |  | Y |  | 9 |  | -1.5625 |  | 0.551 |  | 7.00 |  | -2.8339 |  | 0.025 |  |
|  |  |  |  | - |  | Y |  | 10 |  | -1.5729 |  | 0.875 |  | 7.00 |  | -1.7966 |  | 0.115 |  |
| Y |  | 1 |  | - |  | Y |  | 2 |  | -0.2188 |  | 0.246 |  | 7.00 |  | -0.8893 |  | 0.403 |  |
|  |  |  |  | - |  | Y |  | 3 |  | -1.1042 |  | 0.225 |  | 7.00 |  | -4.9119 |  | 0.002 |  |
|  |  |  |  | - |  | Y |  | 4 |  | -0.8229 |  | 0.351 |  | 7.00 |  | -2.3429 |  | 0.052 |  |
|  |  |  |  | - |  | Y |  | 5 |  | -1.3333 |  | 0.449 |  | 7.00 |  | -2.9693 |  | 0.021 |  |
|  |  |  |  | - |  | Y |  | 6 |  | -1.3646 |  | 0.488 |  | 7.00 |  | -2.7981 |  | 0.027 |  |
|  |  |  |  | - |  | Y |  | 7 |  | -1.2917 |  | 0.555 |  | 7.00 |  | -2.3264 |  | 0.053 |  |
|  |  |  |  | - |  | Y |  | 8 |  | -1.3958 |  | 0.683 |  | 7.00 |  | -2.0427 |  | 0.080 |  |
|  |  |  |  | - |  | Y |  | 9 |  | -1.7083 |  | 0.650 |  | 7.00 |  | -2.6286 |  | 0.034 |  |
|  |  |  |  | - |  | Y |  | 10 |  | -1.7188 |  | 0.768 |  | 7.00 |  | -2.2385 |  | 0.060 |  |
|  |  | 2 |  | - |  | Y |  | 3 |  | -0.8854 |  | 0.378 |  | 7.00 |  | -2.3427 |  | 0.052 |  |
|  |  |  |  | - |  | Y |  | 4 |  | -0.6042 |  | 0.358 |  | 7.00 |  | -1.6876 |  | 0.135 |  |
|  |  |  |  | - |  | Y |  | 5 |  | -1.1146 |  | 0.497 |  | 7.00 |  | -2.2415 |  | 0.060 |  |
|  |  |  |  | - |  | Y |  | 6 |  | -1.1458 |  | 0.561 |  | 7.00 |  | -2.0443 |  | 0.080 |  |
|  |  |  |  | - |  | Y |  | 7 |  | -1.0729 |  | 0.627 |  | 7.00 |  | -1.7115 |  | 0.131 |  |
|  |  |  |  | - |  | Y |  | 8 |  | -1.1771 |  | 0.756 |  | 7.00 |  | -1.5570 |  | 0.163 |  |
|  |  |  |  | - |  | Y |  | 9 |  | -1.4896 |  | 0.771 |  | 7.00 |  | -1.9318 |  | 0.095 |  |
|  |  |  |  | - |  | Y |  | 10 |  | -1.5000 |  | 0.796 |  | 7.00 |  | -1.8840 |  | 0.102 |  |
|  |  | 3 |  | - |  | Y |  | 4 |  | 0.2813 |  | 0.292 |  | 7.00 |  | 0.9630 |  | 0.368 |  |
|  |  |  |  | - |  | Y |  | 5 |  | -0.2292 |  | 0.386 |  | 7.00 |  | -0.5937 |  | 0.571 |  |
|  |  |  |  | - |  | Y |  | 6 |  | -0.2604 |  | 0.329 |  | 7.00 |  | -0.7924 |  | 0.454 |  |
|  |  |  |  | - |  | Y |  | 7 |  | -0.1875 |  | 0.409 |  | 7.00 |  | -0.4583 |  | 0.661 |  |
|  |  |  |  | - |  | Y |  | 8 |  | -0.2917 |  | 0.545 |  | 7.00 |  | -0.5355 |  | 0.609 |  |
|  |  |  |  | - |  | Y |  | 9 |  | -0.6042 |  | 0.460 |  | 7.00 |  | -1.3145 |  | 0.230 |  |
|  |  |  |  | - |  | Y |  | 10 |  | -0.6146 |  | 0.668 |  | 7.00 |  | -0.9196 |  | 0.388 |  |
|  |  | 4 |  | - |  | Y |  | 5 |  | -0.5104 |  | 0.349 |  | 7.00 |  | -1.4605 |  | 0.188 |  |
|  |  |  |  | - |  | Y |  | 6 |  | -0.5417 |  | 0.367 |  | 7.00 |  | -1.4760 |  | 0.183 |  |
|  |  |  |  | - |  | Y |  | 7 |  | -0.4688 |  | 0.405 |  | 7.00 |  | -1.1578 |  | 0.285 |  |
|  |  |  |  | - |  | Y |  | 8 |  | -0.5729 |  | 0.497 |  | 7.00 |  | -1.1530 |  | 0.287 |  |
|  |  |  |  | - |  | Y |  | 9 |  | -0.8854 |  | 0.528 |  | 7.00 |  | -1.6755 |  | 0.138 |  |
|  |  |  |  | - |  | Y |  | 10 |  | -0.8958 |  | 0.594 |  | 7.00 |  | -1.5081 |  | 0.175 |  |
|  |  | 5 |  | - |  | Y |  | 6 |  | -0.0313 |  | 0.251 |  | 7.00 |  | -0.1245 |  | 0.904 |  |
|  |  |  |  | - |  | Y |  | 7 |  | 0.0417 |  | 0.446 |  | 7.00 |  | 0.0934 |  | 0.928 |  |
|  |  |  |  | - |  | Y |  | 8 |  | -0.0625 |  | 0.626 |  | 7.00 |  | -0.0999 |  | 0.923 |  |
|  |  |  |  | - |  | Y |  | 9 |  | -0.3750 |  | 0.515 |  | 7.00 |  | -0.7279 |  | 0.490 |  |
|  |  |  |  | - |  | Y |  | 10 |  | -0.3854 |  | 0.487 |  | 7.00 |  | -0.7915 |  | 0.455 |  |
|  |  | 6 |  | - |  | Y |  | 7 |  | 0.0729 |  | 0.317 |  | 7.00 |  | 0.2302 |  | 0.824 |  |
|  |  |  |  | - |  | Y |  | 8 |  | -0.0313 |  | 0.527 |  | 7.00 |  | -0.0593 |  | 0.954 |  |
|  |  |  |  | - |  | Y |  | 9 |  | -0.3438 |  | 0.317 |  | 7.00 |  | -1.0854 |  | 0.314 |  |
|  |  |  |  | - |  | Y |  | 10 |  | -0.3542 |  | 0.513 |  | 7.00 |  | -0.6898 |  | 0.513 |  |
|  |  | 7 |  | - |  | Y |  | 8 |  | -0.1042 |  | 0.250 |  | 7.00 |  | -0.4169 |  | 0.689 |  |
|  |  |  |  | - |  | Y |  | 9 |  | -0.4167 |  | 0.245 |  | 7.00 |  | -1.6973 |  | 0.133 |  |
|  |  |  |  | - |  | Y |  | 10 |  | -0.4271 |  | 0.729 |  | 7.00 |  | -0.5858 |  | 0.576 |  |
|  |  | 8 |  | - |  | Y |  | 9 |  | -0.3125 |  | 0.374 |  | 7.00 |  | -0.8350 |  | 0.431 |  |
|  |  |  |  | - |  | Y |  | 10 |  | -0.3229 |  | 0.863 |  | 7.00 |  | -0.3740 |  | 0.719 |  |
|  |  | 9 |  | - |  | Y |  | 10 |  | -0.0104 |  | 0.697 |  | 7.00 |  | -0.0150 |  | 0.988 |  |
|  | | | | | | | | | | | | | | | | | | | |

**Compound phase (excluding X and Y trials)**

| Within Subjects Effects | | | | | | | | | | | | | | | |
| --- | --- | --- | --- | --- | --- | --- | --- | --- | --- | --- | --- | --- | --- | --- | --- |
|  |  |  |  |  |  |  |  |  |  |  |  |  |  |  |  |
|  | | **Sum of Squares** | | **df** | | **Mean Square** | | **F** | | **p** | | **η²** | | **η²_p_** | |
| block |  | 8.10 |  | 9 |  | 0.899 |  | 0.814 |  | 0.605 |  | 0.008 |  | 0.104 |  |
| Residual |  | 69.64 |  | 63 |  | 1.105 |  |  |  |  |  |  |  |  |  |
| stimulus |  | 417.95 |  | 3 |  | 139.317 |  | 17.106 |  | < .001 |  | 0.395 |  | 0.710 |  |
| Residual |  | 171.04 |  | 21 |  | 8.145 |  |  |  |  |  |  |  |  |  |
| block ✻ stimulus |  | 35.84 |  | 27 |  | 1.327 |  | 2.544 |  | < .001 |  | 0.034 |  | 0.267 |  |
| Residual |  | 98.62 |  | 189 |  | 0.522 |  |  |  |  |  |  |  |  |  |
| Note. Type 3 Sums of Squares | | | | | | | | | | | | | | | |
|  | | | | | | | | | | | | | | | |

| Post Hoc Comparisons - block ✻ stimulus | | | | | | | | | | | | | | | | | | | |
| --- | --- | --- | --- | --- | --- | --- | --- | --- | --- | --- | --- | --- | --- | --- | --- | --- | --- | --- | --- |
| **Comparison** | | | | | | | | | |  | | | | | | | | | |
| **block** | | **stimulus** | |  | | **block** | | **stimulus** | | **Mean Difference** | | **SE** | | **df** | | **t** | | **p** | |
| 1 |  | A |  | - |  | 1 |  | AX |  | -1.13542 |  | 0.2461 |  | 7.00 |  | -4.6140 |  | 0.002 |  |
|  |  |  |  | - |  | 1 |  | B |  | 1.35417 |  | 0.2868 |  | 7.00 |  | 4.7209 |  | 0.002 |  |
|  |  |  |  | - |  | 1 |  | BY |  | 0.38194 |  | 0.2762 |  | 7.00 |  | 1.3827 |  | 0.209 |  |
|  |  |  |  | - |  | 2 |  | A |  | -0.41667 |  | 0.3429 |  | 7.00 |  | -1.2152 |  | 0.264 |  |
|  |  |  |  | - |  | 2 |  | AX |  | -0.93403 |  | 0.3671 |  | 7.00 |  | -2.5444 |  | 0.038 |  |
|  |  |  |  | - |  | 2 |  | B |  | 1.46875 |  | 0.4287 |  | 7.00 |  | 3.4262 |  | 0.011 |  |
|  |  |  |  | - |  | 2 |  | BY |  | 0.55208 |  | 0.3850 |  | 7.00 |  | 1.4340 |  | 0.195 |  |
|  |  |  |  | - |  | 3 |  | A |  | -0.78125 |  | 0.3432 |  | 7.00 |  | -2.2763 |  | 0.057 |  |
|  |  |  |  | - |  | 3 |  | AX |  | -1.18750 |  | 0.4467 |  | 7.00 |  | -2.6586 |  | 0.033 |  |
|  |  |  |  | - |  | 3 |  | B |  | 1.45833 |  | 0.4137 |  | 7.00 |  | 3.5253 |  | 0.010 |  |
|  |  |  |  | - |  | 3 |  | BY |  | 0.22222 |  | 0.4563 |  | 7.00 |  | 0.4870 |  | 0.641 |  |
|  |  |  |  | - |  | 4 |  | A |  | -0.75000 |  | 0.5079 |  | 7.00 |  | -1.4767 |  | 0.183 |  |
|  |  |  |  | - |  | 4 |  | AX |  | -0.76736 |  | 0.4970 |  | 7.00 |  | -1.5441 |  | 0.166 |  |
|  |  |  |  | - |  | 4 |  | B |  | 1.88542 |  | 0.3965 |  | 7.00 |  | 4.7549 |  | 0.002 |  |
|  |  |  |  | - |  | 4 |  | BY |  | 0.35417 |  | 0.4871 |  | 7.00 |  | 0.7271 |  | 0.491 |  |
|  |  |  |  | - |  | 5 |  | A |  | -0.37500 |  | 0.5414 |  | 7.00 |  | -0.6926 |  | 0.511 |  |
|  |  |  |  | - |  | 5 |  | AX |  | -0.81944 |  | 0.5050 |  | 7.00 |  | -1.6225 |  | 0.149 |  |
|  |  |  |  | - |  | 5 |  | B |  | 1.57292 |  | 0.3372 |  | 7.00 |  | 4.6647 |  | 0.002 |  |
|  |  |  |  | - |  | 5 |  | BY |  | 0.80556 |  | 0.3676 |  | 7.00 |  | 2.1915 |  | 0.065 |  |
|  |  |  |  | - |  | 6 |  | A |  | -0.84375 |  | 0.5604 |  | 7.00 |  | -1.5055 |  | 0.176 |  |
|  |  |  |  | - |  | 6 |  | AX |  | -1.04875 |  | 0.5783 |  | 7.00 |  | -1.8136 |  | 0.113 |  |
|  |  |  |  | - |  | 6 |  | B |  | 1.82292 |  | 0.4053 |  | 7.00 |  | 4.4973 |  | 0.003 |  |
|  |  |  |  | - |  | 6 |  | BY |  | 1.00694 |  | 0.3209 |  | 7.00 |  | 3.1375 |  | 0.016 |  |
|  |  |  |  | - |  | 7 |  | A |  | -0.66667 |  | 0.4730 |  | 7.00 |  | -1.4095 |  | 0.202 |  |
|  |  |  |  | - |  | 7 |  | AX |  | -0.75694 |  | 0.5164 |  | 7.00 |  | -1.4657 |  | 0.186 |  |
|  |  |  |  | - |  | 7 |  | B |  | 2.00000 |  | 0.4637 |  | 7.00 |  | 4.3130 |  | 0.004 |  |
|  |  |  |  | - |  | 7 |  | BY |  | 1.28125 |  | 0.4183 |  | 7.00 |  | 3.0628 |  | 0.018 |  |
|  |  |  |  | - |  | 8 |  | A |  | -1.27083 |  | 0.8017 |  | 7.00 |  | -1.5851 |  | 0.157 |  |
|  |  |  |  | - |  | 8 |  | AX |  | -1.06944 |  | 0.7243 |  | 7.00 |  | -1.4764 |  | 0.183 |  |
|  |  |  |  | - |  | 8 |  | B |  | 2.08333 |  | 0.5113 |  | 7.00 |  | 4.0747 |  | 0.005 |  |
|  |  |  |  | - |  | 8 |  | BY |  | 1.46181 |  | 0.4166 |  | 7.00 |  | 3.5092 |  | 0.010 |  |
|  |  |  |  | - |  | 9 |  | A |  | -1.13542 |  | 0.8992 |  | 7.00 |  | -1.2627 |  | 0.247 |  |
|  |  |  |  | - |  | 9 |  | AX |  | -0.96181 |  | 0.6879 |  | 7.00 |  | -1.3981 |  | 0.205 |  |
|  |  |  |  | - |  | 9 |  | B |  | 2.08333 |  | 0.5044 |  | 7.00 |  | 4.1300 |  | 0.004 |  |
|  |  |  |  | - |  | 9 |  | BY |  | 1.77778 |  | 0.5111 |  | 7.00 |  | 3.4782 |  | 0.010 |  |
|  |  |  |  | - |  | 10 |  | A |  | -1.17708 |  | 0.7484 |  | 7.00 |  | -1.5728 |  | 0.160 |  |
|  |  |  |  | - |  | 10 |  | AX |  | -0.75000 |  | 0.5728 |  | 7.00 |  | -1.3093 |  | 0.232 |  |
|  |  |  |  | - |  | 10 |  | B |  | 1.97917 |  | 0.4838 |  | 7.00 |  | 4.0909 |  | 0.005 |  |
|  |  |  |  | - |  | 10 |  | BY |  | 1.76389 |  | 0.4751 |  | 7.00 |  | 3.7124 |  | 0.008 |  |
|  |  | AX |  | - |  | 1 |  | B |  | 2.48958 |  | 0.4261 |  | 7.00 |  | 5.8430 |  | < .001 |  |
|  |  |  |  | - |  | 1 |  | BY |  | 1.51736 |  | 0.4098 |  | 7.00 |  | 3.7027 |  | 0.008 |  |
|  |  |  |  | - |  | 2 |  | A |  | 0.71875 |  | 0.3072 |  | 7.00 |  | 2.3397 |  | 0.052 |  |
|  |  |  |  | - |  | 2 |  | AX |  | 0.20139 |  | 0.3274 |  | 7.00 |  | 0.6152 |  | 0.558 |  |
|  |  |  |  | - |  | 2 |  | B |  | 2.60417 |  | 0.5723 |  | 7.00 |  | 4.5503 |  | 0.003 |  |
|  |  |  |  | - |  | 2 |  | BY |  | 1.68750 |  | 0.5011 |  | 7.00 |  | 3.3676 |  | 0.012 |  |
|  |  |  |  | - |  | 3 |  | A |  | 0.35417 |  | 0.4919 |  | 7.00 |  | 0.7200 |  | 0.495 |  |
|  |  |  |  | - |  | 3 |  | AX |  | -0.05208 |  | 0.4843 |  | 7.00 |  | -0.1075 |  | 0.917 |  |
|  |  |  |  | - |  | 3 |  | B |  | 2.59375 |  | 0.5999 |  | 7.00 |  | 4.3237 |  | 0.003 |  |
|  |  |  |  | - |  | 3 |  | BY |  | 1.35764 |  | 0.5572 |  | 7.00 |  | 2.4365 |  | 0.045 |  |
|  |  |  |  | - |  | 4 |  | A |  | 0.38542 |  | 0.5801 |  | 7.00 |  | 0.6644 |  | 0.528 |  |
|  |  |  |  | - |  | 4 |  | AX |  | 0.36806 |  | 0.5606 |  | 7.00 |  | 0.6565 |  | 0.532 |  |
|  |  |  |  | - |  | 4 |  | B |  | 3.02083 |  | 0.5541 |  | 7.00 |  | 5.4517 |  | < .001 |  |
|  |  |  |  | - |  | 4 |  | BY |  | 1.48958 |  | 0.6215 |  | 7.00 |  | 2.3966 |  | 0.048 |  |
|  |  |  |  | - |  | 5 |  | A |  | 0.76042 |  | 0.5946 |  | 7.00 |  | 1.2788 |  | 0.242 |  |
|  |  |  |  | - |  | 5 |  | AX |  | 0.31597 |  | 0.5756 |  | 7.00 |  | 0.5490 |  | 0.600 |  |
|  |  |  |  | - |  | 5 |  | B |  | 2.70833 |  | 0.4656 |  | 7.00 |  | 5.8175 |  | < .001 |  |
|  |  |  |  | - |  | 5 |  | BY |  | 1.94097 |  | 0.5041 |  | 7.00 |  | 3.8506 |  | 0.006 |  |
|  |  |  |  | - |  | 6 |  | A |  | 0.29167 |  | 0.5952 |  | 7.00 |  | 0.4900 |  | 0.639 |  |
|  |  |  |  | - |  | 6 |  | AX |  | 0.08667 |  | 0.6361 |  | 7.00 |  | 0.1363 |  | 0.895 |  |
|  |  |  |  | - |  | 6 |  | B |  | 2.95833 |  | 0.5775 |  | 7.00 |  | 5.1223 |  | 0.001 |  |
|  |  |  |  | - |  | 6 |  | BY |  | 2.14236 |  | 0.4630 |  | 7.00 |  | 4.6267 |  | 0.002 |  |
|  |  |  |  | - |  | 7 |  | A |  | 0.46875 |  | 0.5360 |  | 7.00 |  | 0.8745 |  | 0.411 |  |
|  |  |  |  | - |  | 7 |  | AX |  | 0.37847 |  | 0.5824 |  | 7.00 |  | 0.6498 |  | 0.537 |  |
|  |  |  |  | - |  | 7 |  | B |  | 3.13542 |  | 0.6326 |  | 7.00 |  | 4.9565 |  | 0.002 |  |
|  |  |  |  | - |  | 7 |  | BY |  | 2.41667 |  | 0.5617 |  | 7.00 |  | 4.3026 |  | 0.004 |  |
|  |  |  |  | - |  | 8 |  | A |  | -0.13542 |  | 0.8357 |  | 7.00 |  | -0.1620 |  | 0.876 |  |
|  |  |  |  | - |  | 8 |  | AX |  | 0.06597 |  | 0.7784 |  | 7.00 |  | 0.0848 |  | 0.935 |  |
|  |  |  |  | - |  | 8 |  | B |  | 3.21875 |  | 0.6511 |  | 7.00 |  | 4.9437 |  | 0.002 |  |
|  |  |  |  | - |  | 8 |  | BY |  | 2.59722 |  | 0.5867 |  | 7.00 |  | 4.4268 |  | 0.003 |  |
|  |  |  |  | - |  | 9 |  | A |  | 8.88e-16 |  | 0.9584 |  | 7.00 |  | 9.27e-16 |  | 1.000 |  |
|  |  |  |  | - |  | 9 |  | AX |  | 0.17361 |  | 0.7595 |  | 7.00 |  | 0.2286 |  | 0.826 |  |
|  |  |  |  | - |  | 9 |  | B |  | 3.21875 |  | 0.6956 |  | 7.00 |  | 4.6274 |  | 0.002 |  |
|  |  |  |  | - |  | 9 |  | BY |  | 2.91319 |  | 0.6622 |  | 7.00 |  | 4.3996 |  | 0.003 |  |
|  |  |  |  | - |  | 10 |  | A |  | -0.04167 |  | 0.8651 |  | 7.00 |  | -0.0482 |  | 0.963 |  |
|  |  |  |  | - |  | 10 |  | AX |  | 0.38542 |  | 0.7065 |  | 7.00 |  | 0.5456 |  | 0.602 |  |
|  |  |  |  | - |  | 10 |  | B |  | 3.11458 |  | 0.6466 |  | 7.00 |  | 4.8167 |  | 0.002 |  |
|  |  |  |  | - |  | 10 |  | BY |  | 2.89931 |  | 0.6265 |  | 7.00 |  | 4.6280 |  | 0.002 |  |
|  |  | B |  | - |  | 1 |  | BY |  | -0.97222 |  | 0.0880 |  | 7.00 |  | -11.0483 |  | < .001 |  |
|  |  |  |  | - |  | 2 |  | A |  | -1.77083 |  | 0.3510 |  | 7.00 |  | -5.0451 |  | 0.001 |  |
|  |  |  |  | - |  | 2 |  | AX |  | -2.28819 |  | 0.4081 |  | 7.00 |  | -5.6063 |  | < .001 |  |
|  |  |  |  | - |  | 2 |  | B |  | 0.11458 |  | 0.1767 |  | 7.00 |  | 0.6483 |  | 0.537 |  |
|  |  |  |  | - |  | 2 |  | BY |  | -0.80208 |  | 0.1443 |  | 7.00 |  | -5.5591 |  | < .001 |  |
|  |  |  |  | - |  | 3 |  | A |  | -2.13542 |  | 0.2874 |  | 7.00 |  | -7.4312 |  | < .001 |  |
|  |  |  |  | - |  | 3 |  | AX |  | -2.54167 |  | 0.3772 |  | 7.00 |  | -6.7383 |  | < .001 |  |
|  |  |  |  | - |  | 3 |  | B |  | 0.10417 |  | 0.3435 |  | 7.00 |  | 0.3032 |  | 0.771 |  |
|  |  |  |  | - |  | 3 |  | BY |  | -1.13194 |  | 0.2336 |  | 7.00 |  | -4.8462 |  | 0.002 |  |
|  |  |  |  | - |  | 4 |  | A |  | -2.10417 |  | 0.4005 |  | 7.00 |  | -5.2538 |  | 0.001 |  |
|  |  |  |  | - |  | 4 |  | AX |  | -2.12153 |  | 0.3685 |  | 7.00 |  | -5.7576 |  | < .001 |  |
|  |  |  |  | - |  | 4 |  | B |  | 0.53125 |  | 0.2539 |  | 7.00 |  | 2.0923 |  | 0.075 |  |
|  |  |  |  | - |  | 4 |  | BY |  | -1.00000 |  | 0.2223 |  | 7.00 |  | -4.4987 |  | 0.003 |  |
|  |  |  |  | - |  | 5 |  | A |  | -1.72917 |  | 0.3332 |  | 7.00 |  | -5.1889 |  | 0.001 |  |
|  |  |  |  | - |  | 5 |  | AX |  | -2.17361 |  | 0.3810 |  | 7.00 |  | -5.7055 |  | < .001 |  |
|  |  |  |  | - |  | 5 |  | B |  | 0.21875 |  | 0.2383 |  | 7.00 |  | 0.9180 |  | 0.389 |  |
|  |  |  |  | - |  | 5 |  | BY |  | -0.54861 |  | 0.1428 |  | 7.00 |  | -3.8424 |  | 0.006 |  |
|  |  |  |  | - |  | 6 |  | A |  | -2.19792 |  | 0.5088 |  | 7.00 |  | -4.3195 |  | 0.003 |  |
|  |  |  |  | - |  | 6 |  | AX |  | -2.40292 |  | 0.5382 |  | 7.00 |  | -4.4644 |  | 0.003 |  |
|  |  |  |  | - |  | 6 |  | B |  | 0.46875 |  | 0.2495 |  | 7.00 |  | 1.8790 |  | 0.102 |  |
|  |  |  |  | - |  | 6 |  | BY |  | -0.34722 |  | 0.1090 |  | 7.00 |  | -3.1860 |  | 0.015 |  |
|  |  |  |  | - |  | 7 |  | A |  | -2.02083 |  | 0.4615 |  | 7.00 |  | -4.3788 |  | 0.003 |  |
|  |  |  |  | - |  | 7 |  | AX |  | -2.11111 |  | 0.5087 |  | 7.00 |  | -4.1501 |  | 0.004 |  |
|  |  |  |  | - |  | 7 |  | B |  | 0.64583 |  | 0.3549 |  | 7.00 |  | 1.8199 |  | 0.112 |  |
|  |  |  |  | - |  | 7 |  | BY |  | -0.07292 |  | 0.2380 |  | 7.00 |  | -0.3063 |  | 0.768 |  |
|  |  |  |  | - |  | 8 |  | A |  | -2.62500 |  | 0.8639 |  | 7.00 |  | -3.0386 |  | 0.019 |  |
|  |  |  |  | - |  | 8 |  | AX |  | -2.42361 |  | 0.7489 |  | 7.00 |  | -3.2361 |  | 0.014 |  |
|  |  |  |  | - |  | 8 |  | B |  | 0.72917 |  | 0.3406 |  | 7.00 |  | 2.1408 |  | 0.070 |  |
|  |  |  |  | - |  | 8 |  | BY |  | 0.10764 |  | 0.2086 |  | 7.00 |  | 0.5159 |  | 0.622 |  |
|  |  |  |  | - |  | 9 |  | A |  | -2.48958 |  | 1.0012 |  | 7.00 |  | -2.4867 |  | 0.042 |  |
|  |  |  |  | - |  | 9 |  | AX |  | -2.31597 |  | 0.7489 |  | 7.00 |  | -3.0927 |  | 0.018 |  |
|  |  |  |  | - |  | 9 |  | B |  | 0.72917 |  | 0.4063 |  | 7.00 |  | 1.7945 |  | 0.116 |  |
|  |  |  |  | - |  | 9 |  | BY |  | 0.42361 |  | 0.3874 |  | 7.00 |  | 1.0935 |  | 0.310 |  |
|  |  |  |  | - |  | 10 |  | A |  | -2.53125 |  | 0.7862 |  | 7.00 |  | -3.2198 |  | 0.015 |  |
|  |  |  |  | - |  | 10 |  | AX |  | -2.10417 |  | 0.5729 |  | 7.00 |  | -3.6731 |  | 0.008 |  |
|  |  |  |  | - |  | 10 |  | B |  | 0.62500 |  | 0.3254 |  | 7.00 |  | 1.9206 |  | 0.096 |  |
|  |  |  |  | - |  | 10 |  | BY |  | 0.40972 |  | 0.3121 |  | 7.00 |  | 1.3130 |  | 0.231 |  |
|  |  | BY |  | - |  | 2 |  | A |  | -0.79861 |  | 0.3595 |  | 7.00 |  | -2.2217 |  | 0.062 |  |
|  |  |  |  | - |  | 2 |  | AX |  | -1.31597 |  | 0.4052 |  | 7.00 |  | -3.2477 |  | 0.014 |  |
|  |  |  |  | - |  | 2 |  | B |  | 1.08681 |  | 0.2098 |  | 7.00 |  | 5.1799 |  | 0.001 |  |
|  |  |  |  | - |  | 2 |  | BY |  | 0.17014 |  | 0.1381 |  | 7.00 |  | 1.2317 |  | 0.258 |  |
|  |  |  |  | - |  | 3 |  | A |  | -1.16319 |  | 0.3246 |  | 7.00 |  | -3.5835 |  | 0.009 |  |
|  |  |  |  | - |  | 3 |  | AX |  | -1.56944 |  | 0.3948 |  | 7.00 |  | -3.9757 |  | 0.005 |  |
|  |  |  |  | - |  | 3 |  | B |  | 1.07639 |  | 0.3850 |  | 7.00 |  | 2.7958 |  | 0.027 |  |
|  |  |  |  | - |  | 3 |  | BY |  | -0.15972 |  | 0.2341 |  | 7.00 |  | -0.6823 |  | 0.517 |  |
|  |  |  |  | - |  | 4 |  | A |  | -1.13194 |  | 0.4332 |  | 7.00 |  | -2.6130 |  | 0.035 |  |
|  |  |  |  | - |  | 4 |  | AX |  | -1.14931 |  | 0.4140 |  | 7.00 |  | -2.7760 |  | 0.027 |  |
|  |  |  |  | - |  | 4 |  | B |  | 1.50347 |  | 0.2791 |  | 7.00 |  | 5.3862 |  | 0.001 |  |
|  |  |  |  | - |  | 4 |  | BY |  | -0.02778 |  | 0.2449 |  | 7.00 |  | -0.1134 |  | 0.913 |  |
|  |  |  |  | - |  | 5 |  | A |  | -0.75694 |  | 0.3850 |  | 7.00 |  | -1.9661 |  | 0.090 |  |
|  |  |  |  | - |  | 5 |  | AX |  | -1.20139 |  | 0.4189 |  | 7.00 |  | -2.8677 |  | 0.024 |  |
|  |  |  |  | - |  | 5 |  | B |  | 1.19097 |  | 0.2721 |  | 7.00 |  | 4.3772 |  | 0.003 |  |
|  |  |  |  | - |  | 5 |  | BY |  | 0.42361 |  | 0.1926 |  | 7.00 |  | 2.1989 |  | 0.064 |  |
|  |  |  |  | - |  | 6 |  | A |  | -1.22569 |  | 0.5508 |  | 7.00 |  | -2.2254 |  | 0.061 |  |
|  |  |  |  | - |  | 6 |  | AX |  | -1.43069 |  | 0.5663 |  | 7.00 |  | -2.5263 |  | 0.039 |  |
|  |  |  |  | - |  | 6 |  | B |  | 1.44097 |  | 0.2867 |  | 7.00 |  | 5.0255 |  | 0.002 |  |
|  |  |  |  | - |  | 6 |  | BY |  | 0.62500 |  | 0.1514 |  | 7.00 |  | 4.1276 |  | 0.004 |  |
|  |  |  |  | - |  | 7 |  | A |  | -1.04861 |  | 0.4968 |  | 7.00 |  | -2.1106 |  | 0.073 |  |
|  |  |  |  | - |  | 7 |  | AX |  | -1.13889 |  | 0.5312 |  | 7.00 |  | -2.1442 |  | 0.069 |  |
|  |  |  |  | - |  | 7 |  | B |  | 1.61806 |  | 0.3820 |  | 7.00 |  | 4.2360 |  | 0.004 |  |
|  |  |  |  | - |  | 7 |  | BY |  | 0.89931 |  | 0.2367 |  | 7.00 |  | 3.7986 |  | 0.007 |  |
|  |  |  |  | - |  | 8 |  | A |  | -1.65278 |  | 0.8844 |  | 7.00 |  | -1.8689 |  | 0.104 |  |
|  |  |  |  | - |  | 8 |  | AX |  | -1.45139 |  | 0.7729 |  | 7.00 |  | -1.8778 |  | 0.102 |  |
|  |  |  |  | - |  | 8 |  | B |  | 1.70139 |  | 0.3691 |  | 7.00 |  | 4.6095 |  | 0.002 |  |
|  |  |  |  | - |  | 8 |  | BY |  | 1.07986 |  | 0.2154 |  | 7.00 |  | 5.0121 |  | 0.002 |  |
|  |  |  |  | - |  | 9 |  | A |  | -1.51736 |  | 1.0132 |  | 7.00 |  | -1.4976 |  | 0.178 |  |
|  |  |  |  | - |  | 9 |  | AX |  | -1.34375 |  | 0.7639 |  | 7.00 |  | -1.7591 |  | 0.122 |  |
|  |  |  |  | - |  | 9 |  | B |  | 1.70139 |  | 0.4113 |  | 7.00 |  | 4.1370 |  | 0.004 |  |
|  |  |  |  | - |  | 9 |  | BY |  | 1.39583 |  | 0.3878 |  | 7.00 |  | 3.5992 |  | 0.009 |  |
|  |  |  |  | - |  | 10 |  | A |  | -1.55903 |  | 0.8007 |  | 7.00 |  | -1.9471 |  | 0.093 |  |
|  |  |  |  | - |  | 10 |  | AX |  | -1.13194 |  | 0.5972 |  | 7.00 |  | -1.8955 |  | 0.100 |  |
|  |  |  |  | - |  | 10 |  | B |  | 1.59722 |  | 0.3369 |  | 7.00 |  | 4.7414 |  | 0.002 |  |
|  |  |  |  | - |  | 10 |  | BY |  | 1.38194 |  | 0.3196 |  | 7.00 |  | 4.3245 |  | 0.003 |  |
| 2 |  | A |  | - |  | 2 |  | AX |  | -0.51736 |  | 0.2882 |  | 7.00 |  | -1.7949 |  | 0.116 |  |
|  |  |  |  | - |  | 2 |  | B |  | 1.88542 |  | 0.5081 |  | 7.00 |  | 3.7107 |  | 0.008 |  |
|  |  |  |  | - |  | 2 |  | BY |  | 0.96875 |  | 0.3975 |  | 7.00 |  | 2.4374 |  | 0.045 |  |
|  |  |  |  | - |  | 3 |  | A |  | -0.36458 |  | 0.3570 |  | 7.00 |  | -1.0212 |  | 0.341 |  |
|  |  |  |  | - |  | 3 |  | AX |  | -0.77083 |  | 0.3069 |  | 7.00 |  | -2.5117 |  | 0.040 |  |
|  |  |  |  | - |  | 3 |  | B |  | 1.87500 |  | 0.6301 |  | 7.00 |  | 2.9755 |  | 0.021 |  |
|  |  |  |  | - |  | 3 |  | BY |  | 0.63889 |  | 0.4343 |  | 7.00 |  | 1.4710 |  | 0.185 |  |
|  |  |  |  | - |  | 4 |  | A |  | -0.33333 |  | 0.3629 |  | 7.00 |  | -0.9185 |  | 0.389 |  |
|  |  |  |  | - |  | 4 |  | AX |  | -0.35069 |  | 0.3387 |  | 7.00 |  | -1.0355 |  | 0.335 |  |
|  |  |  |  | - |  | 4 |  | B |  | 2.30208 |  | 0.5625 |  | 7.00 |  | 4.0923 |  | 0.005 |  |
|  |  |  |  | - |  | 4 |  | BY |  | 0.77083 |  | 0.5293 |  | 7.00 |  | 1.4563 |  | 0.189 |  |
|  |  |  |  | - |  | 5 |  | A |  | 0.04167 |  | 0.4104 |  | 7.00 |  | 0.1015 |  | 0.922 |  |
|  |  |  |  | - |  | 5 |  | AX |  | -0.40278 |  | 0.3725 |  | 7.00 |  | -1.0813 |  | 0.315 |  |
|  |  |  |  | - |  | 5 |  | B |  | 1.98958 |  | 0.5085 |  | 7.00 |  | 3.9129 |  | 0.006 |  |
|  |  |  |  | - |  | 5 |  | BY |  | 1.22222 |  | 0.4153 |  | 7.00 |  | 2.9429 |  | 0.022 |  |
|  |  |  |  | - |  | 6 |  | A |  | -0.42708 |  | 0.4026 |  | 7.00 |  | -1.0609 |  | 0.324 |  |
|  |  |  |  | - |  | 6 |  | AX |  | -0.63208 |  | 0.4365 |  | 7.00 |  | -1.4480 |  | 0.191 |  |
|  |  |  |  | - |  | 6 |  | B |  | 2.23958 |  | 0.5757 |  | 7.00 |  | 3.8900 |  | 0.006 |  |
|  |  |  |  | - |  | 6 |  | BY |  | 1.42361 |  | 0.3835 |  | 7.00 |  | 3.7122 |  | 0.008 |  |
|  |  |  |  | - |  | 7 |  | A |  | -0.25000 |  | 0.3883 |  | 7.00 |  | -0.6438 |  | 0.540 |  |
|  |  |  |  | - |  | 7 |  | AX |  | -0.34028 |  | 0.4036 |  | 7.00 |  | -0.8431 |  | 0.427 |  |
|  |  |  |  | - |  | 7 |  | B |  | 2.41667 |  | 0.6726 |  | 7.00 |  | 3.5931 |  | 0.009 |  |
|  |  |  |  | - |  | 7 |  | BY |  | 1.69792 |  | 0.5642 |  | 7.00 |  | 3.0097 |  | 0.020 |  |
|  |  |  |  | - |  | 8 |  | A |  | -0.85417 |  | 0.7172 |  | 7.00 |  | -1.1910 |  | 0.272 |  |
|  |  |  |  | - |  | 8 |  | AX |  | -0.65278 |  | 0.6167 |  | 7.00 |  | -1.0585 |  | 0.325 |  |
|  |  |  |  | - |  | 8 |  | B |  | 2.50000 |  | 0.6528 |  | 7.00 |  | 3.8299 |  | 0.006 |  |
|  |  |  |  | - |  | 8 |  | BY |  | 1.87847 |  | 0.5566 |  | 7.00 |  | 3.3751 |  | 0.012 |  |
|  |  |  |  | - |  | 9 |  | A |  | -0.71875 |  | 0.9231 |  | 7.00 |  | -0.7786 |  | 0.462 |  |
|  |  |  |  | - |  | 9 |  | AX |  | -0.54514 |  | 0.6561 |  | 7.00 |  | -0.8309 |  | 0.433 |  |
|  |  |  |  | - |  | 9 |  | B |  | 2.50000 |  | 0.7336 |  | 7.00 |  | 3.4078 |  | 0.011 |  |
|  |  |  |  | - |  | 9 |  | BY |  | 2.19444 |  | 0.7137 |  | 7.00 |  | 3.0748 |  | 0.018 |  |
|  |  |  |  | - |  | 10 |  | A |  | -0.76042 |  | 0.7658 |  | 7.00 |  | -0.9930 |  | 0.354 |  |
|  |  |  |  | - |  | 10 |  | AX |  | -0.33333 |  | 0.5923 |  | 7.00 |  | -0.5628 |  | 0.591 |  |
|  |  |  |  | - |  | 10 |  | B |  | 2.39583 |  | 0.6481 |  | 7.00 |  | 3.6965 |  | 0.008 |  |
|  |  |  |  | - |  | 10 |  | BY |  | 2.18056 |  | 0.6341 |  | 7.00 |  | 3.4388 |  | 0.011 |  |
|  |  | AX |  | - |  | 2 |  | B |  | 2.40278 |  | 0.5490 |  | 7.00 |  | 4.3767 |  | 0.003 |  |
|  |  |  |  | - |  | 2 |  | BY |  | 1.48611 |  | 0.4171 |  | 7.00 |  | 3.5633 |  | 0.009 |  |
|  |  |  |  | - |  | 3 |  | A |  | 0.15278 |  | 0.3474 |  | 7.00 |  | 0.4398 |  | 0.673 |  |
|  |  |  |  | - |  | 3 |  | AX |  | -0.25347 |  | 0.2211 |  | 7.00 |  | -1.1463 |  | 0.289 |  |
|  |  |  |  | - |  | 3 |  | B |  | 2.39236 |  | 0.6489 |  | 7.00 |  | 3.6867 |  | 0.008 |  |
|  |  |  |  | - |  | 3 |  | BY |  | 1.15625 |  | 0.4514 |  | 7.00 |  | 2.5615 |  | 0.037 |  |
|  |  |  |  | - |  | 4 |  | A |  | 0.18403 |  | 0.3974 |  | 7.00 |  | 0.4631 |  | 0.657 |  |
|  |  |  |  | - |  | 4 |  | AX |  | 0.16667 |  | 0.3597 |  | 7.00 |  | 0.4634 |  | 0.657 |  |
|  |  |  |  | - |  | 4 |  | B |  | 2.81944 |  | 0.5946 |  | 7.00 |  | 4.7417 |  | 0.002 |  |
|  |  |  |  | - |  | 4 |  | BY |  | 1.28819 |  | 0.5749 |  | 7.00 |  | 2.2407 |  | 0.060 |  |
|  |  |  |  | - |  | 5 |  | A |  | 0.55903 |  | 0.4142 |  | 7.00 |  | 1.3496 |  | 0.219 |  |
|  |  |  |  | - |  | 5 |  | AX |  | 0.11458 |  | 0.3882 |  | 7.00 |  | 0.2952 |  | 0.776 |  |
|  |  |  |  | - |  | 5 |  | B |  | 2.50694 |  | 0.4824 |  | 7.00 |  | 5.1968 |  | 0.001 |  |
|  |  |  |  | - |  | 5 |  | BY |  | 1.73958 |  | 0.4690 |  | 7.00 |  | 3.7091 |  | 0.008 |  |
|  |  |  |  | - |  | 6 |  | A |  | 0.09028 |  | 0.4387 |  | 7.00 |  | 0.2058 |  | 0.843 |  |
|  |  |  |  | - |  | 6 |  | AX |  | -0.11472 |  | 0.4835 |  | 7.00 |  | -0.2373 |  | 0.819 |  |
|  |  |  |  | - |  | 6 |  | B |  | 2.75694 |  | 0.5938 |  | 7.00 |  | 4.6429 |  | 0.002 |  |
|  |  |  |  | - |  | 6 |  | BY |  | 1.94097 |  | 0.4314 |  | 7.00 |  | 4.4989 |  | 0.003 |  |
|  |  |  |  | - |  | 7 |  | A |  | 0.26736 |  | 0.4309 |  | 7.00 |  | 0.6205 |  | 0.555 |  |
|  |  |  |  | - |  | 7 |  | AX |  | 0.17708 |  | 0.4499 |  | 7.00 |  | 0.3936 |  | 0.706 |  |
|  |  |  |  | - |  | 7 |  | B |  | 2.93403 |  | 0.6460 |  | 7.00 |  | 4.5418 |  | 0.003 |  |
|  |  |  |  | - |  | 7 |  | BY |  | 2.21528 |  | 0.5466 |  | 7.00 |  | 4.0528 |  | 0.005 |  |
|  |  |  |  | - |  | 8 |  | A |  | -0.33681 |  | 0.7823 |  | 7.00 |  | -0.4305 |  | 0.680 |  |
|  |  |  |  | - |  | 8 |  | AX |  | -0.13542 |  | 0.6870 |  | 7.00 |  | -0.1971 |  | 0.849 |  |
|  |  |  |  | - |  | 8 |  | B |  | 3.01736 |  | 0.6239 |  | 7.00 |  | 4.8365 |  | 0.002 |  |
|  |  |  |  | - |  | 8 |  | BY |  | 2.39583 |  | 0.5744 |  | 7.00 |  | 4.1712 |  | 0.004 |  |
|  |  |  |  | - |  | 9 |  | A |  | -0.20139 |  | 0.9337 |  | 7.00 |  | -0.2157 |  | 0.835 |  |
|  |  |  |  | - |  | 9 |  | AX |  | -0.02778 |  | 0.6979 |  | 7.00 |  | -0.0398 |  | 0.969 |  |
|  |  |  |  | - |  | 9 |  | B |  | 3.01736 |  | 0.6810 |  | 7.00 |  | 4.4310 |  | 0.003 |  |
|  |  |  |  | - |  | 9 |  | BY |  | 2.71181 |  | 0.6717 |  | 7.00 |  | 4.0374 |  | 0.005 |  |
|  |  |  |  | - |  | 10 |  | A |  | -0.24306 |  | 0.7870 |  | 7.00 |  | -0.3088 |  | 0.766 |  |
|  |  |  |  | - |  | 10 |  | AX |  | 0.18403 |  | 0.5906 |  | 7.00 |  | 0.3116 |  | 0.764 |  |
|  |  |  |  | - |  | 10 |  | B |  | 2.91319 |  | 0.5873 |  | 7.00 |  | 4.9601 |  | 0.002 |  |
|  |  |  |  | - |  | 10 |  | BY |  | 2.69792 |  | 0.5939 |  | 7.00 |  | 4.5429 |  | 0.003 |  |
|  |  | B |  | - |  | 2 |  | BY |  | -0.91667 |  | 0.1818 |  | 7.00 |  | -5.0408 |  | 0.001 |  |
|  |  |  |  | - |  | 3 |  | A |  | -2.25000 |  | 0.4061 |  | 7.00 |  | -5.5403 |  | < .001 |  |
|  |  |  |  | - |  | 3 |  | AX |  | -2.65625 |  | 0.5056 |  | 7.00 |  | -5.2535 |  | 0.001 |  |
|  |  |  |  | - |  | 3 |  | B |  | -0.01042 |  | 0.3264 |  | 7.00 |  | -0.0319 |  | 0.975 |  |
|  |  |  |  | - |  | 3 |  | BY |  | -1.24653 |  | 0.2408 |  | 7.00 |  | -5.1763 |  | 0.001 |  |
|  |  |  |  | - |  | 4 |  | A |  | -2.21875 |  | 0.4992 |  | 7.00 |  | -4.4443 |  | 0.003 |  |
|  |  |  |  | - |  | 4 |  | AX |  | -2.23611 |  | 0.4749 |  | 7.00 |  | -4.7086 |  | 0.002 |  |
|  |  |  |  | - |  | 4 |  | B |  | 0.41667 |  | 0.2480 |  | 7.00 |  | 1.6801 |  | 0.137 |  |
|  |  |  |  | - |  | 4 |  | BY |  | -1.11458 |  | 0.1235 |  | 7.00 |  | -9.0253 |  | < .001 |  |
|  |  |  |  | - |  | 5 |  | A |  | -1.84375 |  | 0.3758 |  | 7.00 |  | -4.9061 |  | 0.002 |  |
|  |  |  |  | - |  | 5 |  | AX |  | -2.28819 |  | 0.4872 |  | 7.00 |  | -4.6970 |  | 0.002 |  |
|  |  |  |  | - |  | 5 |  | B |  | 0.10417 |  | 0.2366 |  | 7.00 |  | 0.4402 |  | 0.673 |  |
|  |  |  |  | - |  | 5 |  | BY |  | -0.66319 |  | 0.1986 |  | 7.00 |  | -3.3392 |  | 0.012 |  |
|  |  |  |  | - |  | 6 |  | A |  | -2.31250 |  | 0.6200 |  | 7.00 |  | -3.7300 |  | 0.007 |  |
|  |  |  |  | - |  | 6 |  | AX |  | -2.51750 |  | 0.6559 |  | 7.00 |  | -3.8380 |  | 0.006 |  |
|  |  |  |  | - |  | 6 |  | B |  | 0.35417 |  | 0.1914 |  | 7.00 |  | 1.8501 |  | 0.107 |  |
|  |  |  |  | - |  | 6 |  | BY |  | -0.46181 |  | 0.1548 |  | 7.00 |  | -2.9826 |  | 0.020 |  |
|  |  |  |  | - |  | 7 |  | A |  | -2.13542 |  | 0.5897 |  | 7.00 |  | -3.6214 |  | 0.008 |  |
|  |  |  |  | - |  | 7 |  | AX |  | -2.22569 |  | 0.6510 |  | 7.00 |  | -3.4190 |  | 0.011 |  |
|  |  |  |  | - |  | 7 |  | B |  | 0.53125 |  | 0.2929 |  | 7.00 |  | 1.8137 |  | 0.113 |  |
|  |  |  |  | - |  | 7 |  | BY |  | -0.18750 |  | 0.1637 |  | 7.00 |  | -1.1452 |  | 0.290 |  |
|  |  |  |  | - |  | 8 |  | A |  | -2.73958 |  | 1.0075 |  | 7.00 |  | -2.7193 |  | 0.030 |  |
|  |  |  |  | - |  | 8 |  | AX |  | -2.53819 |  | 0.8827 |  | 7.00 |  | -2.8755 |  | 0.024 |  |
|  |  |  |  | - |  | 8 |  | B |  | 0.61458 |  | 0.2559 |  | 7.00 |  | 2.4021 |  | 0.047 |  |
|  |  |  |  | - |  | 8 |  | BY |  | -0.00694 |  | 0.1240 |  | 7.00 |  | -0.0560 |  | 0.957 |  |
|  |  |  |  | - |  | 9 |  | A |  | -2.60417 |  | 1.1377 |  | 7.00 |  | -2.2890 |  | 0.056 |  |
|  |  |  |  | - |  | 9 |  | AX |  | -2.43056 |  | 0.8878 |  | 7.00 |  | -2.7379 |  | 0.029 |  |
|  |  |  |  | - |  | 9 |  | B |  | 0.61458 |  | 0.3318 |  | 7.00 |  | 1.8522 |  | 0.106 |  |
|  |  |  |  | - |  | 9 |  | BY |  | 0.30903 |  | 0.2945 |  | 7.00 |  | 1.0495 |  | 0.329 |  |
|  |  |  |  | - |  | 10 |  | A |  | -2.64583 |  | 0.9037 |  | 7.00 |  | -2.9278 |  | 0.022 |  |
|  |  |  |  | - |  | 10 |  | AX |  | -2.21875 |  | 0.6863 |  | 7.00 |  | -3.2330 |  | 0.014 |  |
|  |  |  |  | - |  | 10 |  | B |  | 0.51042 |  | 0.2349 |  | 7.00 |  | 2.1731 |  | 0.066 |  |
|  |  |  |  | - |  | 10 |  | BY |  | 0.29514 |  | 0.2446 |  | 7.00 |  | 1.2064 |  | 0.267 |  |
|  |  | BY |  | - |  | 3 |  | A |  | -1.33333 |  | 0.3200 |  | 7.00 |  | -4.1660 |  | 0.004 |  |
|  |  |  |  | - |  | 3 |  | AX |  | -1.73958 |  | 0.3694 |  | 7.00 |  | -4.7096 |  | 0.002 |  |
|  |  |  |  | - |  | 3 |  | B |  | 0.90625 |  | 0.4376 |  | 7.00 |  | 2.0710 |  | 0.077 |  |
|  |  |  |  | - |  | 3 |  | BY |  | -0.32986 |  | 0.1185 |  | 7.00 |  | -2.7840 |  | 0.027 |  |
|  |  |  |  | - |  | 4 |  | A |  | -1.30208 |  | 0.3818 |  | 7.00 |  | -3.4108 |  | 0.011 |  |
|  |  |  |  | - |  | 4 |  | AX |  | -1.31944 |  | 0.3694 |  | 7.00 |  | -3.5714 |  | 0.009 |  |
|  |  |  |  | - |  | 4 |  | B |  | 1.33333 |  | 0.3394 |  | 7.00 |  | 3.9290 |  | 0.006 |  |
|  |  |  |  | - |  | 4 |  | BY |  | -0.19792 |  | 0.1838 |  | 7.00 |  | -1.0766 |  | 0.317 |  |
|  |  |  |  | - |  | 5 |  | A |  | -0.92708 |  | 0.2991 |  | 7.00 |  | -3.0993 |  | 0.017 |  |
|  |  |  |  | - |  | 5 |  | AX |  | -1.37153 |  | 0.3788 |  | 7.00 |  | -3.6211 |  | 0.008 |  |
|  |  |  |  | - |  | 5 |  | B |  | 1.02083 |  | 0.3023 |  | 7.00 |  | 3.3771 |  | 0.012 |  |
|  |  |  |  | - |  | 5 |  | BY |  | 0.25347 |  | 0.1925 |  | 7.00 |  | 1.3169 |  | 0.229 |  |
|  |  |  |  | - |  | 6 |  | A |  | -1.39583 |  | 0.5240 |  | 7.00 |  | -2.6638 |  | 0.032 |  |
|  |  |  |  | - |  | 6 |  | AX |  | -1.60083 |  | 0.5459 |  | 7.00 |  | -2.9322 |  | 0.022 |  |
|  |  |  |  | - |  | 6 |  | B |  | 1.27083 |  | 0.3128 |  | 7.00 |  | 4.0632 |  | 0.005 |  |
|  |  |  |  | - |  | 6 |  | BY |  | 0.45486 |  | 0.1400 |  | 7.00 |  | 3.2497 |  | 0.014 |  |
|  |  |  |  | - |  | 7 |  | A |  | -1.21875 |  | 0.5026 |  | 7.00 |  | -2.4248 |  | 0.046 |  |
|  |  |  |  | - |  | 7 |  | AX |  | -1.30903 |  | 0.5431 |  | 7.00 |  | -2.4104 |  | 0.047 |  |
|  |  |  |  | - |  | 7 |  | B |  | 1.44792 |  | 0.4060 |  | 7.00 |  | 3.5663 |  | 0.009 |  |
|  |  |  |  | - |  | 7 |  | BY |  | 0.72917 |  | 0.2324 |  | 7.00 |  | 3.1377 |  | 0.016 |  |
|  |  |  |  | - |  | 8 |  | A |  | -1.82292 |  | 0.9206 |  | 7.00 |  | -1.9801 |  | 0.088 |  |
|  |  |  |  | - |  | 8 |  | AX |  | -1.62153 |  | 0.7893 |  | 7.00 |  | -2.0544 |  | 0.079 |  |
|  |  |  |  | - |  | 8 |  | B |  | 1.53125 |  | 0.3506 |  | 7.00 |  | 4.3676 |  | 0.003 |  |
|  |  |  |  | - |  | 8 |  | BY |  | 0.90972 |  | 0.2240 |  | 7.00 |  | 4.0613 |  | 0.005 |  |
|  |  |  |  | - |  | 9 |  | A |  | -1.68750 |  | 1.0687 |  | 7.00 |  | -1.5790 |  | 0.158 |  |
|  |  |  |  | - |  | 9 |  | AX |  | -1.51389 |  | 0.8024 |  | 7.00 |  | -1.8866 |  | 0.101 |  |
|  |  |  |  | - |  | 9 |  | B |  | 1.53125 |  | 0.4241 |  | 7.00 |  | 3.6104 |  | 0.009 |  |
|  |  |  |  | - |  | 9 |  | BY |  | 1.22569 |  | 0.3976 |  | 7.00 |  | 3.0828 |  | 0.018 |  |
|  |  |  |  | - |  | 10 |  | A |  | -1.72917 |  | 0.8327 |  | 7.00 |  | -2.0767 |  | 0.076 |  |
|  |  |  |  | - |  | 10 |  | AX |  | -1.30208 |  | 0.6153 |  | 7.00 |  | -2.1163 |  | 0.072 |  |
|  |  |  |  | - |  | 10 |  | B |  | 1.42708 |  | 0.3081 |  | 7.00 |  | 4.6317 |  | 0.002 |  |
|  |  |  |  | - |  | 10 |  | BY |  | 1.21181 |  | 0.3104 |  | 7.00 |  | 3.9036 |  | 0.006 |  |
| 3 |  | A |  | - |  | 3 |  | AX |  | -0.40625 |  | 0.2623 |  | 7.00 |  | -1.5487 |  | 0.165 |  |
|  |  |  |  | - |  | 3 |  | B |  | 2.23958 |  | 0.4579 |  | 7.00 |  | 4.8909 |  | 0.002 |  |
|  |  |  |  | - |  | 3 |  | BY |  | 1.00347 |  | 0.3450 |  | 7.00 |  | 2.9089 |  | 0.023 |  |
|  |  |  |  | - |  | 4 |  | A |  | 0.03125 |  | 0.2388 |  | 7.00 |  | 0.1309 |  | 0.900 |  |
|  |  |  |  | - |  | 4 |  | AX |  | 0.01389 |  | 0.2315 |  | 7.00 |  | 0.0600 |  | 0.954 |  |
|  |  |  |  | - |  | 4 |  | B |  | 2.66667 |  | 0.4432 |  | 7.00 |  | 6.0168 |  | < .001 |  |
|  |  |  |  | - |  | 4 |  | BY |  | 1.13542 |  | 0.4153 |  | 7.00 |  | 2.7338 |  | 0.029 |  |
|  |  |  |  | - |  | 5 |  | A |  | 0.40625 |  | 0.3390 |  | 7.00 |  | 1.1983 |  | 0.270 |  |
|  |  |  |  | - |  | 5 |  | AX |  | -0.03819 |  | 0.2386 |  | 7.00 |  | -0.1601 |  | 0.877 |  |
|  |  |  |  | - |  | 5 |  | B |  | 2.35417 |  | 0.4008 |  | 7.00 |  | 5.8735 |  | < .001 |  |
|  |  |  |  | - |  | 5 |  | BY |  | 1.58681 |  | 0.2925 |  | 7.00 |  | 5.4243 |  | < .001 |  |
|  |  |  |  | - |  | 6 |  | A |  | -0.06250 |  | 0.3306 |  | 7.00 |  | -0.1890 |  | 0.855 |  |
|  |  |  |  | - |  | 6 |  | AX |  | -0.26750 |  | 0.3378 |  | 7.00 |  | -0.7920 |  | 0.454 |  |
|  |  |  |  | - |  | 6 |  | B |  | 2.60417 |  | 0.4190 |  | 7.00 |  | 6.2157 |  | < .001 |  |
|  |  |  |  | - |  | 6 |  | BY |  | 1.78819 |  | 0.2966 |  | 7.00 |  | 6.0293 |  | < .001 |  |
|  |  |  |  | - |  | 7 |  | A |  | 0.11458 |  | 0.2813 |  | 7.00 |  | 0.4074 |  | 0.696 |  |
|  |  |  |  | - |  | 7 |  | AX |  | 0.02431 |  | 0.3135 |  | 7.00 |  | 0.0775 |  | 0.940 |  |
|  |  |  |  | - |  | 7 |  | B |  | 2.78125 |  | 0.4925 |  | 7.00 |  | 5.6474 |  | < .001 |  |
|  |  |  |  | - |  | 7 |  | BY |  | 2.06250 |  | 0.4364 |  | 7.00 |  | 4.7266 |  | 0.002 |  |
|  |  |  |  | - |  | 8 |  | A |  | -0.48958 |  | 0.6787 |  | 7.00 |  | -0.7213 |  | 0.494 |  |
|  |  |  |  | - |  | 8 |  | AX |  | -0.28819 |  | 0.5470 |  | 7.00 |  | -0.5269 |  | 0.615 |  |
|  |  |  |  | - |  | 8 |  | B |  | 2.86458 |  | 0.4826 |  | 7.00 |  | 5.9361 |  | < .001 |  |
|  |  |  |  | - |  | 8 |  | BY |  | 2.24306 |  | 0.4150 |  | 7.00 |  | 5.4054 |  | 0.001 |  |
|  |  |  |  | - |  | 9 |  | A |  | -0.35417 |  | 0.8267 |  | 7.00 |  | -0.4284 |  | 0.681 |  |
|  |  |  |  | - |  | 9 |  | AX |  | -0.18056 |  | 0.5593 |  | 7.00 |  | -0.3228 |  | 0.756 |  |
|  |  |  |  | - |  | 9 |  | B |  | 2.86458 |  | 0.5242 |  | 7.00 |  | 5.4647 |  | < .001 |  |
|  |  |  |  | - |  | 9 |  | BY |  | 2.55903 |  | 0.5621 |  | 7.00 |  | 4.5526 |  | 0.003 |  |
|  |  |  |  | - |  | 10 |  | A |  | -0.39583 |  | 0.5924 |  | 7.00 |  | -0.6682 |  | 0.525 |  |
|  |  |  |  | - |  | 10 |  | AX |  | 0.03125 |  | 0.3625 |  | 7.00 |  | 0.0862 |  | 0.934 |  |
|  |  |  |  | - |  | 10 |  | B |  | 2.76042 |  | 0.4552 |  | 7.00 |  | 6.0643 |  | < .001 |  |
|  |  |  |  | - |  | 10 |  | BY |  | 2.54514 |  | 0.4594 |  | 7.00 |  | 5.5400 |  | < .001 |  |
|  |  | AX |  | - |  | 3 |  | B |  | 2.64583 |  | 0.6413 |  | 7.00 |  | 4.1259 |  | 0.004 |  |
|  |  |  |  | - |  | 3 |  | BY |  | 1.40972 |  | 0.4012 |  | 7.00 |  | 3.5138 |  | 0.010 |  |
|  |  |  |  | - |  | 4 |  | A |  | 0.43750 |  | 0.2874 |  | 7.00 |  | 1.5222 |  | 0.172 |  |
|  |  |  |  | - |  | 4 |  | AX |  | 0.42014 |  | 0.1987 |  | 7.00 |  | 2.1146 |  | 0.072 |  |
|  |  |  |  | - |  | 4 |  | B |  | 3.07292 |  | 0.5757 |  | 7.00 |  | 5.3382 |  | 0.001 |  |
|  |  |  |  | - |  | 4 |  | BY |  | 1.54167 |  | 0.5135 |  | 7.00 |  | 3.0024 |  | 0.020 |  |
|  |  |  |  | - |  | 5 |  | A |  | 0.81250 |  | 0.2988 |  | 7.00 |  | 2.7188 |  | 0.030 |  |
|  |  |  |  | - |  | 5 |  | AX |  | 0.36806 |  | 0.2502 |  | 7.00 |  | 1.4713 |  | 0.185 |  |
|  |  |  |  | - |  | 5 |  | B |  | 2.76042 |  | 0.5149 |  | 7.00 |  | 5.3607 |  | 0.001 |  |
|  |  |  |  | - |  | 5 |  | BY |  | 1.99306 |  | 0.4203 |  | 7.00 |  | 4.7415 |  | 0.002 |  |
|  |  |  |  | - |  | 6 |  | A |  | 0.34375 |  | 0.3704 |  | 7.00 |  | 0.9281 |  | 0.384 |  |
|  |  |  |  | - |  | 6 |  | AX |  | 0.13875 |  | 0.3983 |  | 7.00 |  | 0.3483 |  | 0.738 |  |
|  |  |  |  | - |  | 6 |  | B |  | 3.01042 |  | 0.5767 |  | 7.00 |  | 5.2198 |  | 0.001 |  |
|  |  |  |  | - |  | 6 |  | BY |  | 2.19444 |  | 0.4076 |  | 7.00 |  | 5.3837 |  | 0.001 |  |
|  |  |  |  | - |  | 7 |  | A |  | 0.52083 |  | 0.3831 |  | 7.00 |  | 1.3595 |  | 0.216 |  |
|  |  |  |  | - |  | 7 |  | AX |  | 0.43056 |  | 0.3556 |  | 7.00 |  | 1.2107 |  | 0.265 |  |
|  |  |  |  | - |  | 7 |  | B |  | 3.18750 |  | 0.6465 |  | 7.00 |  | 4.9301 |  | 0.002 |  |
|  |  |  |  | - |  | 7 |  | BY |  | 2.46875 |  | 0.5443 |  | 7.00 |  | 4.5357 |  | 0.003 |  |
|  |  |  |  | - |  | 8 |  | A |  | -0.08333 |  | 0.7322 |  | 7.00 |  | -0.1138 |  | 0.913 |  |
|  |  |  |  | - |  | 8 |  | AX |  | 0.11806 |  | 0.6130 |  | 7.00 |  | 0.1926 |  | 0.853 |  |
|  |  |  |  | - |  | 8 |  | B |  | 3.27083 |  | 0.6117 |  | 7.00 |  | 5.3470 |  | 0.001 |  |
|  |  |  |  | - |  | 8 |  | BY |  | 2.64931 |  | 0.5390 |  | 7.00 |  | 4.9153 |  | 0.002 |  |
|  |  |  |  | - |  | 9 |  | A |  | 0.05208 |  | 0.9088 |  | 7.00 |  | 0.0573 |  | 0.956 |  |
|  |  |  |  | - |  | 9 |  | AX |  | 0.22569 |  | 0.6447 |  | 7.00 |  | 0.3501 |  | 0.737 |  |
|  |  |  |  | - |  | 9 |  | B |  | 3.27083 |  | 0.6740 |  | 7.00 |  | 4.8527 |  | 0.002 |  |
|  |  |  |  | - |  | 9 |  | BY |  | 2.96528 |  | 0.6849 |  | 7.00 |  | 4.3296 |  | 0.003 |  |
|  |  |  |  | - |  | 10 |  | A |  | 0.01042 |  | 0.7101 |  | 7.00 |  | 0.0147 |  | 0.989 |  |
|  |  |  |  | - |  | 10 |  | AX |  | 0.43750 |  | 0.4971 |  | 7.00 |  | 0.8801 |  | 0.408 |  |
|  |  |  |  | - |  | 10 |  | B |  | 3.16667 |  | 0.5712 |  | 7.00 |  | 5.5436 |  | < .001 |  |
|  |  |  |  | - |  | 10 |  | BY |  | 2.95139 |  | 0.5824 |  | 7.00 |  | 5.0678 |  | 0.001 |  |
|  |  | B |  | - |  | 3 |  | BY |  | -1.23611 |  | 0.4855 |  | 7.00 |  | -2.5458 |  | 0.038 |  |
|  |  |  |  | - |  | 4 |  | A |  | -2.20833 |  | 0.6170 |  | 7.00 |  | -3.5791 |  | 0.009 |  |
|  |  |  |  | - |  | 4 |  | AX |  | -2.22569 |  | 0.5959 |  | 7.00 |  | -3.7352 |  | 0.007 |  |
|  |  |  |  | - |  | 4 |  | B |  | 0.42708 |  | 0.1840 |  | 7.00 |  | 2.3217 |  | 0.053 |  |
|  |  |  |  | - |  | 4 |  | BY |  | -1.10417 |  | 0.3740 |  | 7.00 |  | -2.9523 |  | 0.021 |  |
|  |  |  |  | - |  | 5 |  | A |  | -1.83333 |  | 0.5643 |  | 7.00 |  | -3.2488 |  | 0.014 |  |
|  |  |  |  | - |  | 5 |  | AX |  | -2.27778 |  | 0.5871 |  | 7.00 |  | -3.8797 |  | 0.006 |  |
|  |  |  |  | - |  | 5 |  | B |  | 0.11458 |  | 0.2414 |  | 7.00 |  | 0.4747 |  | 0.649 |  |
|  |  |  |  | - |  | 5 |  | BY |  | -0.65278 |  | 0.3149 |  | 7.00 |  | -2.0731 |  | 0.077 |  |
|  |  |  |  | - |  | 6 |  | A |  | -2.30208 |  | 0.6737 |  | 7.00 |  | -3.4171 |  | 0.011 |  |
|  |  |  |  | - |  | 6 |  | AX |  | -2.50708 |  | 0.7076 |  | 7.00 |  | -3.5431 |  | 0.009 |  |
|  |  |  |  | - |  | 6 |  | B |  | 0.36458 |  | 0.1485 |  | 7.00 |  | 2.4548 |  | 0.044 |  |
|  |  |  |  | - |  | 6 |  | BY |  | -0.45139 |  | 0.3561 |  | 7.00 |  | -1.2676 |  | 0.245 |  |
|  |  |  |  | - |  | 7 |  | A |  | -2.12500 |  | 0.5916 |  | 7.00 |  | -3.5922 |  | 0.009 |  |
|  |  |  |  | - |  | 7 |  | AX |  | -2.21528 |  | 0.6757 |  | 7.00 |  | -3.2783 |  | 0.014 |  |
|  |  |  |  | - |  | 7 |  | B |  | 0.54167 |  | 0.1551 |  | 7.00 |  | 3.4923 |  | 0.010 |  |
|  |  |  |  | - |  | 7 |  | BY |  | -0.17708 |  | 0.2980 |  | 7.00 |  | -0.5942 |  | 0.571 |  |
|  |  |  |  | - |  | 8 |  | A |  | -2.72917 |  | 0.9501 |  | 7.00 |  | -2.8725 |  | 0.024 |  |
|  |  |  |  | - |  | 8 |  | AX |  | -2.52778 |  | 0.8611 |  | 7.00 |  | -2.9356 |  | 0.022 |  |
|  |  |  |  | - |  | 8 |  | B |  | 0.62500 |  | 0.2635 |  | 7.00 |  | 2.3717 |  | 0.049 |  |
|  |  |  |  | - |  | 8 |  | BY |  | 0.00347 |  | 0.2771 |  | 7.00 |  | 0.0125 |  | 0.990 |  |
|  |  |  |  | - |  | 9 |  | A |  | -2.59375 |  | 1.0083 |  | 7.00 |  | -2.5723 |  | 0.037 |  |
|  |  |  |  | - |  | 9 |  | AX |  | -2.42014 |  | 0.8226 |  | 7.00 |  | -2.9421 |  | 0.022 |  |
|  |  |  |  | - |  | 9 |  | B |  | 0.62500 |  | 0.2764 |  | 7.00 |  | 2.2613 |  | 0.058 |  |
|  |  |  |  | - |  | 9 |  | BY |  | 0.31944 |  | 0.2914 |  | 7.00 |  | 1.0963 |  | 0.309 |  |
|  |  |  |  | - |  | 10 |  | A |  | -2.63542 |  | 0.8188 |  | 7.00 |  | -3.2188 |  | 0.015 |  |
|  |  |  |  | - |  | 10 |  | AX |  | -2.20833 |  | 0.6283 |  | 7.00 |  | -3.5147 |  | 0.010 |  |
|  |  |  |  | - |  | 10 |  | B |  | 0.52083 |  | 0.3192 |  | 7.00 |  | 1.6318 |  | 0.147 |  |
|  |  |  |  | - |  | 10 |  | BY |  | 0.30556 |  | 0.2806 |  | 7.00 |  | 1.0888 |  | 0.312 |  |
|  |  | BY |  | - |  | 4 |  | A |  | -0.97222 |  | 0.3422 |  | 7.00 |  | -2.8409 |  | 0.025 |  |
|  |  |  |  | - |  | 4 |  | AX |  | -0.98958 |  | 0.3663 |  | 7.00 |  | -2.7016 |  | 0.031 |  |
|  |  |  |  | - |  | 4 |  | B |  | 1.66319 |  | 0.3965 |  | 7.00 |  | 4.1949 |  | 0.004 |  |
|  |  |  |  | - |  | 4 |  | BY |  | 0.13194 |  | 0.1909 |  | 7.00 |  | 0.6911 |  | 0.512 |  |
|  |  |  |  | - |  | 5 |  | A |  | -0.59722 |  | 0.2817 |  | 7.00 |  | -2.1203 |  | 0.072 |  |
|  |  |  |  | - |  | 5 |  | AX |  | -1.04167 |  | 0.3605 |  | 7.00 |  | -2.8898 |  | 0.023 |  |
|  |  |  |  | - |  | 5 |  | B |  | 1.35069 |  | 0.3513 |  | 7.00 |  | 3.8446 |  | 0.006 |  |
|  |  |  |  | - |  | 5 |  | BY |  | 0.58333 |  | 0.2152 |  | 7.00 |  | 2.7103 |  | 0.030 |  |
|  |  |  |  | - |  | 6 |  | A |  | -1.06597 |  | 0.4898 |  | 7.00 |  | -2.1763 |  | 0.066 |  |
|  |  |  |  | - |  | 6 |  | AX |  | -1.27097 |  | 0.5113 |  | 7.00 |  | -2.4860 |  | 0.042 |  |
|  |  |  |  | - |  | 6 |  | B |  | 1.60069 |  | 0.3553 |  | 7.00 |  | 4.5052 |  | 0.003 |  |
|  |  |  |  | - |  | 6 |  | BY |  | 0.78472 |  | 0.2023 |  | 7.00 |  | 3.8781 |  | 0.006 |  |
|  |  |  |  | - |  | 7 |  | A |  | -0.88889 |  | 0.4861 |  | 7.00 |  | -1.8288 |  | 0.110 |  |
|  |  |  |  | - |  | 7 |  | AX |  | -0.97917 |  | 0.5446 |  | 7.00 |  | -1.7979 |  | 0.115 |  |
|  |  |  |  | - |  | 7 |  | B |  | 1.77778 |  | 0.4450 |  | 7.00 |  | 3.9947 |  | 0.005 |  |
|  |  |  |  | - |  | 7 |  | BY |  | 1.05903 |  | 0.2705 |  | 7.00 |  | 3.9156 |  | 0.006 |  |
|  |  |  |  | - |  | 8 |  | A |  | -1.49306 |  | 0.9191 |  | 7.00 |  | -1.6244 |  | 0.148 |  |
|  |  |  |  | - |  | 8 |  | AX |  | -1.29167 |  | 0.7760 |  | 7.00 |  | -1.6644 |  | 0.140 |  |
|  |  |  |  | - |  | 8 |  | B |  | 1.86111 |  | 0.3574 |  | 7.00 |  | 5.2081 |  | 0.001 |  |
|  |  |  |  | - |  | 8 |  | BY |  | 1.23958 |  | 0.2834 |  | 7.00 |  | 4.3744 |  | 0.003 |  |
|  |  |  |  | - |  | 9 |  | A |  | -1.35764 |  | 1.0802 |  | 7.00 |  | -1.2568 |  | 0.249 |  |
|  |  |  |  | - |  | 9 |  | AX |  | -1.18403 |  | 0.8051 |  | 7.00 |  | -1.4707 |  | 0.185 |  |
|  |  |  |  | - |  | 9 |  | B |  | 1.86111 |  | 0.4711 |  | 7.00 |  | 3.9505 |  | 0.006 |  |
|  |  |  |  | - |  | 9 |  | BY |  | 1.55556 |  | 0.4331 |  | 7.00 |  | 3.5919 |  | 0.009 |  |
|  |  |  |  | - |  | 10 |  | A |  | -1.39931 |  | 0.8360 |  | 7.00 |  | -1.6738 |  | 0.138 |  |
|  |  |  |  | - |  | 10 |  | AX |  | -0.97222 |  | 0.6220 |  | 7.00 |  | -1.5631 |  | 0.162 |  |
|  |  |  |  | - |  | 10 |  | B |  | 1.75694 |  | 0.3445 |  | 7.00 |  | 5.0993 |  | 0.001 |  |
|  |  |  |  | - |  | 10 |  | BY |  | 1.54167 |  | 0.3308 |  | 7.00 |  | 4.6604 |  | 0.002 |  |
| 4 |  | A |  | - |  | 4 |  | AX |  | -0.01736 |  | 0.1568 |  | 7.00 |  | -0.1107 |  | 0.915 |  |
|  |  |  |  | - |  | 4 |  | B |  | 2.63542 |  | 0.5724 |  | 7.00 |  | 4.6044 |  | 0.002 |  |
|  |  |  |  | - |  | 4 |  | BY |  | 1.10417 |  | 0.4628 |  | 7.00 |  | 2.3858 |  | 0.048 |  |
|  |  |  |  | - |  | 5 |  | A |  | 0.37500 |  | 0.2795 |  | 7.00 |  | 1.3416 |  | 0.222 |  |
|  |  |  |  | - |  | 5 |  | AX |  | -0.06944 |  | 0.1337 |  | 7.00 |  | -0.5193 |  | 0.620 |  |
|  |  |  |  | - |  | 5 |  | B |  | 2.32292 |  | 0.5314 |  | 7.00 |  | 4.3716 |  | 0.003 |  |
|  |  |  |  | - |  | 5 |  | BY |  | 1.55556 |  | 0.3628 |  | 7.00 |  | 4.2874 |  | 0.004 |  |
|  |  |  |  | - |  | 6 |  | A |  | -0.09375 |  | 0.1812 |  | 7.00 |  | -0.5173 |  | 0.621 |  |
|  |  |  |  | - |  | 6 |  | AX |  | -0.29875 |  | 0.1942 |  | 7.00 |  | -1.5386 |  | 0.168 |  |
|  |  |  |  | - |  | 6 |  | B |  | 2.57292 |  | 0.5495 |  | 7.00 |  | 4.6823 |  | 0.002 |  |
|  |  |  |  | - |  | 6 |  | BY |  | 1.75694 |  | 0.3972 |  | 7.00 |  | 4.4238 |  | 0.003 |  |
|  |  |  |  | - |  | 7 |  | A |  | 0.08333 |  | 0.2304 |  | 7.00 |  | 0.3617 |  | 0.728 |  |
|  |  |  |  | - |  | 7 |  | AX |  | -0.00694 |  | 0.2829 |  | 7.00 |  | -0.0245 |  | 0.981 |  |
|  |  |  |  | - |  | 7 |  | B |  | 2.75000 |  | 0.6368 |  | 7.00 |  | 4.3185 |  | 0.003 |  |
|  |  |  |  | - |  | 7 |  | BY |  | 2.03125 |  | 0.5417 |  | 7.00 |  | 3.7496 |  | 0.007 |  |
|  |  |  |  | - |  | 8 |  | A |  | -0.52083 |  | 0.6483 |  | 7.00 |  | -0.8034 |  | 0.448 |  |
|  |  |  |  | - |  | 8 |  | AX |  | -0.31944 |  | 0.4869 |  | 7.00 |  | -0.6561 |  | 0.533 |  |
|  |  |  |  | - |  | 8 |  | B |  | 2.83333 |  | 0.5743 |  | 7.00 |  | 4.9332 |  | 0.002 |  |
|  |  |  |  | - |  | 8 |  | BY |  | 2.21181 |  | 0.5354 |  | 7.00 |  | 4.1314 |  | 0.004 |  |
|  |  |  |  | - |  | 9 |  | A |  | -0.38542 |  | 0.8630 |  | 7.00 |  | -0.4466 |  | 0.669 |  |
|  |  |  |  | - |  | 9 |  | AX |  | -0.21181 |  | 0.5650 |  | 7.00 |  | -0.3749 |  | 0.719 |  |
|  |  |  |  | - |  | 9 |  | B |  | 2.83333 |  | 0.6881 |  | 7.00 |  | 4.1177 |  | 0.004 |  |
|  |  |  |  | - |  | 9 |  | BY |  | 2.52778 |  | 0.6903 |  | 7.00 |  | 3.6618 |  | 0.008 |  |
|  |  |  |  | - |  | 10 |  | A |  | -0.42708 |  | 0.6269 |  | 7.00 |  | -0.6813 |  | 0.518 |  |
|  |  |  |  | - |  | 10 |  | AX |  | 1.33e-15 |  | 0.4237 |  | 7.00 |  | 3.14e-15 |  | 1.000 |  |
|  |  |  |  | - |  | 10 |  | B |  | 2.72917 |  | 0.5860 |  | 7.00 |  | 4.6570 |  | 0.002 |  |
|  |  |  |  | - |  | 10 |  | BY |  | 2.51389 |  | 0.5669 |  | 7.00 |  | 4.4343 |  | 0.003 |  |
|  |  | AX |  | - |  | 4 |  | B |  | 2.65278 |  | 0.5403 |  | 7.00 |  | 4.9099 |  | 0.002 |  |
|  |  |  |  | - |  | 4 |  | BY |  | 1.12153 |  | 0.4561 |  | 7.00 |  | 2.4589 |  | 0.044 |  |
|  |  |  |  | - |  | 5 |  | A |  | 0.39236 |  | 0.2067 |  | 7.00 |  | 1.8982 |  | 0.099 |  |
|  |  |  |  | - |  | 5 |  | AX |  | -0.05208 |  | 0.0990 |  | 7.00 |  | -0.5263 |  | 0.615 |  |
|  |  |  |  | - |  | 5 |  | B |  | 2.34028 |  | 0.5067 |  | 7.00 |  | 4.6189 |  | 0.002 |  |
|  |  |  |  | - |  | 5 |  | BY |  | 1.57292 |  | 0.3527 |  | 7.00 |  | 4.4597 |  | 0.003 |  |
|  |  |  |  | - |  | 6 |  | A |  | -0.07639 |  | 0.2368 |  | 7.00 |  | -0.3226 |  | 0.756 |  |
|  |  |  |  | - |  | 6 |  | AX |  | -0.28139 |  | 0.2835 |  | 7.00 |  | -0.9924 |  | 0.354 |  |
|  |  |  |  | - |  | 6 |  | B |  | 2.59028 |  | 0.5335 |  | 7.00 |  | 4.8556 |  | 0.002 |  |
|  |  |  |  | - |  | 6 |  | BY |  | 1.77431 |  | 0.3888 |  | 7.00 |  | 4.5636 |  | 0.003 |  |
|  |  |  |  | - |  | 7 |  | A |  | 0.10069 |  | 0.2766 |  | 7.00 |  | 0.3641 |  | 0.727 |  |
|  |  |  |  | - |  | 7 |  | AX |  | 0.01042 |  | 0.2920 |  | 7.00 |  | 0.0357 |  | 0.973 |  |
|  |  |  |  | - |  | 7 |  | B |  | 2.76736 |  | 0.6178 |  | 7.00 |  | 4.4796 |  | 0.003 |  |
|  |  |  |  | - |  | 7 |  | BY |  | 2.04861 |  | 0.5334 |  | 7.00 |  | 3.8408 |  | 0.006 |  |
|  |  |  |  | - |  | 8 |  | A |  | -0.50347 |  | 0.6698 |  | 7.00 |  | -0.7517 |  | 0.477 |  |
|  |  |  |  | - |  | 8 |  | AX |  | -0.30208 |  | 0.5282 |  | 7.00 |  | -0.5719 |  | 0.585 |  |
|  |  |  |  | - |  | 8 |  | B |  | 2.85069 |  | 0.5644 |  | 7.00 |  | 5.0507 |  | 0.001 |  |
|  |  |  |  | - |  | 8 |  | BY |  | 2.22917 |  | 0.5187 |  | 7.00 |  | 4.2973 |  | 0.004 |  |
|  |  |  |  | - |  | 9 |  | A |  | -0.36806 |  | 0.8752 |  | 7.00 |  | -0.4205 |  | 0.687 |  |
|  |  |  |  | - |  | 9 |  | AX |  | -0.19444 |  | 0.5919 |  | 7.00 |  | -0.3285 |  | 0.752 |  |
|  |  |  |  | - |  | 9 |  | B |  | 2.85069 |  | 0.6745 |  | 7.00 |  | 4.2263 |  | 0.004 |  |
|  |  |  |  | - |  | 9 |  | BY |  | 2.54514 |  | 0.6788 |  | 7.00 |  | 3.7495 |  | 0.007 |  |
|  |  |  |  | - |  | 10 |  | A |  | -0.40972 |  | 0.6532 |  | 7.00 |  | -0.6272 |  | 0.550 |  |
|  |  |  |  | - |  | 10 |  | AX |  | 0.01736 |  | 0.4342 |  | 7.00 |  | 0.0400 |  | 0.969 |  |
|  |  |  |  | - |  | 10 |  | B |  | 2.74653 |  | 0.5696 |  | 7.00 |  | 4.8216 |  | 0.002 |  |
|  |  |  |  | - |  | 10 |  | BY |  | 2.53125 |  | 0.5586 |  | 7.00 |  | 4.5311 |  | 0.003 |  |
|  |  | B |  | - |  | 4 |  | BY |  | -1.53125 |  | 0.2712 |  | 7.00 |  | -5.6468 |  | < .001 |  |
|  |  |  |  | - |  | 5 |  | A |  | -2.26042 |  | 0.4941 |  | 7.00 |  | -4.5746 |  | 0.003 |  |
|  |  |  |  | - |  | 5 |  | AX |  | -2.70486 |  | 0.5249 |  | 7.00 |  | -5.1531 |  | 0.001 |  |
|  |  |  |  | - |  | 5 |  | B |  | -0.31250 |  | 0.2371 |  | 7.00 |  | -1.3178 |  | 0.229 |  |
|  |  |  |  | - |  | 5 |  | BY |  | -1.07986 |  | 0.2233 |  | 7.00 |  | -4.8359 |  | 0.002 |  |
|  |  |  |  | - |  | 6 |  | A |  | -2.72917 |  | 0.6495 |  | 7.00 |  | -4.2021 |  | 0.004 |  |
|  |  |  |  | - |  | 6 |  | AX |  | -2.93417 |  | 0.6746 |  | 7.00 |  | -4.3496 |  | 0.003 |  |
|  |  |  |  | - |  | 6 |  | B |  | -0.06250 |  | 0.1371 |  | 7.00 |  | -0.4560 |  | 0.662 |  |
|  |  |  |  | - |  | 6 |  | BY |  | -0.87847 |  | 0.3065 |  | 7.00 |  | -2.8666 |  | 0.024 |  |
|  |  |  |  | - |  | 7 |  | A |  | -2.55208 |  | 0.5696 |  | 7.00 |  | -4.4809 |  | 0.003 |  |
|  |  |  |  | - |  | 7 |  | AX |  | -2.64236 |  | 0.6305 |  | 7.00 |  | -4.1912 |  | 0.004 |  |
|  |  |  |  | - |  | 7 |  | B |  | 0.11458 |  | 0.2023 |  | 7.00 |  | 0.5665 |  | 0.589 |  |
|  |  |  |  | - |  | 7 |  | BY |  | -0.60417 |  | 0.2188 |  | 7.00 |  | -2.7609 |  | 0.028 |  |
|  |  |  |  | - |  | 8 |  | A |  | -3.15625 |  | 0.9291 |  | 7.00 |  | -3.3972 |  | 0.011 |  |
|  |  |  |  | - |  | 8 |  | AX |  | -2.95486 |  | 0.8386 |  | 7.00 |  | -3.5237 |  | 0.010 |  |
|  |  |  |  | - |  | 8 |  | B |  | 0.19792 |  | 0.2378 |  | 7.00 |  | 0.8324 |  | 0.433 |  |
|  |  |  |  | - |  | 8 |  | BY |  | -0.42361 |  | 0.1850 |  | 7.00 |  | -2.2900 |  | 0.056 |  |
|  |  |  |  | - |  | 9 |  | A |  | -3.02083 |  | 1.0043 |  | 7.00 |  | -3.0079 |  | 0.020 |  |
|  |  |  |  | - |  | 9 |  | AX |  | -2.84722 |  | 0.8027 |  | 7.00 |  | -3.5470 |  | 0.009 |  |
|  |  |  |  | - |  | 9 |  | B |  | 0.19792 |  | 0.3062 |  | 7.00 |  | 0.6464 |  | 0.539 |  |
|  |  |  |  | - |  | 9 |  | BY |  | -0.10764 |  | 0.2733 |  | 7.00 |  | -0.3938 |  | 0.705 |  |
|  |  |  |  | - |  | 10 |  | A |  | -3.06250 |  | 0.8085 |  | 7.00 |  | -3.7878 |  | 0.007 |  |
|  |  |  |  | - |  | 10 |  | AX |  | -2.63542 |  | 0.6164 |  | 7.00 |  | -4.2758 |  | 0.004 |  |
|  |  |  |  | - |  | 10 |  | B |  | 0.09375 |  | 0.3035 |  | 7.00 |  | 0.3089 |  | 0.766 |  |
|  |  |  |  | - |  | 10 |  | BY |  | -0.12153 |  | 0.2193 |  | 7.00 |  | -0.5540 |  | 0.597 |  |
|  |  | BY |  | - |  | 5 |  | A |  | -0.72917 |  | 0.3542 |  | 7.00 |  | -2.0584 |  | 0.079 |  |
|  |  |  |  | - |  | 5 |  | AX |  | -1.17361 |  | 0.4489 |  | 7.00 |  | -2.6144 |  | 0.035 |  |
|  |  |  |  | - |  | 5 |  | B |  | 1.21875 |  | 0.3052 |  | 7.00 |  | 3.9937 |  | 0.005 |  |
|  |  |  |  | - |  | 5 |  | BY |  | 0.45139 |  | 0.1735 |  | 7.00 |  | 2.6012 |  | 0.035 |  |
|  |  |  |  | - |  | 6 |  | A |  | -1.19792 |  | 0.5907 |  | 7.00 |  | -2.0280 |  | 0.082 |  |
|  |  |  |  | - |  | 6 |  | AX |  | -1.40292 |  | 0.6153 |  | 7.00 |  | -2.2801 |  | 0.057 |  |
|  |  |  |  | - |  | 6 |  | B |  | 1.46875 |  | 0.2342 |  | 7.00 |  | 6.2713 |  | < .001 |  |
|  |  |  |  | - |  | 6 |  | BY |  | 0.65278 |  | 0.2132 |  | 7.00 |  | 3.0613 |  | 0.018 |  |
|  |  |  |  | - |  | 7 |  | A |  | -1.02083 |  | 0.5636 |  | 7.00 |  | -1.8113 |  | 0.113 |  |
|  |  |  |  | - |  | 7 |  | AX |  | -1.11111 |  | 0.6242 |  | 7.00 |  | -1.7800 |  | 0.118 |  |
|  |  |  |  | - |  | 7 |  | B |  | 1.64583 |  | 0.3343 |  | 7.00 |  | 4.9230 |  | 0.002 |  |
|  |  |  |  | - |  | 7 |  | BY |  | 0.92708 |  | 0.1890 |  | 7.00 |  | 4.9058 |  | 0.002 |  |
|  |  |  |  | - |  | 8 |  | A |  | -1.62500 |  | 0.9780 |  | 7.00 |  | -1.6616 |  | 0.141 |  |
|  |  |  |  | - |  | 8 |  | AX |  | -1.42361 |  | 0.8467 |  | 7.00 |  | -1.6813 |  | 0.137 |  |
|  |  |  |  | - |  | 8 |  | B |  | 1.72917 |  | 0.2534 |  | 7.00 |  | 6.8229 |  | < .001 |  |
|  |  |  |  | - |  | 8 |  | BY |  | 1.10764 |  | 0.1588 |  | 7.00 |  | 6.9737 |  | < .001 |  |
|  |  |  |  | - |  | 9 |  | A |  | -1.48958 |  | 1.1142 |  | 7.00 |  | -1.3369 |  | 0.223 |  |
|  |  |  |  | - |  | 9 |  | AX |  | -1.31597 |  | 0.8583 |  | 7.00 |  | -1.5332 |  | 0.169 |  |
|  |  |  |  | - |  | 9 |  | B |  | 1.72917 |  | 0.3783 |  | 7.00 |  | 4.5711 |  | 0.003 |  |
|  |  |  |  | - |  | 9 |  | BY |  | 1.42361 |  | 0.3312 |  | 7.00 |  | 4.2988 |  | 0.004 |  |
|  |  |  |  | - |  | 10 |  | A |  | -1.53125 |  | 0.8691 |  | 7.00 |  | -1.7619 |  | 0.121 |  |
|  |  |  |  | - |  | 10 |  | AX |  | -1.10417 |  | 0.6583 |  | 7.00 |  | -1.6774 |  | 0.137 |  |
|  |  |  |  | - |  | 10 |  | B |  | 1.62500 |  | 0.2766 |  | 7.00 |  | 5.8742 |  | < .001 |  |
|  |  |  |  | - |  | 10 |  | BY |  | 1.40972 |  | 0.2396 |  | 7.00 |  | 5.8843 |  | < .001 |  |
| 5 |  | A |  | - |  | 5 |  | AX |  | -0.44444 |  | 0.2479 |  | 7.00 |  | -1.7929 |  | 0.116 |  |
|  |  |  |  | - |  | 5 |  | B |  | 1.94792 |  | 0.4327 |  | 7.00 |  | 4.5017 |  | 0.003 |  |
|  |  |  |  | - |  | 5 |  | BY |  | 1.18056 |  | 0.3117 |  | 7.00 |  | 3.7873 |  | 0.007 |  |
|  |  |  |  | - |  | 6 |  | A |  | -0.46875 |  | 0.3740 |  | 7.00 |  | -1.2534 |  | 0.250 |  |
|  |  |  |  | - |  | 6 |  | AX |  | -0.67375 |  | 0.4496 |  | 7.00 |  | -1.4987 |  | 0.178 |  |
|  |  |  |  | - |  | 6 |  | B |  | 2.19792 |  | 0.4690 |  | 7.00 |  | 4.6862 |  | 0.002 |  |
|  |  |  |  | - |  | 6 |  | BY |  | 1.38194 |  | 0.3321 |  | 7.00 |  | 4.1615 |  | 0.004 |  |
|  |  |  |  | - |  | 7 |  | A |  | -0.29167 |  | 0.4270 |  | 7.00 |  | -0.6831 |  | 0.516 |  |
|  |  |  |  | - |  | 7 |  | AX |  | -0.38194 |  | 0.4836 |  | 7.00 |  | -0.7897 |  | 0.456 |  |
|  |  |  |  | - |  | 7 |  | B |  | 2.37500 |  | 0.5494 |  | 7.00 |  | 4.3229 |  | 0.003 |  |
|  |  |  |  | - |  | 7 |  | BY |  | 1.65625 |  | 0.4438 |  | 7.00 |  | 3.7320 |  | 0.007 |  |
|  |  |  |  | - |  | 8 |  | A |  | -0.89583 |  | 0.8562 |  | 7.00 |  | -1.0463 |  | 0.330 |  |
|  |  |  |  | - |  | 8 |  | AX |  | -0.69444 |  | 0.7157 |  | 7.00 |  | -0.9703 |  | 0.364 |  |
|  |  |  |  | - |  | 8 |  | B |  | 2.45833 |  | 0.4645 |  | 7.00 |  | 5.2923 |  | 0.001 |  |
|  |  |  |  | - |  | 8 |  | BY |  | 1.83681 |  | 0.4483 |  | 7.00 |  | 4.0977 |  | 0.005 |  |
|  |  |  |  | - |  | 9 |  | A |  | -0.76042 |  | 1.0498 |  | 7.00 |  | -0.7244 |  | 0.492 |  |
|  |  |  |  | - |  | 9 |  | AX |  | -0.58681 |  | 0.7754 |  | 7.00 |  | -0.7568 |  | 0.474 |  |
|  |  |  |  | - |  | 9 |  | B |  | 2.45833 |  | 0.6136 |  | 7.00 |  | 4.0065 |  | 0.005 |  |
|  |  |  |  | - |  | 9 |  | BY |  | 2.15278 |  | 0.5862 |  | 7.00 |  | 3.6722 |  | 0.008 |  |
|  |  |  |  | - |  | 10 |  | A |  | -0.80208 |  | 0.8266 |  | 7.00 |  | -0.9703 |  | 0.364 |  |
|  |  |  |  | - |  | 10 |  | AX |  | -0.37500 |  | 0.5980 |  | 7.00 |  | -0.6271 |  | 0.550 |  |
|  |  |  |  | - |  | 10 |  | B |  | 2.35417 |  | 0.4794 |  | 7.00 |  | 4.9104 |  | 0.002 |  |
|  |  |  |  | - |  | 10 |  | BY |  | 2.13889 |  | 0.4765 |  | 7.00 |  | 4.4886 |  | 0.003 |  |
|  |  | AX |  | - |  | 5 |  | B |  | 2.39236 |  | 0.5139 |  | 7.00 |  | 4.6555 |  | 0.002 |  |
|  |  |  |  | - |  | 5 |  | BY |  | 1.62500 |  | 0.3363 |  | 7.00 |  | 4.8315 |  | 0.002 |  |
|  |  |  |  | - |  | 6 |  | A |  | -0.02431 |  | 0.2123 |  | 7.00 |  | -0.1145 |  | 0.912 |  |
|  |  |  |  | - |  | 6 |  | AX |  | -0.22931 |  | 0.2310 |  | 7.00 |  | -0.9925 |  | 0.354 |  |
|  |  |  |  | - |  | 6 |  | B |  | 2.64236 |  | 0.5264 |  | 7.00 |  | 5.0201 |  | 0.002 |  |
|  |  |  |  | - |  | 6 |  | BY |  | 1.82639 |  | 0.4081 |  | 7.00 |  | 4.4753 |  | 0.003 |  |
|  |  |  |  | - |  | 7 |  | A |  | 0.15278 |  | 0.2259 |  | 7.00 |  | 0.6763 |  | 0.521 |  |
|  |  |  |  | - |  | 7 |  | AX |  | 0.06250 |  | 0.2469 |  | 7.00 |  | 0.2532 |  | 0.807 |  |
|  |  |  |  | - |  | 7 |  | B |  | 2.81944 |  | 0.6077 |  | 7.00 |  | 4.6394 |  | 0.002 |  |
|  |  |  |  | - |  | 7 |  | BY |  | 2.10069 |  | 0.5253 |  | 7.00 |  | 3.9987 |  | 0.005 |  |
|  |  |  |  | - |  | 8 |  | A |  | -0.45139 |  | 0.6225 |  | 7.00 |  | -0.7252 |  | 0.492 |  |
|  |  |  |  | - |  | 8 |  | AX |  | -0.25000 |  | 0.4785 |  | 7.00 |  | -0.5224 |  | 0.617 |  |
|  |  |  |  | - |  | 8 |  | B |  | 2.90278 |  | 0.5481 |  | 7.00 |  | 5.2964 |  | 0.001 |  |
|  |  |  |  | - |  | 8 |  | BY |  | 2.28125 |  | 0.5136 |  | 7.00 |  | 4.4414 |  | 0.003 |  |
|  |  |  |  | - |  | 9 |  | A |  | -0.31597 |  | 0.8193 |  | 7.00 |  | -0.3857 |  | 0.711 |  |
|  |  |  |  | - |  | 9 |  | AX |  | -0.14236 |  | 0.5357 |  | 7.00 |  | -0.2657 |  | 0.798 |  |
|  |  |  |  | - |  | 9 |  | B |  | 2.90278 |  | 0.6679 |  | 7.00 |  | 4.3460 |  | 0.003 |  |
|  |  |  |  | - |  | 9 |  | BY |  | 2.59722 |  | 0.6695 |  | 7.00 |  | 3.8796 |  | 0.006 |  |
|  |  |  |  | - |  | 10 |  | A |  | -0.35764 |  | 0.5918 |  | 7.00 |  | -0.6043 |  | 0.565 |  |
|  |  |  |  | - |  | 10 |  | AX |  | 0.06944 |  | 0.3777 |  | 7.00 |  | 0.1839 |  | 0.859 |  |
|  |  |  |  | - |  | 10 |  | B |  | 2.79861 |  | 0.5705 |  | 7.00 |  | 4.9057 |  | 0.002 |  |
|  |  |  |  | - |  | 10 |  | BY |  | 2.58333 |  | 0.5382 |  | 7.00 |  | 4.7998 |  | 0.002 |  |
|  |  | B |  | - |  | 5 |  | BY |  | -0.76736 |  | 0.2545 |  | 7.00 |  | -3.0148 |  | 0.020 |  |
|  |  |  |  | - |  | 6 |  | A |  | -2.41667 |  | 0.6018 |  | 7.00 |  | -4.0161 |  | 0.005 |  |
|  |  |  |  | - |  | 6 |  | AX |  | -2.62167 |  | 0.6600 |  | 7.00 |  | -3.9725 |  | 0.005 |  |
|  |  |  |  | - |  | 6 |  | B |  | 0.25000 |  | 0.1725 |  | 7.00 |  | 1.4491 |  | 0.191 |  |
|  |  |  |  | - |  | 6 |  | BY |  | -0.56597 |  | 0.2282 |  | 7.00 |  | -2.4800 |  | 0.042 |  |
|  |  |  |  | - |  | 7 |  | A |  | -2.23958 |  | 0.5540 |  | 7.00 |  | -4.0426 |  | 0.005 |  |
|  |  |  |  | - |  | 7 |  | AX |  | -2.32986 |  | 0.6491 |  | 7.00 |  | -3.5892 |  | 0.009 |  |
|  |  |  |  | - |  | 7 |  | B |  | 0.42708 |  | 0.2019 |  | 7.00 |  | 2.1148 |  | 0.072 |  |
|  |  |  |  | - |  | 7 |  | BY |  | -0.29167 |  | 0.1881 |  | 7.00 |  | -1.5506 |  | 0.165 |  |
|  |  |  |  | - |  | 8 |  | A |  | -2.84375 |  | 0.9765 |  | 7.00 |  | -2.9123 |  | 0.023 |  |
|  |  |  |  | - |  | 8 |  | AX |  | -2.64236 |  | 0.8726 |  | 7.00 |  | -3.0282 |  | 0.019 |  |
|  |  |  |  | - |  | 8 |  | B |  | 0.51042 |  | 0.2168 |  | 7.00 |  | 2.3548 |  | 0.051 |  |
|  |  |  |  | - |  | 8 |  | BY |  | -0.11111 |  | 0.2410 |  | 7.00 |  | -0.4610 |  | 0.659 |  |
|  |  |  |  | - |  | 9 |  | A |  | -2.70833 |  | 1.0747 |  | 7.00 |  | -2.5201 |  | 0.040 |  |
|  |  |  |  | - |  | 9 |  | AX |  | -2.53472 |  | 0.8582 |  | 7.00 |  | -2.9535 |  | 0.021 |  |
|  |  |  |  | - |  | 9 |  | B |  | 0.51042 |  | 0.3002 |  | 7.00 |  | 1.7001 |  | 0.133 |  |
|  |  |  |  | - |  | 9 |  | BY |  | 0.20486 |  | 0.2497 |  | 7.00 |  | 0.8204 |  | 0.439 |  |
|  |  |  |  | - |  | 10 |  | A |  | -2.75000 |  | 0.8881 |  | 7.00 |  | -3.0966 |  | 0.017 |  |
|  |  |  |  | - |  | 10 |  | AX |  | -2.32292 |  | 0.6710 |  | 7.00 |  | -3.4617 |  | 0.011 |  |
|  |  |  |  | - |  | 10 |  | B |  | 0.40625 |  | 0.2354 |  | 7.00 |  | 1.7257 |  | 0.128 |  |
|  |  |  |  | - |  | 10 |  | BY |  | 0.19097 |  | 0.2301 |  | 7.00 |  | 0.8299 |  | 0.434 |  |
|  |  | BY |  | - |  | 6 |  | A |  | -1.64931 |  | 0.4617 |  | 7.00 |  | -3.5721 |  | 0.009 |  |
|  |  |  |  | - |  | 6 |  | AX |  | -1.85431 |  | 0.4892 |  | 7.00 |  | -3.7906 |  | 0.007 |  |
|  |  |  |  | - |  | 6 |  | B |  | 1.01736 |  | 0.2162 |  | 7.00 |  | 4.7046 |  | 0.002 |  |
|  |  |  |  | - |  | 6 |  | BY |  | 0.20139 |  | 0.1773 |  | 7.00 |  | 1.1359 |  | 0.293 |  |
|  |  |  |  | - |  | 7 |  | A |  | -1.47222 |  | 0.4107 |  | 7.00 |  | -3.5846 |  | 0.009 |  |
|  |  |  |  | - |  | 7 |  | AX |  | -1.56250 |  | 0.4821 |  | 7.00 |  | -3.2413 |  | 0.014 |  |
|  |  |  |  | - |  | 7 |  | B |  | 1.19444 |  | 0.3252 |  | 7.00 |  | 3.6728 |  | 0.008 |  |
|  |  |  |  | - |  | 7 |  | BY |  | 0.47569 |  | 0.2306 |  | 7.00 |  | 2.0624 |  | 0.078 |  |
|  |  |  |  | - |  | 8 |  | A |  | -2.07639 |  | 0.8248 |  | 7.00 |  | -2.5175 |  | 0.040 |  |
|  |  |  |  | - |  | 8 |  | AX |  | -1.87500 |  | 0.7036 |  | 7.00 |  | -2.6650 |  | 0.032 |  |
|  |  |  |  | - |  | 8 |  | B |  | 1.27778 |  | 0.2763 |  | 7.00 |  | 4.6248 |  | 0.002 |  |
|  |  |  |  | - |  | 8 |  | BY |  | 0.65625 |  | 0.2099 |  | 7.00 |  | 3.1263 |  | 0.017 |  |
|  |  |  |  | - |  | 9 |  | A |  | -1.94097 |  | 0.9607 |  | 7.00 |  | -2.0204 |  | 0.083 |  |
|  |  |  |  | - |  | 9 |  | AX |  | -1.76736 |  | 0.7087 |  | 7.00 |  | -2.4939 |  | 0.041 |  |
|  |  |  |  | - |  | 9 |  | B |  | 1.27778 |  | 0.4060 |  | 7.00 |  | 3.1469 |  | 0.016 |  |
|  |  |  |  | - |  | 9 |  | BY |  | 0.97222 |  | 0.3771 |  | 7.00 |  | 2.5782 |  | 0.037 |  |
|  |  |  |  | - |  | 10 |  | A |  | -1.98264 |  | 0.7362 |  | 7.00 |  | -2.6931 |  | 0.031 |  |
|  |  |  |  | - |  | 10 |  | AX |  | -1.55556 |  | 0.5243 |  | 7.00 |  | -2.9671 |  | 0.021 |  |
|  |  |  |  | - |  | 10 |  | B |  | 1.17361 |  | 0.3254 |  | 7.00 |  | 3.6070 |  | 0.009 |  |
|  |  |  |  | - |  | 10 |  | BY |  | 0.95833 |  | 0.2658 |  | 7.00 |  | 3.6060 |  | 0.009 |  |
| 6 |  | A |  | - |  | 6 |  | AX |  | -0.20500 |  | 0.1467 |  | 7.00 |  | -1.3977 |  | 0.205 |  |
|  |  |  |  | - |  | 6 |  | B |  | 2.66667 |  | 0.6352 |  | 7.00 |  | 4.1979 |  | 0.004 |  |
|  |  |  |  | - |  | 6 |  | BY |  | 1.85069 |  | 0.5139 |  | 7.00 |  | 3.6010 |  | 0.009 |  |
|  |  |  |  | - |  | 7 |  | A |  | 0.17708 |  | 0.1555 |  | 7.00 |  | 1.1391 |  | 0.292 |  |
|  |  |  |  | - |  | 7 |  | AX |  | 0.08681 |  | 0.2629 |  | 7.00 |  | 0.3302 |  | 0.751 |  |
|  |  |  |  | - |  | 7 |  | B |  | 2.84375 |  | 0.7128 |  | 7.00 |  | 3.9897 |  | 0.005 |  |
|  |  |  |  | - |  | 7 |  | BY |  | 2.12500 |  | 0.6529 |  | 7.00 |  | 3.2548 |  | 0.014 |  |
|  |  |  |  | - |  | 8 |  | A |  | -0.42708 |  | 0.5517 |  | 7.00 |  | -0.7741 |  | 0.464 |  |
|  |  |  |  | - |  | 8 |  | AX |  | -0.22569 |  | 0.4035 |  | 7.00 |  | -0.5594 |  | 0.593 |  |
|  |  |  |  | - |  | 8 |  | B |  | 2.92708 |  | 0.6589 |  | 7.00 |  | 4.4424 |  | 0.003 |  |
|  |  |  |  | - |  | 8 |  | BY |  | 2.30556 |  | 0.6553 |  | 7.00 |  | 3.5185 |  | 0.010 |  |
|  |  |  |  | - |  | 9 |  | A |  | -0.29167 |  | 0.7849 |  | 7.00 |  | -0.3716 |  | 0.721 |  |
|  |  |  |  | - |  | 9 |  | AX |  | -0.11806 |  | 0.5037 |  | 7.00 |  | -0.2344 |  | 0.821 |  |
|  |  |  |  | - |  | 9 |  | B |  | 2.92708 |  | 0.7913 |  | 7.00 |  | 3.6992 |  | 0.008 |  |
|  |  |  |  | - |  | 9 |  | BY |  | 2.62153 |  | 0.7884 |  | 7.00 |  | 3.3252 |  | 0.013 |  |
|  |  |  |  | - |  | 10 |  | A |  | -0.33333 |  | 0.5984 |  | 7.00 |  | -0.5570 |  | 0.595 |  |
|  |  |  |  | - |  | 10 |  | AX |  | 0.09375 |  | 0.4219 |  | 7.00 |  | 0.2222 |  | 0.830 |  |
|  |  |  |  | - |  | 10 |  | B |  | 2.82292 |  | 0.7000 |  | 7.00 |  | 4.0330 |  | 0.005 |  |
|  |  |  |  | - |  | 10 |  | BY |  | 2.60764 |  | 0.6677 |  | 7.00 |  | 3.9055 |  | 0.006 |  |
|  |  | AX |  | - |  | 6 |  | B |  | 2.87167 |  | 0.6701 |  | 7.00 |  | 4.2855 |  | 0.004 |  |
|  |  |  |  | - |  | 6 |  | BY |  | 2.05569 |  | 0.5512 |  | 7.00 |  | 3.7295 |  | 0.007 |  |
|  |  |  |  | - |  | 7 |  | A |  | 0.38208 |  | 0.1559 |  | 7.00 |  | 2.4504 |  | 0.044 |  |
|  |  |  |  | - |  | 7 |  | AX |  | 0.29181 |  | 0.1845 |  | 7.00 |  | 1.5812 |  | 0.158 |  |
|  |  |  |  | - |  | 7 |  | B |  | 3.04875 |  | 0.7509 |  | 7.00 |  | 4.0599 |  | 0.005 |  |
|  |  |  |  | - |  | 7 |  | BY |  | 2.33000 |  | 0.6833 |  | 7.00 |  | 3.4100 |  | 0.011 |  |
|  |  |  |  | - |  | 8 |  | A |  | -0.22208 |  | 0.4725 |  | 7.00 |  | -0.4700 |  | 0.653 |  |
|  |  |  |  | - |  | 8 |  | AX |  | -0.02069 |  | 0.3055 |  | 7.00 |  | -0.0677 |  | 0.948 |  |
|  |  |  |  | - |  | 8 |  | B |  | 3.13208 |  | 0.7029 |  | 7.00 |  | 4.4560 |  | 0.003 |  |
|  |  |  |  | - |  | 8 |  | BY |  | 2.51056 |  | 0.6733 |  | 7.00 |  | 3.7288 |  | 0.007 |  |
|  |  |  |  | - |  | 9 |  | A |  | -0.08667 |  | 0.7076 |  | 7.00 |  | -0.1225 |  | 0.906 |  |
|  |  |  |  | - |  | 9 |  | AX |  | 0.08694 |  | 0.4079 |  | 7.00 |  | 0.2131 |  | 0.837 |  |
|  |  |  |  | - |  | 9 |  | B |  | 3.13208 |  | 0.8115 |  | 7.00 |  | 3.8597 |  | 0.006 |  |
|  |  |  |  | - |  | 9 |  | BY |  | 2.82653 |  | 0.8218 |  | 7.00 |  | 3.4394 |  | 0.011 |  |
|  |  |  |  | - |  | 10 |  | A |  | -0.12833 |  | 0.4938 |  | 7.00 |  | -0.2599 |  | 0.802 |  |
|  |  |  |  | - |  | 10 |  | AX |  | 0.29875 |  | 0.3426 |  | 7.00 |  | 0.8720 |  | 0.412 |  |
|  |  |  |  | - |  | 10 |  | B |  | 3.02792 |  | 0.7332 |  | 7.00 |  | 4.1299 |  | 0.004 |  |
|  |  |  |  | - |  | 10 |  | BY |  | 2.81264 |  | 0.6945 |  | 7.00 |  | 4.0499 |  | 0.005 |  |
|  |  | B |  | - |  | 6 |  | BY |  | -0.81597 |  | 0.2500 |  | 7.00 |  | -3.2639 |  | 0.014 |  |
|  |  |  |  | - |  | 7 |  | A |  | -2.48958 |  | 0.5729 |  | 7.00 |  | -4.3455 |  | 0.003 |  |
|  |  |  |  | - |  | 7 |  | AX |  | -2.57986 |  | 0.6550 |  | 7.00 |  | -3.9386 |  | 0.006 |  |
|  |  |  |  | - |  | 7 |  | B |  | 0.17708 |  | 0.1303 |  | 7.00 |  | 1.3592 |  | 0.216 |  |
|  |  |  |  | - |  | 7 |  | BY |  | -0.54167 |  | 0.1629 |  | 7.00 |  | -3.3251 |  | 0.013 |  |
|  |  |  |  | - |  | 8 |  | A |  | -3.09375 |  | 0.9712 |  | 7.00 |  | -3.1854 |  | 0.015 |  |
|  |  |  |  | - |  | 8 |  | AX |  | -2.89236 |  | 0.8636 |  | 7.00 |  | -3.3490 |  | 0.012 |  |
|  |  |  |  | - |  | 8 |  | B |  | 0.26042 |  | 0.1632 |  | 7.00 |  | 1.5953 |  | 0.155 |  |
|  |  |  |  | - |  | 8 |  | BY |  | -0.36111 |  | 0.1479 |  | 7.00 |  | -2.4412 |  | 0.045 |  |
|  |  |  |  | - |  | 9 |  | A |  | -2.95833 |  | 1.0595 |  | 7.00 |  | -2.7922 |  | 0.027 |  |
|  |  |  |  | - |  | 9 |  | AX |  | -2.78472 |  | 0.8433 |  | 7.00 |  | -3.3023 |  | 0.013 |  |
|  |  |  |  | - |  | 9 |  | B |  | 0.26042 |  | 0.2385 |  | 7.00 |  | 1.0917 |  | 0.311 |  |
|  |  |  |  | - |  | 9 |  | BY |  | -0.04514 |  | 0.2125 |  | 7.00 |  | -0.2124 |  | 0.838 |  |
|  |  |  |  | - |  | 10 |  | A |  | -3.00000 |  | 0.8465 |  | 7.00 |  | -3.5441 |  | 0.009 |  |
|  |  |  |  | - |  | 10 |  | AX |  | -2.57292 |  | 0.6401 |  | 7.00 |  | -4.0197 |  | 0.005 |  |
|  |  |  |  | - |  | 10 |  | B |  | 0.15625 |  | 0.2168 |  | 7.00 |  | 0.7209 |  | 0.494 |  |
|  |  |  |  | - |  | 10 |  | BY |  | -0.05903 |  | 0.1722 |  | 7.00 |  | -0.3429 |  | 0.742 |  |
|  |  | BY |  | - |  | 7 |  | A |  | -1.67361 |  | 0.4853 |  | 7.00 |  | -3.4485 |  | 0.011 |  |
|  |  |  |  | - |  | 7 |  | AX |  | -1.76389 |  | 0.5525 |  | 7.00 |  | -3.1926 |  | 0.015 |  |
|  |  |  |  | - |  | 7 |  | B |  | 0.99306 |  | 0.3537 |  | 7.00 |  | 2.8079 |  | 0.026 |  |
|  |  |  |  | - |  | 7 |  | BY |  | 0.27431 |  | 0.2297 |  | 7.00 |  | 1.1943 |  | 0.271 |  |
|  |  |  |  | - |  | 8 |  | A |  | -2.27778 |  | 0.9129 |  | 7.00 |  | -2.4950 |  | 0.041 |  |
|  |  |  |  | - |  | 8 |  | AX |  | -2.07639 |  | 0.7853 |  | 7.00 |  | -2.6441 |  | 0.033 |  |
|  |  |  |  | - |  | 8 |  | B |  | 1.07639 |  | 0.3291 |  | 7.00 |  | 3.2709 |  | 0.014 |  |
|  |  |  |  | - |  | 8 |  | BY |  | 0.45486 |  | 0.2171 |  | 7.00 |  | 2.0948 |  | 0.074 |  |
|  |  |  |  | - |  | 9 |  | A |  | -2.14236 |  | 1.0596 |  | 7.00 |  | -2.0219 |  | 0.083 |  |
|  |  |  |  | - |  | 9 |  | AX |  | -1.96875 |  | 0.7985 |  | 7.00 |  | -2.4655 |  | 0.043 |  |
|  |  |  |  | - |  | 9 |  | B |  | 1.07639 |  | 0.3944 |  | 7.00 |  | 2.7289 |  | 0.029 |  |
|  |  |  |  | - |  | 9 |  | BY |  | 0.77083 |  | 0.3762 |  | 7.00 |  | 2.0490 |  | 0.080 |  |
|  |  |  |  | - |  | 10 |  | A |  | -2.18403 |  | 0.8338 |  | 7.00 |  | -2.6194 |  | 0.034 |  |
|  |  |  |  | - |  | 10 |  | AX |  | -1.75694 |  | 0.6156 |  | 7.00 |  | -2.8543 |  | 0.025 |  |
|  |  |  |  | - |  | 10 |  | B |  | 0.97222 |  | 0.2978 |  | 7.00 |  | 3.2648 |  | 0.014 |  |
|  |  |  |  | - |  | 10 |  | BY |  | 0.75694 |  | 0.3139 |  | 7.00 |  | 2.4114 |  | 0.047 |  |
| 7 |  | A |  | - |  | 7 |  | AX |  | -0.09028 |  | 0.1830 |  | 7.00 |  | -0.4932 |  | 0.637 |  |
|  |  |  |  | - |  | 7 |  | B |  | 2.66667 |  | 0.6466 |  | 7.00 |  | 4.1238 |  | 0.004 |  |
|  |  |  |  | - |  | 7 |  | BY |  | 1.94792 |  | 0.6049 |  | 7.00 |  | 3.2203 |  | 0.015 |  |
|  |  |  |  | - |  | 8 |  | A |  | -0.60417 |  | 0.4732 |  | 7.00 |  | -1.2768 |  | 0.242 |  |
|  |  |  |  | - |  | 8 |  | AX |  | -0.40278 |  | 0.3415 |  | 7.00 |  | -1.1796 |  | 0.277 |  |
|  |  |  |  | - |  | 8 |  | B |  | 2.75000 |  | 0.6148 |  | 7.00 |  | 4.4730 |  | 0.003 |  |
|  |  |  |  | - |  | 8 |  | BY |  | 2.12847 |  | 0.6000 |  | 7.00 |  | 3.5477 |  | 0.009 |  |
|  |  |  |  | - |  | 9 |  | A |  | -0.46875 |  | 0.6711 |  | 7.00 |  | -0.6985 |  | 0.507 |  |
|  |  |  |  | - |  | 9 |  | AX |  | -0.29514 |  | 0.3940 |  | 7.00 |  | -0.7492 |  | 0.478 |  |
|  |  |  |  | - |  | 9 |  | B |  | 2.75000 |  | 0.7258 |  | 7.00 |  | 3.7889 |  | 0.007 |  |
|  |  |  |  | - |  | 9 |  | BY |  | 2.44444 |  | 0.7316 |  | 7.00 |  | 3.3410 |  | 0.012 |  |
|  |  |  |  | - |  | 10 |  | A |  | -0.51042 |  | 0.4860 |  | 7.00 |  | -1.0502 |  | 0.329 |  |
|  |  |  |  | - |  | 10 |  | AX |  | -0.08333 |  | 0.3105 |  | 7.00 |  | -0.2684 |  | 0.796 |  |
|  |  |  |  | - |  | 10 |  | B |  | 2.64583 |  | 0.6603 |  | 7.00 |  | 4.0072 |  | 0.005 |  |
|  |  |  |  | - |  | 10 |  | BY |  | 2.43056 |  | 0.6122 |  | 7.00 |  | 3.9701 |  | 0.005 |  |
|  |  | AX |  | - |  | 7 |  | B |  | 2.75694 |  | 0.7327 |  | 7.00 |  | 3.7629 |  | 0.007 |  |
|  |  |  |  | - |  | 7 |  | BY |  | 2.03819 |  | 0.6766 |  | 7.00 |  | 3.0125 |  | 0.020 |  |
|  |  |  |  | - |  | 8 |  | A |  | -0.51389 |  | 0.3967 |  | 7.00 |  | -1.2953 |  | 0.236 |  |
|  |  |  |  | - |  | 8 |  | AX |  | -0.31250 |  | 0.2681 |  | 7.00 |  | -1.1655 |  | 0.282 |  |
|  |  |  |  | - |  | 8 |  | B |  | 2.84028 |  | 0.7099 |  | 7.00 |  | 4.0012 |  | 0.005 |  |
|  |  |  |  | - |  | 8 |  | BY |  | 2.21875 |  | 0.6522 |  | 7.00 |  | 3.4021 |  | 0.011 |  |
|  |  |  |  | - |  | 9 |  | A |  | -0.37847 |  | 0.6041 |  | 7.00 |  | -0.6265 |  | 0.551 |  |
|  |  |  |  | - |  | 9 |  | AX |  | -0.20486 |  | 0.3079 |  | 7.00 |  | -0.6654 |  | 0.527 |  |
|  |  |  |  | - |  | 9 |  | B |  | 2.84028 |  | 0.7876 |  | 7.00 |  | 3.6063 |  | 0.009 |  |
|  |  |  |  | - |  | 9 |  | BY |  | 2.53472 |  | 0.8101 |  | 7.00 |  | 3.1287 |  | 0.017 |  |
|  |  |  |  | - |  | 10 |  | A |  | -0.42014 |  | 0.3998 |  | 7.00 |  | -1.0508 |  | 0.328 |  |
|  |  |  |  | - |  | 10 |  | AX |  | 0.00694 |  | 0.2455 |  | 7.00 |  | 0.0283 |  | 0.978 |  |
|  |  |  |  | - |  | 10 |  | B |  | 2.73611 |  | 0.7282 |  | 7.00 |  | 3.7574 |  | 0.007 |  |
|  |  |  |  | - |  | 10 |  | BY |  | 2.52083 |  | 0.6865 |  | 7.00 |  | 3.6719 |  | 0.008 |  |
|  |  | B |  | - |  | 7 |  | BY |  | -0.71875 |  | 0.2091 |  | 7.00 |  | -3.4375 |  | 0.011 |  |
|  |  |  |  | - |  | 8 |  | A |  | -3.27083 |  | 1.0366 |  | 7.00 |  | -3.1553 |  | 0.016 |  |
|  |  |  |  | - |  | 8 |  | AX |  | -3.06944 |  | 0.9375 |  | 7.00 |  | -3.2741 |  | 0.014 |  |
|  |  |  |  | - |  | 8 |  | B |  | 0.08333 |  | 0.1511 |  | 7.00 |  | 0.5517 |  | 0.598 |  |
|  |  |  |  | - |  | 8 |  | BY |  | -0.53819 |  | 0.2311 |  | 7.00 |  | -2.3289 |  | 0.053 |  |
|  |  |  |  | - |  | 9 |  | A |  | -3.13542 |  | 1.0938 |  | 7.00 |  | -2.8665 |  | 0.024 |  |
|  |  |  |  | - |  | 9 |  | AX |  | -2.96181 |  | 0.9025 |  | 7.00 |  | -3.2819 |  | 0.013 |  |
|  |  |  |  | - |  | 9 |  | B |  | 0.08333 |  | 0.1718 |  | 7.00 |  | 0.4851 |  | 0.642 |  |
|  |  |  |  | - |  | 9 |  | BY |  | -0.22222 |  | 0.1516 |  | 7.00 |  | -1.4658 |  | 0.186 |  |
|  |  |  |  | - |  | 10 |  | A |  | -3.17708 |  | 0.8941 |  | 7.00 |  | -3.5533 |  | 0.009 |  |
|  |  |  |  | - |  | 10 |  | AX |  | -2.75000 |  | 0.6906 |  | 7.00 |  | -3.9819 |  | 0.005 |  |
|  |  |  |  | - |  | 10 |  | B |  | -0.02083 |  | 0.2015 |  | 7.00 |  | -0.1034 |  | 0.921 |  |
|  |  |  |  | - |  | 10 |  | BY |  | -0.23611 |  | 0.1658 |  | 7.00 |  | -1.4237 |  | 0.198 |  |
|  |  | BY |  | - |  | 8 |  | A |  | -2.55208 |  | 1.0210 |  | 7.00 |  | -2.4996 |  | 0.041 |  |
|  |  |  |  | - |  | 8 |  | AX |  | -2.35069 |  | 0.9054 |  | 7.00 |  | -2.5962 |  | 0.036 |  |
|  |  |  |  | - |  | 8 |  | B |  | 0.80208 |  | 0.1651 |  | 7.00 |  | 4.8574 |  | 0.002 |  |
|  |  |  |  | - |  | 8 |  | BY |  | 0.18056 |  | 0.1150 |  | 7.00 |  | 1.5699 |  | 0.160 |  |
|  |  |  |  | - |  | 9 |  | A |  | -2.41667 |  | 1.1173 |  | 7.00 |  | -2.1630 |  | 0.067 |  |
|  |  |  |  | - |  | 9 |  | AX |  | -2.24306 |  | 0.8892 |  | 7.00 |  | -2.5225 |  | 0.040 |  |
|  |  |  |  | - |  | 9 |  | B |  | 0.80208 |  | 0.2421 |  | 7.00 |  | 3.3134 |  | 0.013 |  |
|  |  |  |  | - |  | 9 |  | BY |  | 0.49653 |  | 0.1703 |  | 7.00 |  | 2.9156 |  | 0.022 |  |
|  |  |  |  | - |  | 10 |  | A |  | -2.45833 |  | 0.8980 |  | 7.00 |  | -2.7375 |  | 0.029 |  |
|  |  |  |  | - |  | 10 |  | AX |  | -2.03125 |  | 0.6865 |  | 7.00 |  | -2.9590 |  | 0.021 |  |
|  |  |  |  | - |  | 10 |  | B |  | 0.69792 |  | 0.1532 |  | 7.00 |  | 4.5563 |  | 0.003 |  |
|  |  |  |  | - |  | 10 |  | BY |  | 0.48264 |  | 0.1133 |  | 7.00 |  | 4.2593 |  | 0.004 |  |
| 8 |  | A |  | - |  | 8 |  | AX |  | 0.20139 |  | 0.1845 |  | 7.00 |  | 1.0918 |  | 0.311 |  |
|  |  |  |  | - |  | 8 |  | B |  | 3.35417 |  | 1.0329 |  | 7.00 |  | 3.2473 |  | 0.014 |  |
|  |  |  |  | - |  | 8 |  | BY |  | 2.73264 |  | 0.9944 |  | 7.00 |  | 2.7480 |  | 0.029 |  |
|  |  |  |  | - |  | 9 |  | A |  | 0.13542 |  | 0.3262 |  | 7.00 |  | 0.4152 |  | 0.690 |  |
|  |  |  |  | - |  | 9 |  | AX |  | 0.30903 |  | 0.1767 |  | 7.00 |  | 1.7486 |  | 0.124 |  |
|  |  |  |  | - |  | 9 |  | B |  | 3.35417 |  | 1.1061 |  | 7.00 |  | 3.0325 |  | 0.019 |  |
|  |  |  |  | - |  | 9 |  | BY |  | 3.04861 |  | 1.1338 |  | 7.00 |  | 2.6888 |  | 0.031 |  |
|  |  |  |  | - |  | 10 |  | A |  | 0.09375 |  | 0.3275 |  | 7.00 |  | 0.2863 |  | 0.783 |  |
|  |  |  |  | - |  | 10 |  | AX |  | 0.52083 |  | 0.4281 |  | 7.00 |  | 1.2167 |  | 0.263 |  |
|  |  |  |  | - |  | 10 |  | B |  | 3.25000 |  | 1.0782 |  | 7.00 |  | 3.0144 |  | 0.020 |  |
|  |  |  |  | - |  | 10 |  | BY |  | 3.03472 |  | 1.0163 |  | 7.00 |  | 2.9860 |  | 0.020 |  |
|  |  | AX |  | - |  | 8 |  | B |  | 3.15278 |  | 0.9193 |  | 7.00 |  | 3.4294 |  | 0.011 |  |
|  |  |  |  | - |  | 8 |  | BY |  | 2.53125 |  | 0.8786 |  | 7.00 |  | 2.8809 |  | 0.024 |  |
|  |  |  |  | - |  | 9 |  | A |  | -0.06597 |  | 0.4614 |  | 7.00 |  | -0.1430 |  | 0.890 |  |
|  |  |  |  | - |  | 9 |  | AX |  | 0.10764 |  | 0.1844 |  | 7.00 |  | 0.5838 |  | 0.578 |  |
|  |  |  |  | - |  | 9 |  | B |  | 3.15278 |  | 1.0010 |  | 7.00 |  | 3.1496 |  | 0.016 |  |
|  |  |  |  | - |  | 9 |  | BY |  | 2.84722 |  | 1.0285 |  | 7.00 |  | 2.7684 |  | 0.028 |  |
|  |  |  |  | - |  | 10 |  | A |  | -0.10764 |  | 0.3151 |  | 7.00 |  | -0.3416 |  | 0.743 |  |
|  |  |  |  | - |  | 10 |  | AX |  | 0.31944 |  | 0.3336 |  | 7.00 |  | 0.9576 |  | 0.370 |  |
|  |  |  |  | - |  | 10 |  | B |  | 3.04861 |  | 0.9583 |  | 7.00 |  | 3.1814 |  | 0.015 |  |
|  |  |  |  | - |  | 10 |  | BY |  | 2.83333 |  | 0.9049 |  | 7.00 |  | 3.1312 |  | 0.017 |  |
|  |  | B |  | - |  | 8 |  | BY |  | -0.62153 |  | 0.2216 |  | 7.00 |  | -2.8048 |  | 0.026 |  |
|  |  |  |  | - |  | 9 |  | A |  | -3.21875 |  | 1.1175 |  | 7.00 |  | -2.8804 |  | 0.024 |  |
|  |  |  |  | - |  | 9 |  | AX |  | -3.04514 |  | 0.9051 |  | 7.00 |  | -3.3644 |  | 0.012 |  |
|  |  |  |  | - |  | 9 |  | B |  | -1.25e−10 |  | 0.2530 |  | 7.00 |  | -4.94e−10 |  | 1.000 |  |
|  |  |  |  | - |  | 9 |  | BY |  | -0.30556 |  | 0.1768 |  | 7.00 |  | -1.7285 |  | 0.128 |  |
|  |  |  |  | - |  | 10 |  | A |  | -3.26042 |  | 0.9042 |  | 7.00 |  | -3.6059 |  | 0.009 |  |
|  |  |  |  | - |  | 10 |  | AX |  | -2.83333 |  | 0.6922 |  | 7.00 |  | -4.0934 |  | 0.005 |  |
|  |  |  |  | - |  | 10 |  | B |  | -0.10417 |  | 0.1875 |  | 7.00 |  | -0.5556 |  | 0.596 |  |
|  |  |  |  | - |  | 10 |  | BY |  | -0.31944 |  | 0.1063 |  | 7.00 |  | -3.0053 |  | 0.020 |  |
|  |  | BY |  | - |  | 9 |  | A |  | -2.59722 |  | 1.0927 |  | 7.00 |  | -2.3770 |  | 0.049 |  |
|  |  |  |  | - |  | 9 |  | AX |  | -2.42361 |  | 0.8606 |  | 7.00 |  | -2.8163 |  | 0.026 |  |
|  |  |  |  | - |  | 9 |  | B |  | 0.62153 |  | 0.2473 |  | 7.00 |  | 2.5129 |  | 0.040 |  |
|  |  |  |  | - |  | 9 |  | BY |  | 0.31597 |  | 0.2271 |  | 7.00 |  | 1.3912 |  | 0.207 |  |
|  |  |  |  | - |  | 10 |  | A |  | -2.63889 |  | 0.8596 |  | 7.00 |  | -3.0699 |  | 0.018 |  |
|  |  |  |  | - |  | 10 |  | AX |  | -2.21181 |  | 0.6522 |  | 7.00 |  | -3.3912 |  | 0.012 |  |
|  |  |  |  | - |  | 10 |  | B |  | 0.51736 |  | 0.1924 |  | 7.00 |  | 2.6883 |  | 0.031 |  |
|  |  |  |  | - |  | 10 |  | BY |  | 0.30208 |  | 0.1701 |  | 7.00 |  | 1.7755 |  | 0.119 |  |
| 9 |  | A |  | - |  | 9 |  | AX |  | 0.17361 |  | 0.3147 |  | 7.00 |  | 0.5516 |  | 0.598 |  |
|  |  |  |  | - |  | 9 |  | B |  | 3.21875 |  | 1.1495 |  | 7.00 |  | 2.8001 |  | 0.027 |  |
|  |  |  |  | - |  | 9 |  | BY |  | 2.91319 |  | 1.1943 |  | 7.00 |  | 2.4393 |  | 0.045 |  |
|  |  |  |  | - |  | 10 |  | A |  | -0.04167 |  | 0.3307 |  | 7.00 |  | -0.1260 |  | 0.903 |  |
|  |  |  |  | - |  | 10 |  | AX |  | 0.38542 |  | 0.5140 |  | 7.00 |  | 0.7499 |  | 0.478 |  |
|  |  |  |  | - |  | 10 |  | B |  | 3.11458 |  | 1.1599 |  | 7.00 |  | 2.6851 |  | 0.031 |  |
|  |  |  |  | - |  | 10 |  | BY |  | 2.89931 |  | 1.0906 |  | 7.00 |  | 2.6584 |  | 0.033 |  |
|  |  | AX |  | - |  | 9 |  | B |  | 3.04514 |  | 0.9574 |  | 7.00 |  | 3.1806 |  | 0.015 |  |
|  |  |  |  | - |  | 9 |  | BY |  | 2.73958 |  | 0.9951 |  | 7.00 |  | 2.7532 |  | 0.028 |  |
|  |  |  |  | - |  | 10 |  | A |  | -0.21528 |  | 0.1862 |  | 7.00 |  | -1.1563 |  | 0.285 |  |
|  |  |  |  | - |  | 10 |  | AX |  | 0.21181 |  | 0.2766 |  | 7.00 |  | 0.7658 |  | 0.469 |  |
|  |  |  |  | - |  | 10 |  | B |  | 2.94097 |  | 0.9393 |  | 7.00 |  | 3.1311 |  | 0.017 |  |
|  |  |  |  | - |  | 10 |  | BY |  | 2.72569 |  | 0.8789 |  | 7.00 |  | 3.1012 |  | 0.017 |  |
|  |  | B |  | - |  | 9 |  | BY |  | -0.30556 |  | 0.1554 |  | 7.00 |  | -1.9666 |  | 0.090 |  |
|  |  |  |  | - |  | 10 |  | A |  | -3.26042 |  | 0.9296 |  | 7.00 |  | -3.5073 |  | 0.010 |  |
|  |  |  |  | - |  | 10 |  | AX |  | -2.83333 |  | 0.7282 |  | 7.00 |  | -3.8908 |  | 0.006 |  |
|  |  |  |  | - |  | 10 |  | B |  | -0.10417 |  | 0.1604 |  | 7.00 |  | -0.6494 |  | 0.537 |  |
|  |  |  |  | - |  | 10 |  | BY |  | -0.31944 |  | 0.2083 |  | 7.00 |  | -1.5338 |  | 0.169 |  |
|  |  | BY |  | - |  | 10 |  | A |  | -2.95486 |  | 0.9873 |  | 7.00 |  | -2.9929 |  | 0.020 |  |
|  |  |  |  | - |  | 10 |  | AX |  | -2.52778 |  | 0.7835 |  | 7.00 |  | -3.2261 |  | 0.015 |  |
|  |  |  |  | - |  | 10 |  | B |  | 0.20139 |  | 0.1635 |  | 7.00 |  | 1.2319 |  | 0.258 |  |
|  |  |  |  | - |  | 10 |  | BY |  | -0.01389 |  | 0.1489 |  | 7.00 |  | -0.0933 |  | 0.928 |  |
| 10 |  | A |  | - |  | 10 |  | AX |  | 0.42708 |  | 0.2451 |  | 7.00 |  | 1.7425 |  | 0.125 |  |
|  |  |  |  | - |  | 10 |  | B |  | 3.15625 |  | 0.9298 |  | 7.00 |  | 3.3947 |  | 0.012 |  |
|  |  |  |  | - |  | 10 |  | BY |  | 2.94097 |  | 0.8719 |  | 7.00 |  | 3.3731 |  | 0.012 |  |
|  |  | AX |  | - |  | 10 |  | B |  | 2.72917 |  | 0.7086 |  | 7.00 |  | 3.8515 |  | 0.006 |  |
|  |  |  |  | - |  | 10 |  | BY |  | 2.51389 |  | 0.6637 |  | 7.00 |  | 3.7878 |  | 0.007 |  |
|  |  | B |  | - |  | 10 |  | BY |  | -0.21528 |  | 0.1497 |  | 7.00 |  | -1.4385 |  | 0.193 |  |
|  | | | | | | | | | | | | | | | | | | | |

**Experiment S2: Ruling out alternatives for the role of agency in competitive credit assignment – Replication**

**Negative patterning discrimination**

| Fixed Effect Omnibus tests | | | | | | | | | |
| --- | --- | --- | --- | --- | --- | --- | --- | --- | --- |
|  |  |  |  |  |  |  |  |  |  |
|  | | **F** | | **Num df** | | **Den df** | | **p** | |
| Group |  | 0.846 |  | 1 |  | 14.0 |  | 0.373 |  |
| 3 Sess Block |  | 1.228 |  | 12 |  | 350.0 |  | 0.262 |  |
| Stimulus |  | 53.373 |  | 1 |  | 350.0 |  | < .001 |  |
| Group ✻ 3 Sess Block |  | 0.687 |  | 12 |  | 350.0 |  | 0.764 |  |
| Group ✻ Stimulus |  | 9.113 |  | 1 |  | 350.0 |  | 0.003 |  |
| 3 Sess Block ✻ Stimulus |  | 2.307 |  | 12 |  | 350.0 |  | 0.008 |  |
| Group ✻ 3 Sess Block ✻ Stimulus |  | 0.941 |  | 12 |  | 350.0 |  | 0.506 |  |
| Note. Satterthwaite method for degrees of freedom | | | | | | | | | |
|  | | | | | | | | | |

## Simple Effects

| Simple effects of Stimulus : Parameter estimates | | | | | | | | | | | | | | | | | |
| --- | --- | --- | --- | --- | --- | --- | --- | --- | --- | --- | --- | --- | --- | --- | --- | --- | --- |
| **Moderator levels** | |  | | | | | | **95% Confidence Interval** | | | |  | | | | | |
| **Group** | | **contrast** | | **Estimate** | | **SE** | | **Lower** | | **Upper** | | **df** | | **t** | | **p** | |
| 1 |  | AX- - A/X+ |  | -0.322 |  | 0.106 |  | -0.530 |  | -0.113 |  | 350 |  | -3.03 |  | 0.003 |  |
| 2 |  | AX- - A/X+ |  | -0.774 |  | 0.106 |  | -0.983 |  | -0.566 |  | 350 |  | -7.30 |  | < .001 |  |
| Note. Simple effects are estimated keeping constant other independent variable(s) in the model | | | | | | | | | | | | | | | | | |
|  | | | | | | | | | | | | | | | | | |

**Positive patterning discrimination**

| Fixed Effect Omnibus tests | | | | | | | | | |
| --- | --- | --- | --- | --- | --- | --- | --- | --- | --- |
|  |  |  |  |  |  |  |  |  |  |
|  | | **F** | | **Num df** | | **Den df** | | **p** | |
| group |  | 0.367 |  | 1 |  | 14.0 |  | 0.554 |  |
| 3Session Blocks |  | 0.812 |  | 12 |  | 350.0 |  | 0.638 |  |
| Stimulus |  | 172.854 |  | 1 |  | 350.0 |  | < .001 |  |
| group ✻ 3Session Blocks |  | 0.409 |  | 12 |  | 350.0 |  | 0.960 |  |
| group ✻ Stimulus |  | 20.082 |  | 1 |  | 350.0 |  | < .001 |  |
| 3Session Blocks ✻ Stimulus |  | 0.920 |  | 12 |  | 350.0 |  | 0.527 |  |
| group ✻ 3Session Blocks ✻ Stimulus |  | 0.287 |  | 12 |  | 350.0 |  | 0.991 |  |
| Note. Satterthwaite method for degrees of freedom | | | | | | | | | |
|  | | | | | | | | | |

## Simple Effects

| Simple effects of Stimulus : Parameter estimates | | | | | | | | | | | | | | | | | |
| --- | --- | --- | --- | --- | --- | --- | --- | --- | --- | --- | --- | --- | --- | --- | --- | --- | --- |
| **Moderator levels** | |  | | | | | | **95% Confidence Interval** | | | |  | | | | | |
| **group** | | **contrast** | | **Estimate** | | **SE** | | **Lower** | | **Upper** | | **df** | | **t** | | **p** | |
| 1 |  | BY+ - B/Y- |  | 0.772 |  | 0.126 |  | 0.524 |  | 1.02 |  | 350 |  | 6.13 |  | < .001 |  |
| 2 |  | BY+ - B/Y- |  | 1.570 |  | 0.126 |  | 1.322 |  | 1.82 |  | 350 |  | 12.47 |  | < .001 |  |
| Note. Simple effects are estimated keeping constant other independent variable(s) in the model | | | | | | | | | | | | | | | | | |
|  | | | | | | | | | | | | | | | | | |
